# Sex-specific responses in glucose-insulin homeostasis and lipoprotein-lipid components after high-dose supplementation with marine n-3 PUFAs in abdominal obesity: a randomized double-blind crossover study

**DOI:** 10.3389/fnut.2023.1020678

**Published:** 2023-06-19

**Authors:** Johnny Laupsa-Borge, Elise Grytten, Pavol Bohov, Bodil Bjørndal, Elin Strand, Jon Skorve, Jan Erik Nordrehaug, Rolf K. Berge, Espen Rostrup, Gunnar Mellgren, Simon N. Dankel, Ottar K. Nygård

**Affiliations:** ^1^Hormone Laboratory, Department of Medical Biochemistry and Pharmacology, Haukeland University Hospital, Bergen, Norway; ^2^Mohn Nutrition Research Laboratory, Department of Clinical Science, University of Bergen, Bergen, Norway; ^3^Department of Clinical Science, University of Bergen, Bergen, Norway; ^4^Department of Heart Disease, Haukeland University Hospital, Bergen, Norway; ^5^Mohn Research Center for Diabetes Precision Medicine, Department of Clinical Science, University of Bergen, Bergen, Norway

**Keywords:** sex-specific responses, omega-3 PUFAs, lipoprotein subfractions, blood lipids, glucose-insulin homeostasis, abdominal obesity, crossover design, constrained linear mixed-effects modeling

## Abstract

**Background:**

Clinical studies on effects of marine-derived omega-3 (n-3) polyunsaturated fatty acids (PUFAs), mainly eicosapentaenoic acid (EPA) and docosahexaenoic acid (DHA), and the plant-derived omega-6 (n-6) PUFA linoleic acid (LA) on lipoprotein-lipid components and glucose-insulin homeostasis have shown conflicting results, which may partly be explained by differential responses in females and males. However, we have lacked data on sexual dimorphism in the response of cardiometabolic risk markers following increased consumption of n-3 or n-6 PUFAs.

**Objective:**

To explore sex-specific responses after n-3 (EPA + DHA) or n-6 (LA) PUFA supplementation on circulating lipoprotein subfractions, standard lipids, apolipoproteins, fatty acids in red blood cell membranes, and markers of glycemic control/insulin sensitivity among people with abdominal obesity.

**Methods:**

This was a randomized double-blind crossover study with two 7-week intervention periods separated by a 9-week washout phase. Females (*n* = 16) were supplemented with 3 g/d of EPA + DHA (fish oil) or 15 g/d of LA (safflower oil), while males (*n* = 23) received a dose of 4 g/d of EPA + DHA or 20 g/d of LA. In fasting blood samples, we measured lipoprotein particle subclasses, standard lipids, apolipoproteins, fatty acid profiles, and markers of glycemic control/insulin sensitivity.

**Results:**

The between-sex difference in relative change scores was significant after n-3 for total high-density lipoproteins (females/males: −11%*/−3.3%, *p* = 0.036; *: significant within-sex change), high-density lipoprotein particle size (+2.1%*/−0.1%, *p* = 0.045), and arachidonic acid (−8.3%*/−12%*, *p* = 0.012), and after n-6 for total (+37%*/+2.1%, *p* = 0.041) and small very-low-density lipoproteins (+97%*/+14%, *p* = 0.021), and lipoprotein (a) (−16%*/+0.1%, *p* = 0.028). Circulating markers of glucose-insulin homeostasis differed significantly after n-3 for glucose (females/males: −2.1%/+3.9%*, *p* = 0.029), insulin (−31%*/+16%, *p* < 0.001), insulin C-peptide (−12%*/+13%*, *p* = 0.001), homeostasis model assessment of insulin resistance index 2 (−12%*/+14%*, *p* = 0.001) and insulin sensitivity index 2 (+14%*/−12%*, *p* = 0.001), and quantitative insulin sensitivity check index (+4.9%*/−3.4%*, *p* < 0.001).

**Conclusion:**

We found sex-specific responses after high-dose n-3 (but not n-6) supplementation in circulating markers of glycemic control/insulin sensitivity, which improved in females but worsened in males. This may partly be related to the sex differences we observed in several components of the lipoprotein-lipid profile following the n-3 intervention.

**Clinical trial registration:**

https://clinicaltrials.gov/, identifier [NCT02647333].

## 1. Introduction

The risk of cardiovascular disease (CVD), a leading cause of death worldwide (1), is lower in females than in males (2–4). Several lines of evidence indicate that this may partly be explained by sex-specific differences in the metabolism and levels of fatty acids (FAs), lipoproteins, lipids, and apolipoproteins (apo) due to the actions of sex hormones, genetic mechanisms, and other factors (4–9). Sex hormones may for example influence the metabolism of essential FAs and lead to sex-specific differences in levels of polyunsaturated fatty acids (PUFAs) in blood and cell membranes (6, 10, 11), and several genotype × sex interactions have been associated with the concentrations of total PUFAs and omega-3 (n-3) PUFAs in different blood lipid fractions (12, 13). Additionally, different measures of body adiposity have been related to blood levels of FAs, lipoprotein subfractions, and lipids in a sex-dependent manner (9, 14–17).

Elevated blood levels of triacylglycerols (TAGs) and TAG-rich lipoproteins (TRLs) or their remnants are strongly associated with abdominal obesity (18), atherosclerotic CVD (19–21), and all-cause mortality (19), and increasing cardiometabolic risk has been observed with increasing TAG levels within the widely accepted “normal” range (22, 23). The concentrations of different lipoprotein subclass particles, as well as their composition of lipids and proteins, including apoB and apoC-III, have been shown to be independent predictors of CVD comparable to or stronger than routinely measured levels of cholesterol in low-density lipoproteins (LDLs) and high-density lipoproteins (HDLs) (24–33). Premenopausal females are generally characterized by less atherogenic lipoprotein-lipid-apolipoprotein (LLA) patterns than similar-aged males (34–38). Cross-sectional analyses have shown less atherogenic lipoprotein subfraction profiles characterized by smaller-sized very-low-density lipoproteins (VLDLs), larger-sized LDLs, and/or larger-sized HDLs in females relative to males (16, 34–39). Studies have overall reported lower levels of large/medium VLDLs and higher concentrations of large/medium HDLs in females compared with males (16, 36–38, 40), as well as lower TAGs and higher HDL cholesterol (HDL-C) (14, 16, 34–38, 40–48), while the sex differences were more variable in other lipoprotein subfractions and lipids.

Lower CVD risk and cardiovascular mortality have been associated with higher intakes or blood levels of marine-derived n-3 PUFAs, mainly eicosapentaenoic acid (EPA) and docosahexaenoic acid (DHA) (49–56), and with higher intakes or circulating levels of plant-derived omega-6 (n-6) PUFAs, mainly linoleic acid (LA) (57–60). These inverse relationships have been partially explained by changes in blood fat fractions (58, 61, 62), primarily reduced TAG levels after n-3 intake (51, 63–67) and lower cholesterol levels after n-6 intake (62, 68–70). However, effects on CVD morbidity, mortality, and intermediate outcomes, including lipoproteins and lipids, have not been consistent across studies for n-3 (50, 53, 71–74), nor for n-6 (57, 59, 75–78), and it remains controversial whether dietary or supplemental PUFAs reduce cardiometabolic risk (70, 73, 79). Inconsistent results may partly be explained by differential responses to PUFA supplementation between sexes and other subgroups, and previous studies have suggested that n-3 and n-6 PUFAs modify CVD risk factors differently in females and males (80–82).

We previously reported that high-dose supplementation of high-quality oils with n-3 (EPA + DHA) or n-6 (LA) PUFAs was followed by reductions in primarily TAG-or cholesterol-related LLA components, respectively, and concluded that the responses after both interventions point to changes in the LLA profile that have been associated with reduced cardiometabolic risk, also among people with TAG or LDL cholesterol (LDL-C) levels within the normal range (83). However, to the best of our knowledge, no randomized controlled trials (RCTs) have previously explored sex-dependent lipoprotein subgroup responses after n-3 or n-6 PUFA interventions. Additionally, human studies have produced conflicting results regarding the effects of marine n-3 PUFAs on glucose responses and insulin sensitivity (84–89), which are important factors in the metabolism of LLA components (90–92), and findings for sex-dependent effects of n-3 PUFAs on measures of insulin resistance are inconsistent (86, 88). Notably, very few studies have investigated sexually dimorphic responses in glucose-insulin homeostasis after increased consumption of n-6 PUFAs (LA) (93), and none have directly compared females and males. Thus, we report here from a randomized crossover trial exploratory analyses of sex-specific responses in concentrations and sizes of all major lipoprotein subclass particles, determined by nuclear magnetic resonance (NMR) spectroscopy, and in levels of associated lipids and apolipoproteins, as well as in indices of glycemic control/insulin sensitivity, at baseline and after high-dose supplementation with n-3 or n-6 PUFAs in people with abdominal obesity. We demonstrate sex-dependent responses after n-3 (but not n-6) supplementation in glucose-insulin homeostasis that may be related to sex differences in several components of the lipoprotein-lipid profile following the n-3 intervention.

## 2. Materials and methods

This study reports exploratory analyses of sex-specific responses at baseline and after n-3 or n-6 PUFA interventions in an RCT with a crossover design previously described in detail (83). Briefly, the study was registered at ClinicalTrials.gov (NCT02647333) and conducted according to the guidelines in the Declaration of Helsinki. The trial procedures were reviewed and approved by the Regional Committee for Medical and Health Research Ethics (2014/2336/REK South-East). All participants provided written informed consent.

### 2.1. Participants

People with a sedentary lifestyle (<2 h/week of physical activity), 30–70 years of age, and abdominal obesity [waist circumference (WC) of ≥80 cm in females and ≥ 94 cm in males] but otherwise healthy, were screened for eligibility. Exclusion criteria were as follows: diagnosed diabetes, severe psychiatric illness, malabsorption disorders, or other chronic diseases; regular use of medications (except antibiotics, NSAIDs, antihistamines, diuretics, and hormone replacement therapy if prescribed by a physician); fasting serum levels of TAGs >5 mmol/L; cigarette smoking; alcohol abuse (>7 alcohol units/week for females; >14 alcohol units/week for males); previous coronary interventions; previous bariatric surgery; pregnancy or lactation; blood donation within 3 months before baseline; scheduled hospitalization during the study; and pacemaker or implantable cardioverter defibrillator (ICD). Participants taking dietary supplements, including fish oil, were asked to stop supplementation at least 3 months before pre-treatment baseline, except for those taking prescribed iron, calcium, or vitamin D for medical reasons. Changes in prescribed medication and supplementation during the study have been previously reported (83). Participants were instructed to maintain their usual lifestyle, dietary habits, and physical activity level during the study. The habitual food intake was obtained by dietary recordings from Monday to Sunday two times in the run-in period (baseline) and one time during both intervention periods (in week five). Participants were also questioned about their physical activity level at each baseline and follow-up visit, as previously described (83).

### 2.2. Study design

This randomized double-blind two-period crossover study was conducted at Haukeland University Hospital in Bergen, Norway, from May 2015 to March 2016. After a run-in period of 15 week without any dietary supplements, eligible participants were randomly assigned to one of two treatment sequences: supplementation with n-3 fatty acids (fish oil) in period one, followed by supplementation with n-6 fatty acids (safflower oil) in period two (sequence AB), or n-6 supplementation in period one, followed by n-3 supplementation in period two (sequence BA). The intervention periods lasted for 7 weeks and were separated by a 9-week washout phase. Participants were at pre-treatment baseline allocated to the sequences by stratified randomization using sex as a stratum. Both participants and study investigators were blinded to the intervention order until statistical analyses of all primary and most secondary outcomes were performed. Clinical measurements and tissue sampling were conducted 1 day before treatment period one started (baseline visit 1, B1; September 2015), the day after 7 weeks of the first intervention (follow-up visit 1, I1; November 2015), before treatment period two started (baseline visit 2, B2; January 2016), and after the second intervention (follow-up visit 2, I2; March 2016).

### 2.3. Interventions

The n-3 supplement was a hydrolyzed and re-esterified fish oil (TAG form) containing mainly EPA (C20:5n-3) and DHA (C22:6n-3), while the n-6 supplement was an organic, cold-pressed safflower oil containing mainly LA (C18:2n-6). Based on analyzed PUFA levels in these supplementation products, females should consume 5.6 mL/5.0 g (1.8 g EPA and 1.2 g DHA) and males 7.5 mL/6.7 g (2.4 g EPA and 1.6 g DHA) fish oil each day during the n-3 period to obtain a total dose with EPA + DHA of 3 and 4 g/d, respectively. In the n-6 period, the daily dose of safflower oil was for females 24.5 mL/22.6 g (15 g LA) and for males 32.7 mL/30.1 g (20 g LA). Different amounts of oils among females and males (a ratio of 3:4) allowed for similar intakes of EPA + DHA or LA per kg body weight. The total amount of supplement was divided into two daily dosages, one in the morning and one in the afternoon with meals, and the participants were instructed to take the supplement (using a syringe with the dose marked) in each period from the morning after the baseline visit to the evening before the follow-up visit. Both supplements were produced and sponsored by Pharmatech AS (Rolvsøy, Norway). The FA compositions of the oils were analyzed by the Lipid Research Group at the University of Bergen, while analyses of primary and secondary oxidation products and free fatty acids, as well as acid values, were performed by Multilab Østfold AS (Rolvsøy, Norway) and indicated high-quality products at delivery (83). Participants were instructed to store the bottles with oil supplements at 4°C in dark (i.e., in the refrigerator) during the entire intervention periods to reduce the rate of lipid oxidation. The trial was blinded for the participants and the study investigators by equal appearance of the study products and the bottles containing these oils. The taste was modified by adding 1.8% of a natural citrus aroma and 0.045% of steviol glycosides (E 960). Equal appearance was accomplished by using 0.85% calcium carbonate (E 170) and 0.2% turmeric (E 100). We have previously reported an analysis of the blinding, as well as compliance with the interventions and recorded adverse events (83).

### 2.4. Study visits

Prior to each study visit, participants were instructed to fast for at least 10–12 h and avoid alcohol for at least 48 h. Besides, they were asked to not exercise the day before, as this may also affect circulating levels of lipids and lipoproteins (94). In the morning between 07:30 and 11:30 a.m., venous blood samples were collected, and anthropometric variables were measured. The participants were also questioned about fasting status, alcohol intake, physical activity, stress level, use of medications and supplements, any health conditions, and compliance with the intervention since the last visit.

### 2.5. Anthropometrics

Body weight, height, and waist and hip circumferences were measured by standardized procedures, and body composition, i.e., body fat mass, body fat percent, visceral fat area, and fat-free mass, was further analyzed by using a segmental multifrequency bioelectrical impedance measurement system (InBody S10; InBody Co., Ltd., Seoul, South Korea) in a lying posture. Studies indicate that multifrequency bioelectrical impedance analysis is a simple and convenient method for estimating visceral fat area and significantly correlates with computed tomography scan measurements (95, 96), although its accuracy may vary depending on the population being measured (97). The means of two measurements were calculated for all the anthropometric measures.

### 2.6. Biochemical variables

#### 2.6.1. Fatty acid profiles in RBCMs

All blood samples were aliquoted and stored at −80°C until the end of the study when samples from all visits were analyzed at the same time. Fasting levels of 47 FAs in red blood cell membranes (RBCMs) were measured by gas–liquid chromatography using the internal standard C21:0 as previously described (83, 98). The total levels of analyzed n-3 and n-6 PUFAs are reported as the n-6/n-3 ratio, while the n-3 index was calculated as the sum of EPA and DHA in RBCMs and expressed as a percentage by weight of the total fatty acid (TFA) content (g FA/100 g TFA; wt%). We also report the levels of other RBCM FAs measured as wt% to eliminate differences in RBCM amounts, which may vary between individual blood sampling, even in the same person. However, since there is no agreement on the choice of FA measure (absolute vs. relative) (99–101), data on the concentrations (μg/mL) of RBCM FAs are also reported.

#### 2.6.2. Lipoprotein particle subclasses

Concentrations and sizes of lipoprotein particle subclasses, and a lipoprotein-based insulin resistance index (LP-IR), were determined in EDTA-plasma samples by LipoScience (now LabCorp, Inc., Raleigh, NC, United States) using an automated NMR spectroscopy assay according to the LipoProfile-3 algorithm (102, 103). The following nine subfractions were measured by NMR (estimated ranges of particle diameter): large VLDLs (including chylomicrons if present; >60 nm), medium-sized (medium) VLDLs (42–60 nm), small VLDLs (29–42 nm), intermediate-density lipoproteins (IDLs; 23–29 nm), large LDLs (20.5–23.0 nm), small LDLs (18–20.5 nm), large HDLs (9.4–14 nm), medium HDLs (8.2–9.4 nm), and small HDLs (7.3–8.2 nm). Total VLDL and HDL particle concentrations were calculated as the sum of small, medium, and large subclass particle concentrations, and total LDL particle concentration is the sum of small LDL, large LDL, and IDL concentrations.

#### 2.6.3. Lipids

We measured fasting serum TAGs, total cholesterol (TC), LDL-C, and HDL-C at the Department of Medical Biochemistry and Pharmacology (DMBP), Haukeland University Hospital, according to standardized procedures. Serum phospholipids, free cholesterol, and non-esterified fatty acids (NEFAs) were analyzed on a Hitachi 917 Chemistry Analyzer (Boehringer Mannheim GmbH, Mannheim, Germany) using kits from DiaSys Diagnostic Systems GmbH (Holzheim, Germany). Non-HDL cholesterol (non-HDL-C) was calculated by subtracting HDL-C from TC, while TRL cholesterol (TRL-C) was derived as non-HDL-C minus LDL-C.

#### 2.6.4. Lipoprotein (a) and apolipoproteins

Serum concentrations of lipoprotein (a) [Lp(a)], apoB, and apoA-I were analyzed at DMBP. Serum levels of apoA-II, apoC-II, apoC-III, and apoE were measured using the MILLIPLEX MAP Human Apolipoprotein Magnetic Bead Panel—Cardiovascular Disease Multiplex Assay (APOMAG-62 K; Merck Millipore, Billerica, MA, United States) and detected by the Bio-Plex 200 System (Bio-Rad, Hercules, CA, United States).

#### 2.6.5. Indices of glycemic control and insulin sensitivity

Fasting serum glucose, insulin, and insulin C-peptide (INCP), as well as glycated hemoglobin (HbA1c) in whole blood, were analyzed at DMBP. The homeostasis model assessment (HOMA) is reported as an index of insulin sensitivity, and we used the HOMA2 calculator developed by the University of Oxford^1^ to estimate insulin resistance (homeostasis model assessment of insulin resistance index 2, HOMA2-IR), insulin sensitivity (HOMA2-%S), and beta-cell function (HOMA2-%B) based on the updated computerized model (104). Fasting serum levels of glucose and INCP were used as inputs in these calculations. Another measure of insulin sensitivity, the quantitative insulin sensitivity check index (QUICKI), was calculated from the measurements of fasting glucose and insulin concentrations (105): 1/(log[fasting glucose (mg/dL)] + log[fasting insulin (μU/mL)]). We also report the revised version of QUICKI (rQUICKI), which incorporates the fasting level of NEFAs (106): 1/(log[fasting glucose (mg/dL)] + log[fasting insulin (μU/mL)] + log[fasting NEFAs (mmol/L)]). LP-IR, a marker of insulin resistance ranging from 0 (least) to 100 (most insulin resistant), was calculated by LipoScience as a weighted combination of lipoprotein subclass (large VLDLs, small LDLs, and large HDLs) and size (VLDL, LDL, and HDL sizes) parameters most closely associated with HOMA-IR (107).

#### 2.6.6. Other biomarkers

Alanine aminotransferase, albumin, alkaline phosphatase, aspartate aminotransferase, bile acids, bilirubin, creatine kinase, γ-glutamyl transpeptidase, lactate dehydrogenase, estrogen (17β-estradiol), testosterone, sex hormone-binding globulin (SHBG), thyroid stimulating hormone, free thyroxine, and insulin-like growth factor-1 were analyzed at DMBP. The free androgen index, often used as a surrogate marker of free testosterone, was calculated as (testosterone/SHBG) × 100. Serum 25-OH vitamin D_3_ was analyzed by liquid chromatography–tandem mass spectrometry at BEVITAL AS, Bergen.^2^ The ketone bodies 3-hydroxybutyrate and acetoacetate were measured by gas chromatography-tandem mass spectrometry at BEVITAL AS.

### 2.7. Statistical analyses

#### 2.7.1. Sex-specific responses in relative change scores (primary analysis)

The reported outcomes in this study are between-sex differences within treatments for relative change scores in fasting blood levels of LLA components: NMR-measured lipoprotein subclass particle concentrations and mean sizes, Lp(a), blood lipids (TAGs, NEFAs, phospholipids, TC, free cholesterol, LDL-C, and HDL-C), cholesterol levels calculated from these measures (non-HDL-C and TRL-C), apolipoproteins (apoB, apoA-I, apoA-II, apoC-II, apoC-III, and apoE), and ratios between these variables, as well as RBCM FAs. We also report between-treatment differences within females and males. The results presented here are derived from an intention-to-treat analysis including all randomized participants (16 females and 23 males), and the sample size calculation for the primary outcomes was previously reported (83).

Data are presented as raw unadjusted means (SDs), geometric means (1 SD ranges), or mean score differences (±absolute/relative effect estimates [95% CIs]). The geometric SD ranges used in descriptive statistics were calculated by dividing and multiplying the geometric means with the geometric SD factors to obtain the lower and upper limits, respectively (108). The distribution of data points from different measurements is shown by violin and error bar plots in Supplementary Figures S1–S4. All inferential tests were two-tailed with a nominal alpha level of 0.05. Adjustments for multiplicity were not performed due to the exploratory nature of the analyses and because a general adjustment method for mixed modeling of repeated measurements has not yet been developed due to difficulties associated with the correlation structure, which has to be taken into account (109). The statistical analyses were conducted with R v4.1.2,^3^ data transformation and exploration were done by using the *tidyverse* packages,^4^ and plots were made by the *ggplot2* package v3.3.0 and GraphPad Prism v.8.0.1 (GraphPad Software, San Diego, CA, United States).

Study outcomes were analyzed by period-and baseline-adjusted constrained linear mixed-effects models (cLMMs), a constrained longitudinal data analysis technique (110–114), with “subjects” as the random factor by using the *lme* function in the *nlme* package v3.1–157. We followed a design-driven approach and defined a fixed effects structure including “time,” “sex,” “period,” “treatment × time,” “time × sex,” and “treatment × time × sex,” chose a random effects structure with random intercepts and slopes for “time,” and used the correlation structure compound symmetry, which is appropriate for a crossover design (115). In case of heterogeneity, the model included a variance structure allowing for different variances per stratum of “treatment,” “time,” and/or “sex.” In the between-sex and between-treatment comparisons, the male sex and the n-6 intervention were defined as the reference groups, respectively.

By excluding the main term “treatment” and thereby constraining the baseline values to be equal across treatment groups, a reasonable assumption in RCTs, the cLMM inherently adjusts for baseline differences (111, 113, 114), i.e., period-specific baselines (psb; within-subject effect) in a crossover design. We also included subject-averaged baselines (sab; between-subject effect) as a fixed covariable to control for cross-level bias in this design (115). To obtain both baseline-adjusted within-treatment changes and between-treatment differences in change scores for each stratum of sex, we replaced the two “treatment × time” interaction terms in the cLMM with a time-varying covariable that describes the treatment effect. This computational trick was done by adding the new variable to the data in a long format and setting this covariable equal to the “time” variable for one treatment group and to zero (i.e., baseline) at all time points for the other (reference) group. When specified correctly, the intercept from the model output will be equal regardless of the chosen reference treatment group. Since the cLMM assumes that the baseline means are equal across arms, the new time-varying covariable essentially estimates the mean difference between treatments at the follow-up time, which is analytically equivalent to an ANCOVA approach (113). However, in contrast to this approach, the cLMM also provides baseline-adjusted within-treatment change scores.

In a separate model, we additionally adjusted for age and covariables that can differ during the trial (period level factors) or between sexes. Total energy intake normalized to body weight was included because of different amounts of oils between the intervention periods. Vitamin D_3_ was controlled for due to possible effects on blood lipids, such as LDL-C (116), and its often-varying tissue concentrations between seasons. Additionally, we adjusted for alcohol intake normalized to body weight (although participants were instructed to avoid alcohol for at least 48 h before study visits) and physical activity level (although we recruited people with a sedentary lifestyle), which impact LLA components (94, 117–120). To assess the effect of menopausal status, we fitted a separate model including a binary variable for menopausal status among females (0: premenopausal; 1: postmenopausal) and age categories among males (0: <55 years; 1: ≥55 years). Females were classified as postmenopausal based on the following criteria: follicle-stimulating hormone (FSH) ≥30 IU/L or age ≥ 55 years.

Values were transformed by the natural logarithm before analyses of responses in relative terms (108). Relative within-treatment changes from baseline to follow-up for each stratum of sex and between-sex differences in change scores within treatments are reported in the main text and tables as percentages calculated from the regression coefficients (i.e., the average of log-ratios) by the formula 100 × (exp^estimate^ − 1). In the figures, however, we show results in relative terms as sympercents (s%), of reasons previously explained (83). This relative measure is calculated as the difference between the natural logs of two numbers multiplied by 100, i.e.,100 × ln(a) – 100 × ln(b), making it straightforward to present and interpret without back transformation (121). As part of the model validation procedure, the Shapiro–Wilk test for normality, the D’Agostino test for skewness, and graphical tools (boxplots, quantile-quantile plots, and histograms) were used to assess the distribution of standardized residuals [see Supplementary material (Supplementary Text, *Materials and Methods*), for further details about the model validation procedure].

#### 2.7.2. Sex-specific responses in relative follow-up scores (sensitivity analysis)

When no outcome data are missing, the test for treatment difference over time in the cLMM is essentially equivalent to a test for treatment difference in an ANCOVA mixed model (AMM) (110, 111, 113, 114), which is often regarded as the most robust and powerful method (122–124). However, the cLMM is at least as efficient and powerful as the AMM (111–114). Yet, the results of cLMM slightly differ from the results of AMM because of the random part of the models (111, 114). Additionally, when outcome data are missing, the two analyses are based on different populations because participants with missing baseline or follow-up measurements are deleted in the AMM, while the cLMM uses all available data (111–114). We, therefore, conducted a sensitivity analysis of follow-up scores adjusted for the main effects of period, period-specific baselines, and subject-averaged baselines. This AMM included the fixed terms “treatment,” “sex,” “period,” “psb,” “sab,” and “treatment × sex,” a random effects structure with random intercepts-only, the correlation structure compound symmetry, and a data-driven variance structure.

#### 2.7.3. Missing data handling

Linear mixed-effects modeling has been shown to efficiently deal with data sets containing missing outcome values and to be the optimal estimator in trials with repeated outcome measurements comprising a large portion of missing data (125–127). Therefore, we did not pre-specify another strategy for dealing with potential intermittent missing repeated outcome measurements or missing data resulting from dropouts, such as multiple imputation before mixed modeling, as this has shown to add no obvious gain compared to a standard mixed model approach without imputed values (126, 127).

#### 2.7.4. Linear regression-determined associations

Sex-specific associations between continuous variables were analyzed with linear regression models by using the *lm* function in the R *stats* package v4.1.2. Before the analyses, data were transformed by the natural logarithm and standardized by the *scale* function in the R *base* package v4.1.2. The models included LLA components/secondary outcomes as univariate outcomes and anthropometric measures/biochemical variables as univariable predictors of interest. In one model, we adjusted for age. Another model was additionally adjusted for body mass index (BMI) and WC. We performed split sample analyses (sex-stratified multivariable models) and interaction analyses [augmented product term models (128)] to estimate within-sex associations and between-sex differences, respectively. From these models, we obtained two different effect sizes: (1) standardized regression coefficients (95% CIs), and (2) partial Cohen’s f^2^ by using the *partial_f2* function in the R package *sensemakr* v0.1.4. Cohen’s f^2^ ≥ 0.02 (2%), f^2^ ≥ 0.15 (15%), and f^2^ ≥ 0.35 (35%) represent a small effect/weak association, medium effect/moderate association, and large effect/strong association, respectively (129–131).

## 3. Results

### 3.1. Study participants

A flow diagram of the 39 randomized individuals (16 females and 23 males) included in the final intention-to-treat analysis is reported in Supplementary Figure S5 [CONSORT 2019 format (132)]. Pre-treatment clinical characteristics of the subjects randomly assigned to intervention sequence AB or BA are shown by sequence and period for each stratum of sex in Supplementary Table S1 (geometric means for biochemical variables) and Supplementary Table S2 (arithmetic means for biochemical variables). The study participants were at baseline middle-aged [females: 56.3 (SD 9.3, range 38–69) years; males: 55.2 (SD 9.5, range 37–69) years], overweight [BMI: 28.5 (SD 4.5) in females, 29.8 (SD 3.8) in males], had abdominal obesity [WC: 100 (SD 11.2) cm in females, 107 (SD 8.7) cm in males; visceral fat area: 134 (SD 51) cm^2^ in females, 183 (SD 61) cm^2^ in males], and were normolipidemic (*n* = 7; all males) or hyperlipidemic (*n* = 32; 16 males) (Supplementary Figure S6) (83). Among the females, we identified 10 (63%) to be postmenopausal at B1. None of the females with FSH levels <30 IU/L were 55 years or older, and none with FSH levels ≥30 IU/L were younger than 55 years. 12 (52%) of the males were 55 years or older.

The pre-treatment TAG levels were measured to be <1.13 mmol/L in 9 females and 8 males, ≥1.7 mmol/L in 2 females and 9 males, and ≥ 2.3 mmol/L in 1 female and 2 males. All females and males had at B1 an n-3 index in RBCMs higher than 4 wt%. According to proposed n-3 index risk zones for coronary heart disease (133), 4 females and 11 males were at intermediate (4–8 wt%) risk, while 12 females and 12 males were at low (>8 wt%) risk. In 3 females and 1 male, the n-3 index was >10 wt%. The mean B1 levels of LA in RBCMs were similar to the wt% of EPA + DHA (Supplementary Table S1) and lower compared with circulating levels of LA measured as wt% in erythrocyte phospholipids in seven prospective observational studies (59). The relative content of LA in RBCMs was measured to be >6 wt% in all participants, >8 wt% in 15 females and 18 males, and > 10 wt% in 1 female and 2 males. Thus, our sample was characterized by a relatively high EPA + DHA/LA ratio in blood in both females and males.

### 3.2. Dietary and supplemental oil intakes

The cross-sectional analyses of dietary data at the pre-treatment baseline showed no significant sex differences in absolute intakes of energy and macronutrients per unit of body weight or fat-free mass, except for a significantly higher fiber intake per unit of fat-free mass among females compared with males (Supplementary Table S3). During the n-3 intervention, the calculated average intake of supplemental EPA and DHA was in total 3.68 (SD 0.78) g/d in females and 4.04 (0.68) g/d in males (females vs. males [95% CI]: −0.35 g/d [−0.69, −0.013], *p =* 0.042; from linear regression). The average intake per 100 kg body weight was 4.63 (1.15) g/d in females and 4.21 (0.91) g/d in males (0.40 g/d [−0.08, 0.87], *p =* 0.101). When normalized to 100 kg fat-free mass, the average intake was 7.70 (1.81) g/d in females and 6.08 (1.15) g/d in males (1.48 g/d [0.82, 2.15], *p <* 0.001). During the n-6 period, the calculated average intake of supplemental LA was 13.7 (2.53) g/d in females and 17.7 (2.25) g/d in males (females vs. males [95% CI]: −3.92 g/d [−5.03, −2.81], *p <* 0.001). The average intake per 100 kg body weight was 17.2 (3.46) g/d in females and 18.4 (3.06) g/d in males (−1.24 g/d [−2.77, 0.28], *p =* 0.110). When normalized to 100 kg fat-free mass, the average intake was 28.1 (4.63) g/d in females and 26.7 (3.70) g/d in males (1.45 g/d [−0.58, 3.47], *p =* 0.160).

We observed non-significant reductions in energy intake from the habitual diet after both interventions (Table 1). However, the caloric contribution from the supplemental safflower oil resulted in significantly higher total energy intake after the n-6 intervention in both females and males. The significantly lower amount of supplemental PUFA oil during the n-3 periods compared with the n-6 periods, which was recorded to be on average 16 g/d (~144 kcal/d) less in females (n-3 vs. n-6 [95% CI]: −16.2 g/d [−18.4, −14.1], *p <* 0.001) and 22 g/d (~198 kcal/d) less in males (n-3 vs. n-6 [95% CI]: −22.1 g/d [−24.0, −20.3], *p <* 0.001), explains most of the approximately 170 less kcal that were consumed in total during the n-3 periods versus the n-6 periods in females (n-3 vs. n-6 [95% CI]: −170 kcal [−307, −32.9], *p =* 0.016) and males (−169 kcal [−287, −49.8], *p =* 0.006). However, and most importantly for our analyses of sex-dependent responses, changes from baseline in total energy intake per 100 kg body weight did not differ significantly between sexes after the n-3 (females vs. males [95% CI]: −31.1 kcal [−272, 210], *p =* 0.799) and n-6 (5.37 kcal [−234, 245], *p =* 0.965) interventions (Table 1). This was also the case when we normalized total energy intake to 100 kg fat-free mass.

**Table 1:** Recorded daily intakes of energy and macronutrients at baseline and during the intervention periods.^1^

Variable and treatment	Baseline^2^	Follow-up^3^	Absolute change^4^	Time^5^	wTXbSEX^6^	bTXwSEX^7^
Energy, diet, kcal						
n-3: females	1911 (356)	1831 (403)	−80.9 (−230, 68.6)	0.286	0.485	0.369
n-3: males	2,413 (521)	2,395 (554)	−10.2 (−143, 123)	0.880		0.603
n-6: females	1911 (356)	1857 (442)	−20.5 (−173, 132)	0.790	0.845	
n-6: males	2,413 (521)	2,364 (465)	−40.6 (−174, 92.3)	0.546		
Energy, total, kcal						
n-3: females	1911 (356)	1905 (406)	−6.29 (−157, 144)	0.934	0.684	0.016
n-3: males	2,413 (521)	2,427 (556)	+35.0 (−98.1, 168)	0.603		0.006
n-6: females	1911 (356)	2043 (448)	+163 (13.1, 314)	0.033	0.691	
n-6: males	2,413 (521)	2,608 (462)	+204 (72.5, 335)	0.003		
Energy, total/100 kg BW, kcal^8^						
n-3: females	2,459 (685)	2,420 (692)	−16.1 (−196, 164)	0.860	0.799	0.019
n-3: males	2,553 (689)	2,546 (738)	+15.1 (−145, 175)	0.853		0.027
n-6: females	2,459 (685)	2,574 (800)	+183 (2.95, 364)	0.046	0.965	
n-6: males	2,553 (689)	2,719 (682)	+178 (20.3, 336)	0.027		
Carbohydrate, E%						
n-3: females	39.8 (6.20)	39.0 (8.06)	−0.45 (−2.76, 1.86)	0.699	0.501	0.171
n-3: males	37.1 (7.18)	37.2 (7.12)	+0.60 (−1.45, 2.65)	0.563		0.025
n-6: females	39.8 (6.20)	38.2 (8.19)	−1.81 (−4.12, 0.50)	0.123	0.760	
n-6: males	37.1 (7.18)	35.4 (6.70)	−1.34 (−3.36, 0.68)	0.193		
Protein, E%						
n-3: females	16.7 (2.11)	16.5 (2.38)	−0.27 (−1.43, 0.90)	0.652	0.706	0.010
n-3: males	18.1 (3.98)	17.5 (3.53)	−0.56 (−1.59, 0.46)	0.279		<0.001
n-6: females	16.7 (2.11)	15.3 (1.72)	−1.51 (−2.67, −0.34)	0.012	0.385	
n-6: males	18.1 (3.98)	16.0 (3.40)	−2.19 (−3.20, −1.17)	<0.001		
Fat, E%						
n-3: females	37.0 (4.78)	39.4 (6.42)	+2.40 (−0.071, 4.87)	0.057	0.168	0.002
n-3: males	38.9 (5.94)	39.4 (5.50)	+0.090 (−2.10, 2.28)	0.935		<0.001
n-6: females	37.0 (4.78)	41.7 (6.49)	+5.24 (2.77, 7.72)	<0.001	0.588	
n-6: males	38.9 (5.94)	43.6 (5.37)	+4.34 (2.18, 6.51)	<0.001		
Fiber, E%						
n-3: females	2.05 (0.34)	2.06 (0.53)	+0.012 (−0.15, 0.18)	0.884	0.228	0.048
n-3: males	1.70 (0.31)	1.59 (0.27)	−0.12 (−0.27, 0.024)	0.099		0.320
n-6: females	2.05 (0.34)	1.91 (0.44)	−0.14 (−0.30, 0.029)	0.105	0.648	
n-6: males	1.70 (0.31)	1.52 (0.32)	−0.19 (−0.33, −0.043)	0.011		
Added sugar, E%						
n-3: females	5.43 (2.01)	4.15 (2.08)	−1.02 (−2.03, −0.017)	0.046	0.071	0.444
n-3: males	5.17 (4.45)	5.20 (4.08)	+0.21 (−0.68, 1.10)	0.637		0.239
n-6: females	5.43 (2.01)	4.58 (2.21)	−0.72 (−1.72, 0.29)	0.160	0.440	
n-6: males	5.17 (4.45)	4.77 (3.98)	−0.20 (−1.07, 0.68)	0.661		
Alcohol, E%						
n-3: females	4.57 (4.15)	3.09 (2.65)	−1.67 (−2.96, −0.37)	0.012	0.040	0.890
n-3: males	4.19 (3.40)	4.40 (4.26)	+0.15 (−0.99, 1.30)	0.793		0.105
n-6: females	4.57 (4.15)	2.98 (3.18)	−1.74 (−3.04, −0.44)	0.009	0.177	
n-6: males	4.19 (3.40)	3.53 (3.58)	−0.56 (−1.69, 0.58)	0.333		
SFAs, E%						
n-3: females	13.5 (2.18)	13.8 (2.75)	+0.37 (−0.90, 1.63)	0.567	0.242	0.004
n-3: males	14.9 (3.55)	14.4 (2.79)	−0.63 (−1.75, 0.48)	0.262		0.757
n-6: females	13.5 (2.18)	12.3 (2.74)	−1.12 (−2.38, 0.15)	0.083	0.684	
n-6: males	14.9 (3.55)	14.3 (3.35)	−0.77 (−1.87, 0.33)	0.168		
MUFAs, E%						
n-3: females	12.1 (2.55)	12.5 (3.42)	+0.34 (−0.85, 1.53)	0.569	0.323	0.138
n-3: males	13.5 (2.50)	13.1 (2.67)	−0.45 (−1.51, 0.60)	0.396		0.392
n-6: females	12.1 (2.55)	12.8 (2.42)	+1.08 (−0.12, 2.27)	0.076	0.147	
n-6: males	13.5 (2.50)	13.5 (2.54)	−0.089 (−1.13, 0.95)	0.865		
PUFAs, E%						
n-3: females	5.51 (1.47)	7.04 (1.62)	+1.43 (0.59, 2.27)	0.001	0.454	<0.001
n-3: males	5.70 (1.43)	6.76 (1.34)	+1.00 (0.26, 1.74)	0.008		<0.001
n-6: females	5.51 (1.47)	11.1 (2.01)	+5.66 (4.82, 6.50)	<0.001	0.527	
n-6: males	5.70 (1.43)	11.0 (1.83)	+5.30 (4.57, 6.03)	<0.001		
TRFAs, E%						
n-3: females	0.28 (0.088)	0.31 (0.14)	+0.023 (−0.042, 0.088)	0.487	0.188	0.007
n-3: males	0.35 (0.15)	0.32 (0.14)	−0.035 (−0.092, 0.022)	0.228		0.587
n-6: females	0.28 (0.088)	0.23 (0.11)	−0.058 (−0.12, 0.008)	0.082	0.404	
n-6: males	0.35 (0.15)	0.33 (0.14)	−0.021 (−0.077, 0.035)	0.458		
n-3 PUFAs, E%						
n-3: females	0.89 (0.30)	2.93 (0.97)	+2.02 (1.74, 2.31)	<0.001	0.204	<0.001
n-3: males	1.12 (0.38)	2.89 (0.74)	+1.78 (1.52, 2.03)	<0.001		<0.001
n-6: females	0.89 (0.30)	0.96 (0.37)	+0.050 (−0.24, 0.34)	0.730	0.241	
n-6: males	1.12 (0.38)	0.94 (0.36)	−0.18 (−0.42, 0.074)	0.165		
n-6 PUFAs, E%						
n-3: females	3.19 (0.95)	3.33 (1.11)	+0.091 (−0.53, 0.71)	0.772	0.343	<0.001
n-3: males	3.77 (0.86)	3.49 (0.91)	−0.31 (−0.86, 0.24)	0.270		<0.001
n-6: females	3.19 (0.95)	9.17 (1.76)	+5.99 (5.37, 6.61)	<0.001	0.524	
n-6: males	3.77 (0.86)	9.49 (1.58)	+5.72 (5.18, 6.26)	<0.001		
n-6/n-3 ratio						
n-3: females	3.79 (1.11)	1.22 (0.48)	−2.54 (−4.11, −0.97)	0.002	0.926	<0.001
n-3: males	3.62 (1.14)	1.29 (0.52)	−2.44 (−3.83, −1.05)	0.001		<0.001
n-6: females	3.79 (1.11)	11.0 (4.58)	+7.23 (5.66, 8.80)	<0.001	0.463	
n-6: males	3.62 (1.14)	11.6 (4.57)	+8.00 (6.64, 9.36)	<0.001		

^1^Pooled period data of total dietary and supplemental intakes of energy and macronutrients per day were analyzed with cLMMs. bTXwSEX, between-treatment within-sex; BW, body weight; cLMM, constrained linear mixed-effects model; E%, energy percentage of total energy intake; n-3, omega-3 PUFAs; n-6, omega-6 PUFAs; TRFAs, trans fatty acids; wTXbSEX, within-treatment between-sex.

^2^Values are arithmetic means (SDs) of dietary intakes recorded two times during seven consecutive days in the run-in period (baseline).

^3^Values are arithmetic means (SDs) of total dietary and supplemental intakes recorded over seven consecutive days during both intervention periods (in week five). One participant was lost to follow-up during the first intervention period in sequence AB.

^4^Absolute model-adjusted mean change scores (95% CIs) from baseline to follow-up.

^5^*P*-values for absolute changes from baseline to follow-up within treatments and sexes (time effects).

^6^*P*-values for absolute changes from baseline to follow-up within treatments and between sexes (sex differences in time effects). The first and second values refer to between-sex differences after the n-3 and n-6 interventions, respectively.

^7^*P*-values for absolute changes from baseline to follow-up between treatments and within sexes (group differences in time effects within each stratum of sex). The first and second values refer to between-treatment differences in females and males, respectively.

^8^Total dietary and supplemental energy intakes normalized to 100 kg body weight.

The energy percentage of fat increased significantly with the n-6 supplementation in both females and males compared with the n-3 intervention, and a significant decrease in protein intake was recorded after n-6 versus n-3 (Table 1). The changes in these nutrients, as well as carbohydrates and fiber, did not differ significantly between sexes after n-3 or n-6, nor when the intakes were normalized to 100 kg body weight, while we observed a significant between-sex difference in change scores for energy percentage of alcohol after n-3, also when normalized to body weight. Notably, the intakes of saturated, monounsaturated, and trans FAs did not change significantly with any treatment within both sexes and were not significantly different between sexes within treatments. Importantly, the recorded model-adjusted changes in total, n-3, and n-6 PUFA intakes were not significantly different between the sexes (Table 1). Additionally, the Likert scale data on physical activity level showed no significant differences between sexes at B1 (females vs. males [95% CI]: 3.49 [−2.05, 9.04], *p* = 0.224), and no significant sex differences in change scores after n-3 (3.99 [−0.62, 8.60], *p* = 0.089) or n-6 (0.40 [−4.22, 5.03], *p* = 0.863) supplementation.

### 3.3. FA profiles in RBCMs

We measured a comprehensive panel of 47 FAs in RBCMs because cardiometabolic effects of supplemental PUFAs are largely mediated through changes in the FA composition of different tissues, and circulating FA levels can reflect such changes (133, 134), as well as adherence to the interventions (99). The cross-sectional analyses of pre-treatment data showed that the contents of FAs in RBCMs differed significantly between sexes only for γ-linolenic acid (Table 2 and Supplementary Table S4). In the period-and baseline-adjusted mixed modeling, we found that the relative changes from baseline to follow-up differed significantly between sexes after the n-3 intervention in the n-6/n-3 ratio and wt% of arachidonic acid (AA) (more reduced in males than in females), while there were no significant sex-specific responses after n-6 supplementation for any of the variables (Table 3 and Supplementary Table S5). The relative content (wt%) of supplemental fatty acids in RBCMs increased less, although statistically non-significant, among females than males for EPA and DHA after the n-3 intervention, and for LA after n-6 supplementation. Furthermore, our analyses demonstrated significant between-treatment differences within both females and males for all the reported FAs, except α-linolenic acid. Notably, the observed changes in RBCM fatty acid profiles indicated good compliance with the interventions for both sexes. In the sensitivity analyses of follow-up scores adjusted for the main effects of period and baselines, we found after the n-3 intervention significant between-sex differences in the RBCM n-6/n-3 ratio (females vs. males [95% CI]: 7.83% [0.63, 15.5], *p =* 0.033) and wt% of EPA (−11.6% [−21.0, −1.11], *p =* 0.032), docosapentaenoic acid (−4.07% [−6.92, −1.14], *p =* 0.008), and AA (4.12% [1.21, 7.11], *p =* 0.007), but not DHA (−2.49% [−5.86, 0.99], *p =* 0.153), while no significant sex differences were observed after n-6 supplementation (Supplementary Table S6).

**Table 2:** Relative sex-specific differences at pre-treatment baseline in RBCM fatty acid levels.^1^

Variable and treatment	Females^2^	Males^2^	Relative difference^3^	*P*-value^4^
Relative contents of RBCM fatty acids				
n-6/n-3 ratio	2.37 (1.96, 2.86)	2.45 (2.00, 2.99)	−3.44 (−14.8, 9.42)	0.586
n-3 index, wt%	8.76 (7.52, 10.2)	8.28 (6.91, 9.93)	5.80 (−5.12, 18.0)	0.317
ALA, wt%^5^	0.19 (0.15, 0.25)	0.19 (0.16, 0.23)	−0.64 (−14.8, 15.9)	0.936
EPA, wt%	1.43 (1.03, 1.98)	1.31 (0.96, 1.81)	8.48 (−11.7, 33.3)	0.443
DPA, wt%	2.95 (2.69, 3.24)	3.08 (2.82, 3.38)	−4.20 (−9.66, 1.60)	0.161
DHA, wt%	7.30 (6.45, 8.28)	6.94 (5.88, 8.20)	5.24 (−4.41, 15.9)	0.305
LA, wt%	8.80 (8.21, 9.44)	8.73 (7.95, 9.59)	0.80 (−4.49, 6.37)	0.775
GLA, wt%	0.046 (0.036, 0.059)	0.056 (0.042, 0.075)	−18.5 (−31.9, −2.46)	0.032
DGLA, wt%	1.50 (1.31, 1.72)	1.65 (1.38, 1.96)	−8.96 (−17.8, 0.77)	0.078
AA, wt%	15.4 (14.0, 16.9)	15.1 (13.6, 16.7)	2.06 (−4.21, 8.73)	0.533
Concentrations of RBCM fatty acids				
TFAs, μg/mL	758 (701, 820)	742 (700, 788)	2.14 (−2.18, 6.66)	0.343
ALA, μg/mL^5^	1.43 (1.09, 1.88)	1.42 (1.19, 1.70)	0.89 (−13.7, 18.0)	0.912
EPA, μg/mL	10.8 (7.79, 15.0)	9.75 (7.23, 13.2)	10.8 (−9.12, 35.2)	0.317
DPA, μg/mL	22.4 (19.9, 25.3)	22.9 (20.8, 25.1)	−2.12 (−8.47, 4.67)	0.536
DHA, μg/mL	55.4 (47.6, 64.5)	51.5 (43.8, 60.6)	7.50 (−2.80, 18.9)	0.168
LA, μg/mL	66.7 (59.3, 75.1)	64.8 (57.8, 72.7)	2.95 (−4.42, 10.9)	0.448
GLA, μg/mL	0.34 (0.26, 0.44)	0.41 (0.30, 0.58)	−18.2 (−32.8, −0.35)	0.053
DGLA, μg/mL	11.4 (9.65, 13.4)	12.2 (9.97, 15.0)	−7.00 (−17.6, 4.96)	0.247
AA, μg/mL	117 (104, 131)	112 (98.8, 127)	4.25 (−3.45, 12.6)	0.295

^1^Fasting pre-treatment RBCM fatty acid levels were analyzed with GLS models adjusted for heterogeneity of variance by using the *gls* function in the R package *nlme* v3.1–157. Values were transformed by the natural logarithm before the analyses. AA, arachidonic acid; ALA, 𝛼-linolenic acid; DGLA, dihomo-γ-linolenic acid; DHA, docosahexaenoic acid; DPA, docosapentaenoic acid; EPA, eicosapentaenoic acid; GLA, γ-linolenic acid; GLS, generalized least squares; LA, linoleic acid; n-3, omega-3 PUFAs; n-6, omega-6 PUFAs; PUFAs, polyunsaturated fatty acids; RBCMs, red blood cell membranes; TFAs, total fatty acids; wt%, weight percentage of total fatty acids.

^2^Values are geometric means (1 SD ranges) of fasting RBCM fatty acid levels measured at the first baseline visit before any intervention.^6^

^3^Relative model-adjusted differences (95% CIs) between sexes (females vs. males) as percentages calculated from the model estimates: % = (exp^estimate^ − 1) × 100.^6^

^4^P-values for relative between-sex differences.

^5^One outlier was excluded from the analyses of ALA.

^6^Arithmetic means (SDs) and absolute differences between sexes are shown in Supplementary Table S4.

**Table 3:** Sex-specific responses in relative changes for RBCM fatty acid levels after 7 weeks of supplementation with n-3 or n-6 PUFAs.^1^

Variable and treatment	Baseline^2^	Follow-up^2^	Relative change^3^	Time^4^	wTXbSEX^5^	bTXwSEX^6^
Relative contents of RBCM fatty acids						
n-6/n-3 ratio						
n-3: females	2.36 (1.94, 2.87)	1.48 (1.24, 1.75)	−31.8 (−35.5, −27.9)	<0.001	0.027	<0.001
n-3: males	2.48 (1.99, 3.08)	1.44 (1.21, 1.71)	−37.2 (−40.1, −34.0)	<0.001		<0.001
n-6: females	2.12 (1.75, 2.56)	2.43 (2.07, 2.86)	+7.88 (4.51, 11.4)	<0.001	0.381	
n-6: males	2.26 (1.83, 2.79)	2.67 (2.27, 3.14)	+9.85 (6.87, 12.9)	<0.001		
n-3 index, wt%						
n-3: females	8.82 (7.45, 10.4)	12.5 (11.1, 14.2)	+35.7 (29.0, 42.7)	<0.001	0.054	<0.001
n-3: males	8.25 (6.78, 10.1)	12.6 (10.9, 14.5)	+44.8 (38.6, 51.2)	<0.001		<0.001
n-6: females	9.43 (8.14, 10.9)	8.78 (7.67, 10.0)	−3.06 (−5.82, −0.23)	0.034	0.414	
n-6: males	8.77 (7.41, 10.4)	8.02 (6.91, 9.30)	−4.55 (−6.89, −2.15)	<0.001		
ALA, wt%^7^						
n-3: females	0.18 (0.14, 0.23)	0.15 (0.12, 0.18)	−17.5 (−27.8, −5.71)	0.005	0.991	0.456
n-3: males	0.18 (0.14, 0.22)	0.15 (0.12, 0.19)	−17.4 (−26.0, −7.82)	0.001		0.186
n-6: females	0.19 (0.15, 0.24)	0.14 (0.11, 0.18)	−20.9 (−27.4, −13.9)	<0.001	0.733	
n-6: males	0.19 (0.15, 0.23)	0.14 (0.12, 0.16)	−22.4 (−27.7, −16.8)	<0.001		
EPA, wt%						
n-3: females	1.43 (0.96, 2.11)	4.03 (3.10, 5.24)	+137 (106, 171)	<0.001	0.090	<0.001
n-3: males	1.27 (0.88, 1.83)	4.22 (3.36, 5.29)	+176 (145, 210)	<0.001		<0.001
n-6: females	1.82 (1.32, 2.51)	1.35 (0.99, 1.84)	−10.7 (−18.5, −2.27)	0.015	0.948	
n-6: males	1.55 (1.12, 2.16)	1.13 (0.86, 1.47)	−10.4 (−17.2, −3.00)	0.007		
DPA, wt%						
n-3: females	2.93 (2.65, 3.23)	3.74 (3.47, 4.03)	+16.6 (13.2, 20.1)	<0.001	0.134	<0.001
n-3: males	3.05 (2.81, 3.31)	3.99 (3.75, 4.25)	+20.0 (17.0, 23.1)	<0.001		<0.001
n-6: females	3.23 (2.86, 3.65)	2.98 (2.74, 3.24)	−3.15 (−4.96, −1.31)	0.001	0.380	
n-6: males	3.34 (2.96, 3.78)	3.06 (2.82, 3.33)	−4.19 (−5.73, −2.62)	<0.001		
DHA, wt%						
n-3: females	7.34 (6.43, 8.36)	8.43 (7.80, 9.11)	+12.8 (8.44, 17.3)	<0.001	0.173	<0.001
n-3: males	6.95 (5.86, 8.25)	8.30 (7.40, 9.32)	+16.9 (13.0, 20.8)	<0.001		<0.001
n-6: females	7.57 (6.77, 8.46)	7.40 (6.65, 8.23)	−0.65 (−2.76, 1.51)	0.549	0.240	
n-6: males	7.18 (6.20, 8.31)	6.87 (5.95, 7.93)	−2.30 (−4.09, −0.49)	0.014		
LA, wt%						
n-3: females	8.76 (8.16, 9.39)	7.19 (6.56, 7.88)	−18.1 (−21.1, −15.1)	<0.001	0.971	<0.001
n-3: males	8.74 (7.99, 9.56)	7.22 (6.36, 8.19)	−18.2 (−20.8, −15.6)	<0.001		<0.001
n-6: females	8.72 (8.11, 9.37)	9.76 (9.29, 10.2)	+11.5 (7.46, 15.7)	<0.001	0.501	
n-6: males	8.67 (7.81, 9.64)	10.0 (9.10, 11.0)	+13.4 (9.83, 17.0)	<0.001		
GLA, wt%						
n-3: females	0.046 (0.035, 0.061)	0.026 (0.021, 0.032)	−43.3 (−53.2, −31.3)	<0.001	0.808	<0.001
n-3: males	0.053 (0.039, 0.073)	0.031 (0.021, 0.045)	−41.6 (−50.4, −31.2)	<0.001		<0.001
n-6: females	0.044 (0.032, 0.061)	0.045 (0.034, 0.060)	−0.46 (−11.7, 12.3)	0.939	0.613	
n-6: males	0.053 (0.041, 0.069)	0.055 (0.042, 0.073)	+3.64 (−6.43, 14.8)	0.489		
DGLA, wt%						
n-3: females	1.50 (1.32, 1.70)	1.12 (0.96, 1.31)	−24.5 (−28.5, −20.3)	<0.001	0.278	<0.001
n-3: males	1.65 (1.39, 1.97)	1.19 (0.98, 1.44)	−28.0 (−32.7, −23.0)	<0.001		<0.001
n-6: females	1.48 (1.29, 1.70)	1.49 (1.31, 1.69)	−0.034 (−3.64, 3.71)	0.986	0.353	
n-6: males	1.63 (1.37, 1.94)	1.68 (1.43, 1.98)	+2.21 (−0.75, 5.25)	0.144		
AA, wt%						
n-3: females	15.4 (14.0, 17.0)	13.8 (12.7, 15.1)	−8.25 (−10.4, −6.11)	<0.001	0.012	<0.001
n-3: males	15.2 (13.7, 16.8)	13.3 (12.1, 14.5)	−11.8 (−13.5, −10.0)	<0.001		<0.001
n-6: females	14.8 (13.4, 16.3)	15.2 (13.9, 16.6)	+0.58 (−1.72, 2.94)	0.620	0.860	
n-6: males	14.7 (13.3, 16.4)	15.2 (13.9, 16.6)	+0.86 (−1.11, 2.86)	0.393		
Concentrations of RBCM fatty acids						
TFAs, μg/mL						
n-3: females	770 (703, 844)	780 (713, 855)	+0.83 (−2.68, 4.47)	0.643	0.465	0.738
n-3: males	746 (705, 789)	742 (701, 786)	−0.82 (−3.45, 1.89)	0.548		0.888
n-6: females	778 (699, 865)	785 (711, 867)	+1.42 (−2.11, 5.08)	0.432	0.292	
n-6: males	751 (698, 808)	741 (700, 785)	−0.96 (−3.59, 1.74)	0.478		
ALA, μg/mL^7^						
n-3: females	1.34 (1.01, 1.76)	1.17 (0.90, 1.53)	−16.8 (−27.4, −4.60)	0.009	0.887	0.591
n-3: males	1.33 (1.08, 1.63)	1.11 (0.90, 1.36)	−17.8 (−26.5, −8.06)	0.001		0.141
n-6: females	1.47 (1.13, 1.90)	1.13 (0.90, 1.42)	−19.4 (−26.3, −11.9)	<0.001	0.357	
n-6: males	1.38 (1.08, 1.76)	1.03 (0.91, 1.17)	−23.6 (−29.0, −17.8)	<0.001		
EPA, μg/mL						
n-3: females	11.0 (7.63, 15.8)	31.5 (23.1, 42.8)	+156 (122, 196)	<0.001	0.091	<0.001
n-3: males	9.46 (6.64, 13.5)	31.3 (24.7, 39.7)	+200 (165, 239)	<0.001		<0.001
n-6: females	14.1 (9.88, 20.2)	10.6 (7.70, 14.6)	−6.81 (−19.2, 7.54)	0.331	0.813	
n-6: males	11.7 (8.40, 16.2)	8.34 (6.32, 11.0)	−8.81 (−19.4, 3.15)	0.141		
DPA, μg/mL						
n-3: females	22.6 (20.2, 25.3)	29.2 (25.5, 33.4)	+21.9 (15.7, 28.5)	<0.001	0.706	<0.001
n-3: males	22.7 (21.3, 24.3)	29.6 (27.6, 31.8)	+23.5 (18.2, 29.1)	<0.001		<0.001
n-6: females	25.1 (21.0, 30.1)	23.4 (20.8, 26.3)	−1.57 (−5.80, 2.85)	0.477	0.238	
n-6: males	25.1 (22.0, 28.6)	22.7 (20.8, 24.8)	−4.86 (−8.35, −1.24)	0.009		
DHA, μg/mL						
n-3: females	56.5 (49.0, 65.1)	65.8 (57.7, 75.0)	+14.1 (8.21, 20.3)	<0.001	0.443	<0.001
n-3: males	51.8 (44.2, 60.9)	61.6 (54.0, 70.3)	+17.2 (12.0, 22.6)	<0.001		<0.001
n-6: females	58.9 (49.7, 69.7)	58.1 (49.2, 68.6)	+0.65 (−4.53, 6.11)	0.808	0.268	
n-6: males	53.9 (46.1, 63.0)	50.9 (43.3, 59.9)	−3.20 (−7.45, 1.25)	0.155		
LA, μg/mL						
n-3: females	67.5 (58.8, 77.4)	56.1 (49.9, 63.2)	−17.0 (−21.3, −12.4)	<0.001	0.761	<0.001
n-3: males	65.1 (57.9, 73.2)	53.6 (46.8, 61.3)	−17.9 (−21.5, −14.0)	<0.001		<0.001
n-6: females	67.8 (59.8, 76.8)	76.6 (68.9, 85.1)	+13.2 (7.33, 19.5)	<0.001	0.900	
n-6: males	65.1 (56.5, 75.1)	74.2 (66.1, 83.3)	+13.8 (8.68, 19.1)	<0.001		
GLA, μg/mL						
n-3: females	0.35 (0.25, 0.48)	0.20 (0.15, 0.27)	−41.8 (−49.0, −33.7)	<0.001	0.986	<0.001
n-3: males	0.40 (0.28, 0.56)	0.23 (0.16, 0.34)	−41.7 (−48.8, −33.7)	<0.001		<0.001
n-6: females	0.35 (0.25, 0.48)	0.36 (0.26, 0.49)	+4.02 (−8.75, 18.6)	0.552	0.855	
n-6: males	0.40 (0.29, 0.56)	0.41 (0.31, 0.55)	+2.26 (−10.2, 16.4)	0.733		
DGLA, μg/mL						
n-3: females	11.6 (9.71, 13.8)	8.76 (7.40, 10.4)	−23.5 (−28.4, −18.2)	<0.001	0.158	<0.001
n-3: males	12.3 (10.1, 15.1)	8.80 (7.12, 10.9)	−28.1 (−32.0, −23.9)	<0.001		<0.001
n-6: females	11.5 (9.57, 13.8)	11.7 (9.74, 14.0)	+1.33 (−3.15, 6.01)	0.563	0.994	
n-6: males	12.2 (10.0, 15.0)	12.5 (10.3, 15.2)	+1.31 (−2.52, 5.29)	0.505		
AA, μg/mL						
n-3: females	119 (105, 134)	108 (96.5, 121)	−7.50 (−10.8, −4.04)	<0.001	0.024	<0.001
n-3: males	113 (99.6, 128)	98.4 (88.6, 109)	−12.5 (−15.2, −9.74)	<0.001		<0.001
n-6: females	115 (102, 130)	119 (105, 135)	+2.01 (−1.66, 5.82)	0.284	0.388	
n-6: males	111 (97.6, 126)	112 (102, 124)	−0.11 (−3.18, 3.05)	0.943		

^1^Fasting RBCM fatty acid levels were analyzed with cLMMs adjusted for the main effects of period and subject-averaged baselines. Values were transformed by the natural logarithm before the analyses. AA, arachidonic acid; ALA, 𝛼-linolenic acid; bTXwSEX, between-treatment within-sex; DGLA, dihomo-γ-linolenic acid; DHA, docosahexaenoic acid; DPA, docosapentaenoic acid; EPA, eicosapentaenoic acid; GLA, γ-linolenic acid; LA, linoleic acid; cLMM, constrained linear mixed-effects model; n-3, omega-3 PUFAs; n-6, omega-6 PUFAs; PUFAs, polyunsaturated fatty acids; RBCM, red blood cell membrane; TFAs, total fatty acids; wTXbSEX, within-treatment between-sex; wt%, weight percentage of total fatty acids.

^2^Values are geometric means (1 SD ranges) of fasting RBCM levels at baseline and follow-up within treatments and sexes.^8^

^3^Relative model-adjusted mean change scores (95% CIs) from baseline to follow-up as percentages calculated from the model estimates: % = (exp^estimate^ − 1) × 100.^8^

^4^*P*-values for relative changes from baseline to follow-up within treatments and sexes (time effects).

^5^*P*-values for relative changes from baseline to follow-up within treatments and between sexes (sex differences in time effects). The first and second values refer to between-sex differences after the n-3 and n-6 interventions, respectively.

^6^*P*-values for relative changes from baseline to follow-up between treatments and within sexes (group differences in time effects within each stratum of sex). The first and second values refer to between-treatment differences in females and males, respectively.

^7^One influential outlier was excluded from the final analyses of ALA.

^8^Arithmetic means (SDs) and absolute model-adjusted mean change scores are shown in Supplementary Table S5.

### 3.4. Lipoprotein particle subclasses

**Figure 1:**
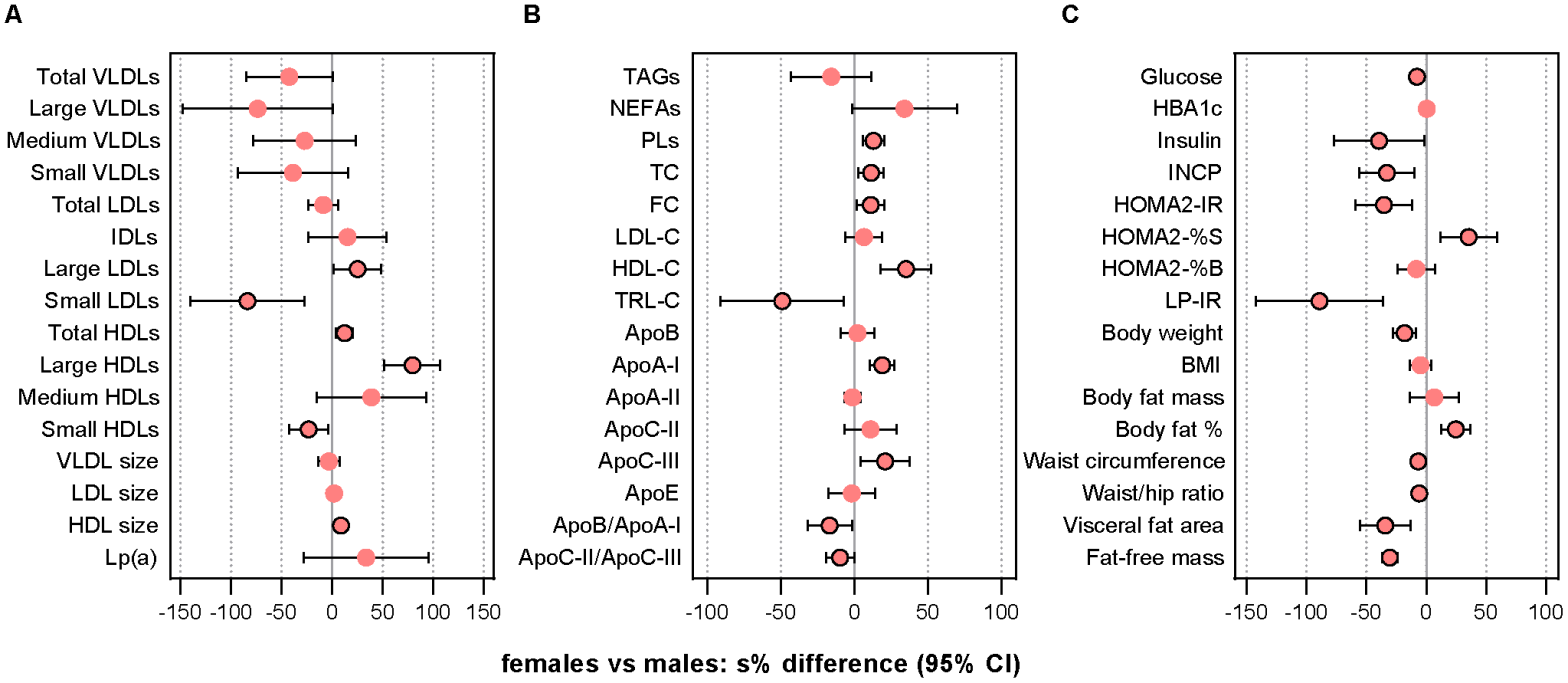
Comparison of pre-treatment characteristics. The dot plots show relative between-sex differences (females vs. males) at the pre-treatment baseline in lipoprotein subclass particle concentrations and mean sizes **(A)**, lipids and lipoproteins **(B)**, and indices of glycemic control/insulin sensitivity and anthropometric measures **(C)**. Data were analyzed with GLS models using the *gls* function in the R package nlme v3.1–157. In case of heterogeneity, the model included a variance structure allowing for different variances per stratum of “sex.” Before the analysis, values were transformed by the natural logarithm and multiplied by 100 to show differences as additive, symmetric percentages (sympercents; see main text). Error bars represent 95% CIs. Number of participants included: *n* = 39 (16 females, 23 males). Apo, apolipoprotein; FC, free cholesterol; GLS, generalized least squares; HbA1c, glycated hemoglobin; HDLs, high-density lipoprotein particles; HDL-C, HDL cholesterol; HOMA2-IR, homeostasis model assessment of insulin resistance index 2 (computer model); HOMA2-%B, homeostasis model assessment of 𝛽-cell function index 2 (computer model); HOMA2-%S, homeostasis model assessment of insulin sensitivity index 2 (computer model); IDLs, intermediate-density lipoprotein particles; INCP, insulin C-peptide; LDLs, low-density lipoprotein particles; LDL-C, LDL cholesterol; Lp(a), lipoprotein (a); LP-IR, lipoprotein-based insulin resistance index; PLs, phospholipids; s%, sympercent; TC, total cholesterol; TRL-C, TAG-rich lipoprotein cholesterol; VLDLs, vey-low-density lipoprotein particles. Total VLDLs and large VLDLs also include chylomicrons if present.

We found in the cross-sectional analyses of sex-specific differences at the pre-treatment baseline that several LLA components differed significantly between females and males (Figure 1 and Supplementary Tables S7, S8). An overall assessment of these variables indicates that females, compared with males, had a less atherogenic profile based on most of the LLA components with significant sex differences (more large LDLs and HDLs, fewer small LDLs and HDLs, more total HDLs, larger mean HDL size, higher HDL-C, lower TRL-C, more apoA-I, lower apoB/apoA-I ratio) but not all (more TC, more apoC-III, lower apoC-II/apoC-III ratio).

**Figure 2:**
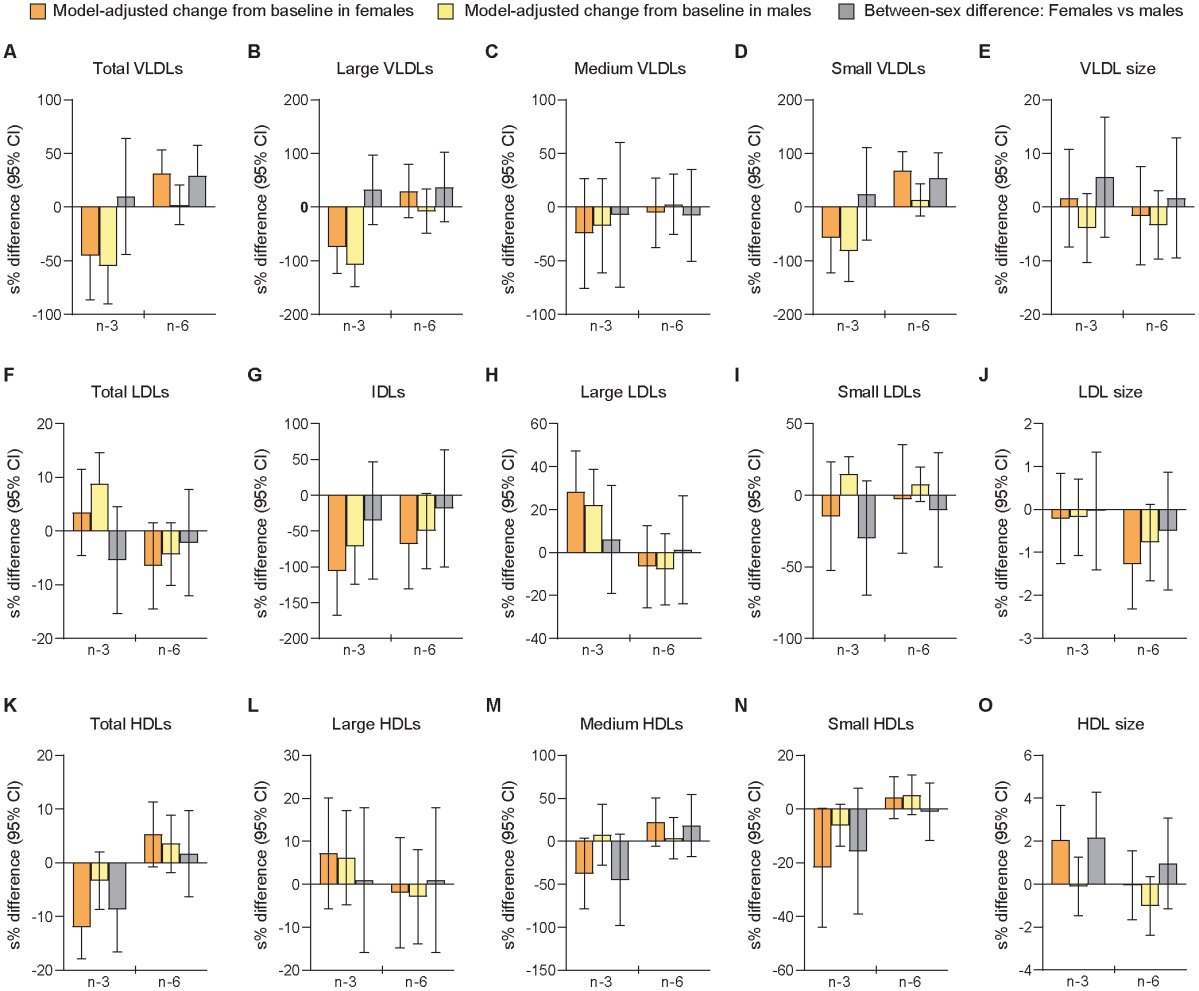
Differences between females and males in change scores for lipoprotein subfractions after 7 weeks of supplementation with n-3 or n-6 PUFAs. The bar plots show relative changes from baseline to follow-up and between-sex differences in change scores of particle concentrations and mean sizes for VLDLs **(A-E)**, LDLs **(F-J)**, and HDLs **(K-O)**. VLDLs and LDLs were measured in nmol/L, HDLs in μmol/L, and particle sizes in nm. Data were analyzed with period- and baseline-adjusted cLMMs (see main text). Before the analysis, values were transformed by the natural logarithm and multiplied by 100 to show within-sex changes and between-sex differences as additive, symmetric percentages (sympercents; see main text). Error bars represent 95% CIs. Relative changes from baseline to follow-up within females and males are shown in orange and yellow bars, respectively. Between-sex differences in relative change scores are shown in gray bars. Number of participants included: *n* = 39 (16 females, 23 males) at baseline in P1; *n* = 38 (16 females, 22 males) at follow-up in P1; *n* = 38 at baseline in P2 (16 females, 22 males); *n* = 38 at follow-up in P2 (16 females, 22 males). cLMMs, constrained linear mixed-effects models; HDL, high-density lipoprotein particles; IDLs, intermediate-density lipoprotein particles; LDLs, low-density lipoprotein particles; n-3, omega-3 PUFAs; n-6, omega-6 PUFAs; PUFAs, polyunsaturated fatty acids; P1, the first intervention period; P2, the second intervention period; s%, sympercent; VLDLs, very-low-density lipoprotein particles. Total VLDLs and large VLDLs also include chylomicrons if present.

The period- and baseline-adjusted mixed modeling showed that the relative changes from baseline to follow-up differed significantly between sexes after the n-3 intervention in total HDLs (females vs. males: −11.3% [−3.64 μmol/L] vs. −3.29% [−0.96 μmol/L], *p =* 0.036) and mean HDL size (+2.09% [+0.21 nm] vs. −0.093% [−0.006 nm], *p =* 0.045), and after n-6 supplementation in total VLDLs (+37.0% [+10.1 nmol/L] vs. +2.13% [+0.11 nmol/L], *p =* 0.041) and small VLDLs (+97.1% [+10.1 nmol/L] vs. +14.2% [+2.26 nmol/L], *p =* 0.021) (Figure 2 and Supplementary Tables S9, S10). Large but non-significant sex differences after mean changes in opposite directions for females and males were observed after n-3 supplementation in small LDLs (−13.7% [−46.1 nmol/L] vs. +16.3% [+94.3 nmol/L], *p =* 0.140) and medium HDLs (−31.2% [−2.99 μmol/L] vs. +7.96 [−0.34 μmol/L], *p =* 0.097), and after the n-6 intervention in large VLDLs (+34.9% [−0.16 nmol/L] vs. −7.17% [−1.25 nmol/L], *p =* 0.254). Smaller non-significant sex differences after mean changes in opposite directions were observed for mean VLDL size after n-3 (+1.69% [+0.55 nm] vs. −3.83% [−1.97 nm], *p =* 0.323) and for small LDLs after n-6 (−2.59% [+35.0 nmol/L] vs. +7.92% [+43.4 nmol/L], *p =* 0.611). In the analyses of between-treatment differences within sexes, we found that females and males differed for medium HDLs, LDL size, and HDL size, all of them demonstrating a significant difference between interventions only in females. Among these variables, only medium HDLs showed a between-sex difference after n-3 that was significantly different from the between-sex difference after n-6 (n-3 vs. n-6 in females [95% CI]: −44.9 [−62.9, −18.3], *p =* 0.003; n-3 vs. n-6 in males [95% CI]: 4.07 [−25.7, 45.7], *p =* 0.815; females vs. males after n-3 vs. n-6: −47.1 [−68.5, −11.1], *p =* 0.017).

The sensitivity analysis of follow-up scores adjusted for the main effects of period and baselines showed a significant between-sex difference in mean HDL size (females vs. males [95% CI]: 2.50% [0.57, 4.47], *p =* 0.012) after the n-3 intervention, but not in total HDLs (−5.66% [−11.6, 0.72], *p =* 0.079) after n-3 and not in total VLDLs (4.76% [−15.3, 29.5], *p =* 0.659) or small VLDLs (33.8% [−7.21, 93.0], *p =* 0.115) after n-6 (Supplementary Table S6). In contrast to the primary analysis, we found here a significant sex-specific difference in small LDLs after n-3 (−42.9% [−61.0, −16.5], *p =* 0.005). Moreover, large but non-significant sex differences were found for medium HDLs after n-3 (−39.2% [−64.7, 4.70], *p =* 0.072) and for small LDLs (−27.5% [−50.5, 6.05], *p =* 0.095) after n-6.

### 3.5. Lp(a) and standard lipids

**Figure 3:**
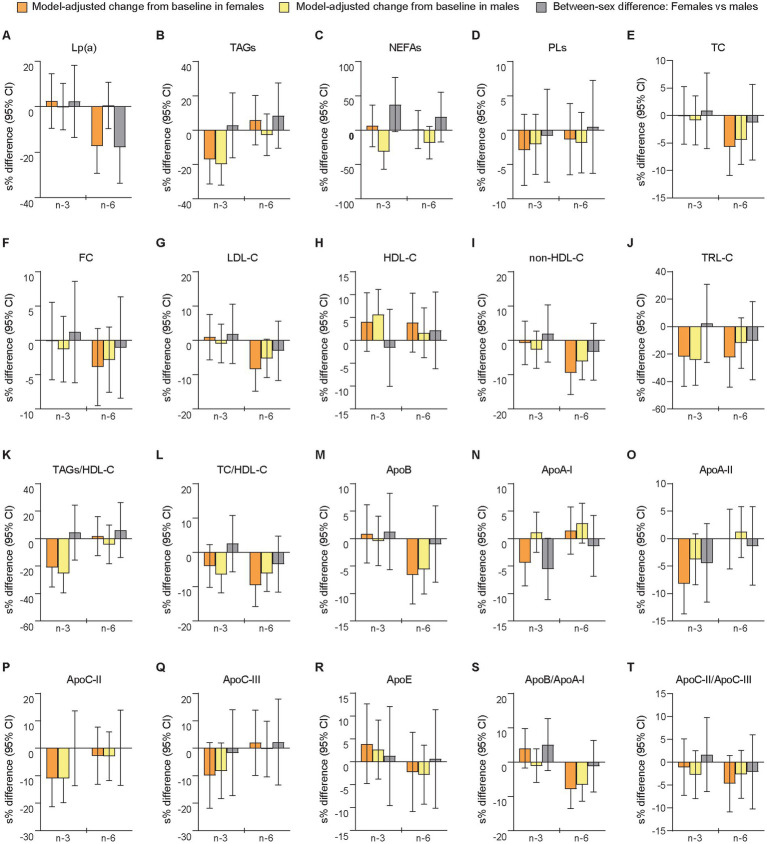
Differences between females and males in change scores for lipoprotein (a), blood lipids, and apolipoproteins after 7 weeks of supplementation with n-3 or n-6 PUFAs. The bar plots show relative changes from baseline to follow-up and between-sex differences in change scores for lipoprotein (a) **(A)**, blood lipids **(B–L)**, and apolipoproteins **(M–T)**. Blood lipids were measured in mmol/L and apolipoproteins in g/L. Apo, apolipoprotein; HDL-C, HDL cholesterol; LDL-C, LDL cholesterol; Lp(a), lipoprotein (a); non-HDL-C, non-HDL cholesterol; TC, total cholesterol; TRL-C, TAG-rich lipoprotein cholesterol. See further explanation and abbreviations in Figure 2.

The relative changes from baseline to follow-up differed significantly between sexes after the n-6 intervention in Lp(a) (females vs. males: −15.5% [−23.1 mg/L] vs. +0.52% [+8.60 mg/L], *p =* 0.028), while there were no significant sex-specific responses after n-3 supplementation (Figure 3 and Supplementary Tables S9, S10). However, a large but non-significant sex difference was observed in NEFAs (+6.43% [+0.030 mmol/L] vs. −26.7% [−0.10 mmol/L], *p =* 0.065) after mean changes in opposite directions for females and males following the n-3 intervention. Smaller non-significant sex differences after mean changes in opposite directions were observed after n-6 for TAGs (+5.92% [+0.026 mmol/L] vs. −2.64% [−0.080 mmol/L], *p =* 0.380) and NEFAs (+0.87% [+0.033 mmol/L] vs. −16.6% [−0.063 mmol/L], *p =* 0.301). The analyses of between-treatment differences within sexes demonstrated that females and males differed for Lp(a), TC, LDL-C, and non-HDL-C, all of which showed a significant difference between interventions only in females. Among these variables, only Lp(a) showed a between-sex difference after n-3 that was significantly different from the between-sex difference after n-6 (n-3 vs. n-6 in females [95% CI]: 21.9 [6.25, 39.8], *p =* 0.005; n-3 vs. n-6 in males [95% CI]: −0.37 [−11.4, 12.0], *p =* 0.950; females vs. males after n-3 vs. n-6: 22.3 [2.15, 46.5], *p =* 0.029). The sensitivity analysis showed significant between-sex differences in NEFAs (females vs. males [95% CI]: 51.3% [10.1, 108], *p =* 0.012) following the n-3 intervention and in Lp(a) (−11.6% [−21.8, −0.23], *p =* 0.046) after n-6 supplementation (Supplementary Table S6). Moderate to large but non-significant sex differences were found for NEFAs (25.3% [−9.02, 72.5], *p =* 0.162) and TRL-C (−15.4% [−33.9, 8.27], *p =* 0.177) after n-6. In the exploratory analyses of linear regression-determined associations, we found after the n-3 intervention that relative change scores in NEFA and TAG levels were inversely (non-significantly) associated and stronger in males (standardized regression coefficient [95% CI]: −0.38 [−0.81, 0.058], *p =* 0.086, f^2^ = 16.4%) than females (−0.098 [−0.67, 0.47], *p =* 0.717, f^2^ = 0.98%), while positive (non-significant) relationships were observed after n-6 in both females (0.052 [−0.52, 0.62], *p =* 0.850, f^2^ = 0.27%) and males (0.36 [−0.070, 0.80], *p =* 0.096, f^2^ = 15.3%).

### 3.6. Apolipoproteins

We found no significant between-sex differences for any of the apolipoproteins in relative or absolute change scores (Figure 3 and Supplementary Tables S9, S10), or in the sensitivity analysis of follow-up scores (Supplementary Table S6). Notably, we observed that females and males had changes in opposite directions for apoA-I (females vs. males: −4.25% [−0.059 g/L] vs. +1.16% [+0.010 g/L], *p =* 0.052) after the n-3 intervention (Supplementary Tables S9, S10). Additionally, the analyses of between-treatment differences within sexes demonstrated that only apoA-I differed for females (significant) and males (non-significant), although the between-sex differences after n-3 and n-6 were not significantly different (n-3 vs. n-6 in females [95% CI]: −5.66 [−9.88, −1.24], *p =* 0.013; n-3 vs. n-6 in males [95% CI]: −1.63 [−5.39, 2.29], *p =* 0.406; females vs. males after n-3 vs. n-6: −4.09 [−9.69, 1.85], *p =* 0.171).

Taken together, this exploratory analysis of sex-specific responses in change scores of LLA components after supplementation with n-3 or n-6 PUFAs showed in females compared with males relatively fewer total HDLs and larger mean HDL particle size after the n-3 intervention, and relatively more of total and small VLDLs after n-6 supplementation, as well as more AA in RBCMs after n-3 and fewer Lp(a) after n-6, while we found no significant sex differences for the apolipoproteins. The sensitivity analyses of follow-up scores supported this for mean HDL particle size, AA, Lp(a), and apolipoproteins, but not for total HDLs, total VLDLs, or small VLDLs. Additionally, after n-3 supplementation, large non-significant between-sex differences in relative change scores were observed for small LDLs (non-significantly decreased in females and significantly increased in males) and NEFAs (non-significantly increased in females and significantly decreased in males), while the analyses of follow-up scores showed significantly fewer small LDLs and more NEFAs in females than in males following this intervention. We also found in the sensitivity analyses significantly less EPA in RBCMs from females compared with males after n-3 supplementation. Notably, the analyses of between-treatment differences in change scores within sexes demonstrated for several LLA components significant differences between the n-3 and n-6 interventions only in females. Finally, covariable-adjusted effect estimates additionally controlled for covariables that can differ during the trial (period level factors) or between sexes, are reported in Supplementary material (Supplementary text, *Results*).

### 3.7. Glycemic control and insulin sensitivity

**Figure 4:**
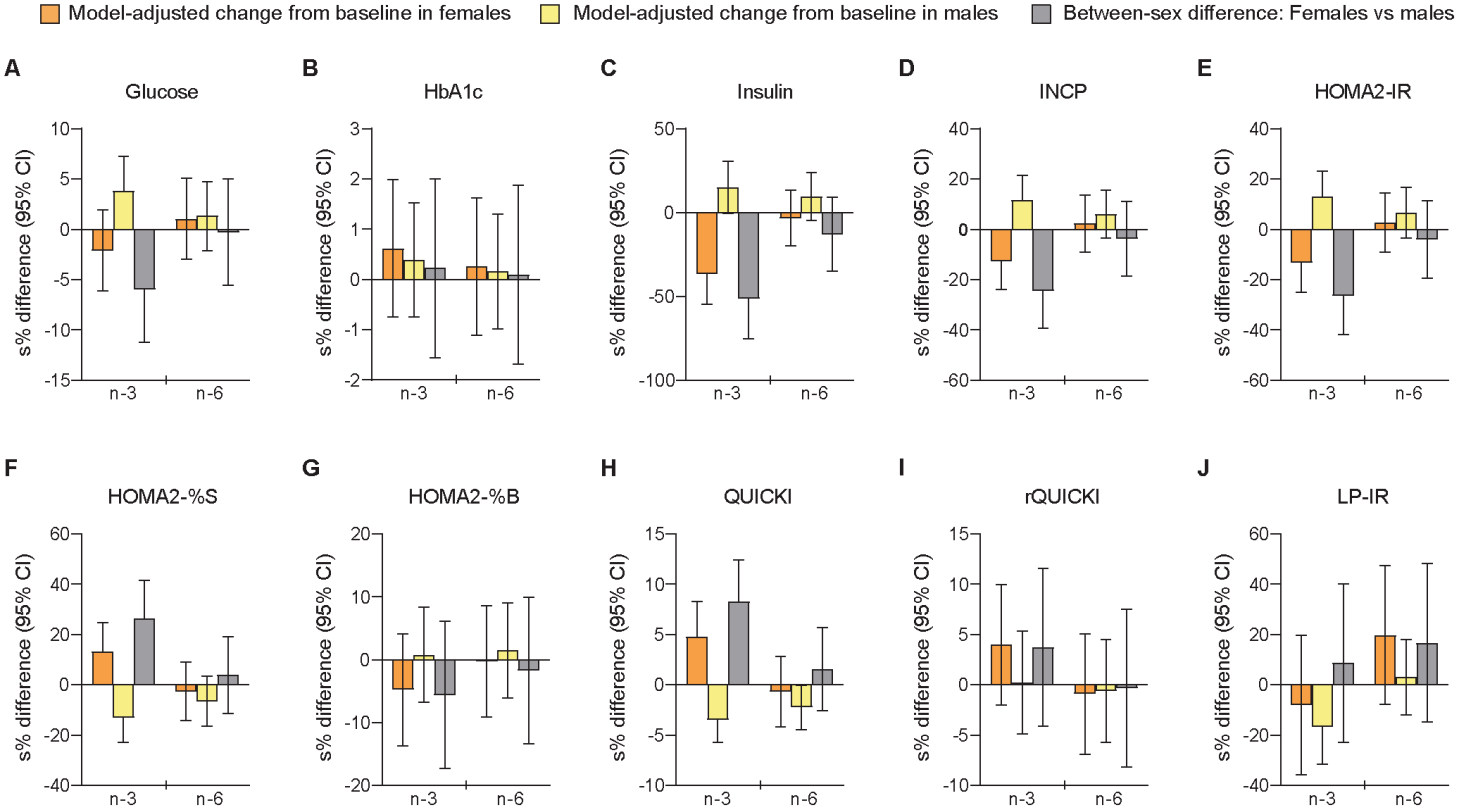
Differences between females and males in change scores for circulating markers of glycemic control/insulin sensitivity after 7 weeks of supplementation with n-3 or n-6 PUFAs. The bar plots show relative changes from baseline to follow-up and between-sex differences in change scores for glycemic control **(A,B)** and insulin sensitivity **(C–J)**. Glucose was measured in mmol/L, HbA1c in %, insulin in mU/L, and INCP in nmol/L. HbA1c, glycated hemoglobin; HOMA2-IR, homeostasis model assessment of insulin resistance index 2 (computer model); HOMA2-%B, homeostasis model assessment of 𝛽-cell function index 2; HOMA2-%S, homeostasis model assessment of insulin sensitivity index 2; INCP, insulin C-peptide; LP-IR, lipoprotein-based insulin resistance index; QUICKI, quantitative insulin sensitivity check index; rQUICKI, revised QUICKI. See further explanation and abbreviations in Figure 2.

We measured several indices of glycemic control and insulin sensitivity because differences in glucose-insulin homeostasis have shown to affect the metabolism and circulating levels of LLA components (90–92). The cross-sectional analyses of sex-specific differences at B1 showed that females compared with males had lower fasting glucose levels and higher insulin sensitivity (Figure 1 and Supplementary Tables S11, S12). Relative changes from baseline to follow-up differed significantly between sexes after the n-3 intervention for glucose (females vs. males: −2.07% [−0.12 mmol/L] vs. +3.90% [+0.21 mmol/L], *p =* 0.029), insulin (−30.5% [−2.48 mU/L] vs. +16.2% [+1.78 mU/L], *p <* 0.001), INCP (−11.8% [−0.077 nmol/L] vs. +12.5% [+0.084 nmol/L], *p =* 0.001), HOMA2-IR (−12.4% [−0.18] vs. +13.9% [+0.21], *p =* 0.001), HOMA2-%S (+14.2% [+13.2] vs. −12.2% [−8.60], *p =* 0.001), and QUICKI (+4.92% [+0.007] vs. −3.42% [−0.005], *p <* 0.001), while there were no significant sex-specific responses after n-6 supplementation (Figure 4 and Supplementary Tables S13, S14). The sensitivity analysis supported these findings (Supplementary Table S6). Thus, the results indicated that males were significantly more insulin resistant at baseline than females and that the circulating markers of glucose and insulin responses improved in females but worsened in males after n-3 supplementation. Notably, LP-IR, another measure of insulin resistance calculated from lipoprotein parameters associated with HOMA-IR (107), decreased after n-3 in both females (non-significantly) and males (significantly) and showed no significant between-sex difference. Besides, rQUICKI demonstrated no significant sex-dependent effect. Finally, in the analyses of between-treatment differences within sexes, we found that females and males differed for insulin, INCP, HOMA2-IR, HOMA2-%S, and QUICKI, of which all showed a significant difference between interventions only in females, and that the between-sex differences after n-3 were significantly different from the between-sex differences after n-6.

### 3.8. Anthropometrics

Body weight and composition were measured because the sexual dimorphism in anthropometric measures typically observed between females and males has been related to differences in glucose-insulin homeostasis and LLA components (9, 16, 18), and because we wanted to investigate if different anthropometric traits would be affected differently between sexes after high-dose PUFA supplementation. The cross-sectional analyses of pre-treatment data showed that females compared with males had higher body fat percent but less abdominal adiposity (Supplementary Tables S11, S12). No significant between-sex differences in relative or absolute change scores were observed (Supplementary Tables S13, S14), while the sensitivity analysis of follow-up scores showed significant sex differences in body weight (females vs. males [95% CI]: −1.28% [−2.25, −0.29], *p =* 0.013), BMI (−1.02% [−1.94, −0.093], *p =* 0.032), and body fat mass (−3.68% [−6.38, −0.91], *p =* 0.011) following the n-3 intervention (Supplementary Table S6). Notably, all anthropometric measures except fat-free mass changed after n-3 supplementation in opposite directions for females (decreased) and males (increased), while this was observed only for visceral fat area after the n-6 treatment (Supplementary Tables S13, S14). Additionally, small but significant increases in body weight and BMI were found among females and males after n-6 supplementation, probably related to the significantly higher total energy intake caused by this intervention, although the exploratory analyses of linear regression-determined associations did not show any significant relationships between relative changes in total energy intake and body weight for females (standardized regression coefficient [95% CI]: 0.28 [−0.15, 0.71], *p =* 0.182, f^2^ = 15.3%) or males (0.066 [−0.41, 0.54], *p =* 0.772, f^2^ = 0.48%).

### 3.9. Markers of liver function

The liver is a major regulator of glucose and lipid metabolism (135, 136), and circulating levels of liver enzymes may be related to hepatic lipid content (9, 137), which has been associated with hepatic production and secretion of TRLs (138–143). A standard liver panel was also part of the monitoring of potential adverse effects of the high-dose PUFA supplementation, which has been reported previously (83). All circulating markers of liver function (alanine aminotransferase, albumin, alkaline phosphatase, aspartate aminotransferase, bile acids, bilirubin, creatine kinase, γ-glutamyl transpeptidase, and lactate dehydrogenase) were at B1 found to be lower in females compared with males (Supplementary Tables S11, S12), indicating less hepatic lipid content in females compared with males (9, 137), possibly related to higher hepatic insulin sensitivity (9, 137, 144). We observed no significant between-sex differences in relative or absolute change scores in any of these measures after the PUFA interventions (Supplementary Tables S13, S14), while the sensitivity analysis showed a significant sex difference in γ-glutamyl transpeptidase (females vs. males [95% CI]: −15.8% [−28.6, −0.73], *p =* 0.041) after n-3 supplementation (Supplementary Table S6).

### 3.10. Additional hormones

Several hormones, including the sex steroids estrogen (17β-estradiol) and testosterone, were measured because they are known to affect the metabolism of n-3 and n-6 PUFAs (6), as well as the metabolism of LLA components (5, 8, 145). The secosteroid vitamin D_3_ was also measured due to possible effects on blood lipids, such as LDL-C (116). Our cross-sectional analyses of sex-specific differences at B1 showed, as expected, significantly lower testosterone levels in females than in males, higher SHBG, and lower free androgen index (Supplementary Tables S11, S12). Estrogen levels did not differ significantly, but it should be noted that both pre- and postmenopausal females were included in the study. Additionally, we found significantly lower free thyroxine levels in females than males, but no significant sex differences in thyroid stimulating hormone, insulin-like growth factor-1, or vitamin D_3_. None of the measured hormones showed significant between-sex differences in relative or absolute change scores (Supplementary Tables S13, S14), or in follow-up scores (Supplementary Table S6), after n-3 or n-6 supplementation. In the exploratory analyses of linear regression-determined associations, we found among males after the n-3 intervention a strong inverse relationship between relative changes of testosterone levels and the n-3 index (as well as in EPA and DHA) adjusted for age, BMI, WC, and SHBG (standardized regression coefficient [95% CI]: −0.62 [−1.06, −0.17], *p =* 0.009, f^2^ = 54.7%), while this was not evident among females (−0.022 [−0.90, 0.85], *p =* 0.955, f^2^ = 0.040%). We did not find any significant associations in relative change scores for testosterone versus LA after n-6, for estrogen versus n-3 index after n-3, or for estrogen versus LA after n-6.

### 3.11. Ketone bodies

Circulating levels of ketone bodies were measured as markers of hepatic beta-oxidation of FAs (146, 147), which has been shown to be affected by n-3 PUFAs (89). The fasting serum levels of 3-hydroxybutyrate and acetoacetate did not differ significantly between females and males at the pre-treatment baseline (Supplementary Tables S11, S12). These ketone bodies changed non-significantly after n-3 supplementation in opposite directions for females (increased) and males (decreased) and showed large but non-significant sex differences in relative change scores for 3-hydroxybutyrate (females vs. males: +13.1% [+16.5 μmol/L] vs. −25.8% [−27.8 μmol/L], *p =* 0.154) and acetoacetate (+17.1% [+10.3 μmol/L] vs. −11.2% [−7.69 μmol/L], *p =* 0.254) (Supplementary Tables S13, S14), and in follow-up scores (Supplementary Table S6). After the n-6 intervention, these ketones were non-significantly lowered in both females and males and demonstrated no significant sex-specific responses. Finally, the exploratory analyses of linear regression-determined relationships showed after adjustment for age, BMI, and WC strong positive associations between relative changes in 3-hydroxybutyrate and NEFA levels after the n-3 intervention for females (standardized regression coefficient [95% CI]: 0.85 [0.54, 1.16], *p <* 0.001, f^2^ = 322%) and males (0.75 [0.40, 1.11], *p <* 0.001, f^2^ = 118%), and after n-6 supplementation for females (0.64 [0.22, 1.06], *p =* 0.006, f^2^ = 103%) and males (0.56 [0.16, 0.96], *p =* 0.009, f^2^ = 50.8%). Similar associations were found for acetoacetate.

## 4. Discussion

Although biological sex is an important modifier of cardiometabolic risk factors (7, 9), including body adiposity, glucose-insulin homeostasis, blood fat fractions, and LLA profiles (5, 8–11, 18, 35, 36, 38, 91, 144, 145, 148–154), and several of these factors have shown sex-specific responses to dietary changes (5, 9, 45, 155), we have lacked analyses of sexual dimorphism in cardiometabolic risk following increased consumption of n-3 or n-6 PUFAs. We here report sex-dependent responses after PUFA interventions in LLA components, glycemic control, and insulin sensitivity based on data that have directly compared females and males using n-3 (EPA + DHA) or n-6 (LA) PUFA supplementation with high-quality oils and similar doses per kg body weight across sexes. In our analyses, we observed sex-dependent responses (females vs. males) following n-3 supplementation for small LDLs (fewer), total HDLs (fewer), mean HDL particle size (larger), and NEFAs (more), as well as EPA (less) and AA (more) in RBCMs, and after n-6 supplementation for small VLDLs (more), total VLDLs (more), and Lp(a) (fewer). Importantly, we found after the n-3 (but not n-6) intervention significant sex differences in circulating markers of glycemic control/insulin sensitivity, which improved in females but worsened in males.

The significant changes in markers of insulin sensitivity among females after n-3 supplementation are in line with a systematic review and meta-analysis of 31 RCTs, showing that interventions with n-3 PUFAs (α-linolenic acid, EPA, and/or DHA) for at least 6 weeks significantly improve indices of insulin resistance in females (86). Also, in two cohorts of people with overweight/obesity, the n-3 status (EPA + DHA) in erythrocytes or plasma phospholipids was inversely associated with type 2 diabetes in females (156, 157). In contrast to our findings, the meta-analysis and cohort studies did not demonstrate significant effects or relationships in males, and a previous trial in males with overweight showed that a higher n-3 index was associated with increased insulin sensitivity (84). Notably, none of the included studies in the meta-analysis directly compared females and males. In a more recent 12-week parallel-arm study specifically investigating sex-dependent effects of a DHA-enriched fish oil compared with corn oil, fasting insulin levels and HOMA-IR were significantly reduced in adults with abdominal obesity, but no significant sex differences were evident (88). In our trial, the significant increase in fasting glucose levels observed among males after the n-3 intervention agrees with a study in healthy males that found after 8 weeks of n-3 supplementation significantly elevated fasting and postprandial plasma glucose concentrations but no significant changes in insulin levels (89), while the DHA-enriched fish oil study showed no increase in fasting glucose levels and no sex-dependent effects (88). Although it is questionable, within the context of short-term increased n-3 PUFA intake, to extrapolate our observed changes in glucose and insulin markers to sex-specific effects on CVD endpoints, previous research has shown that fasting glucose, fasting insulin, and HOMA-IR were positively associated with incident CVD (158–161). A prospective cohort study in males found an odds ratio of 1.6 (95% CI: 1.1–2.3) for ischemic heart disease with each increase of 1 SD in fasting insulin concentration after adjustment for plasma TAGs, apoB, LDL-C, and HDL-C (158), while a meta-analysis of cohort studies and nested case–control studies showed that the relative risk of CVD per 1 SD increase in HOMA-IR was 1.37 (1.05, 1.80) for females and 1.41 (1.12, 1.77) for males (159).

Possible mechanisms explaining how n–3 PUFAs may affect insulin sensitivity have been previously described (82, 86, 162, 163), but reasons behind the sex differences we observed in fasting glucose levels and insulin sensitivity after the n-3 intervention are not clear (9, 82, 86, 89). Available data indicate that the sexual dimorphism may be caused or mediated, at least partly, by changes in steroid receptor signaling pathways (8, 164, 165). Previous n-3 PUFA intervention studies have shown inconsistent results regarding responses in sex hormones, including testosterone levels in males (166, 167). The n-3 intervention in the current study was not followed by significant changes or sex differences in testosterone levels or free androgen index, but we found among males a strong inverse association between relative changes in the n-3 index and testosterone levels adjusted for age, BMI, WC, and SHBG. In contrast, a DHA-enriched fish oil intervention (860 mg DHA and 120 mg EPA/d) for 12 weeks showed in males that change in testosterone was positively correlated with change in DHA in erythrocyte membranes after controlling for age and BMI (167).

Regardless of sex hormone levels, higher intakes of EPA and DHA may decrease the expression of the androgen receptor (AR) (168, 169) or modulate the actions of AR (170). This nuclear transcription factor plays a major role in glucose-insulin homeostasis (164, 171), potentially leading to sexually dimorphic effects on glucose, lipid, and energy metabolism in females and males (164, 165, 172). Additionally, the activity of 5-alpha-reductase, which catalyzes the conversion of testosterone to the more biologically active form dihydrotestosterone (DHT), may also be hampered by n-3 PUFAs and EPA’s 15-lipoxygenase metabolite (173). Furthermore, AR-induced activity has shown to be inhibited by small heterodimer partner (SHP) (174, 175), and animal data indicate that fish oil feeding increases the hepatic expression of SHP (176). This nuclear receptor regulates genes involved in, e.g., glucose and lipid metabolism (177–179) and may have sex-dependent and tissue-specific functions leading to disparate effects on TAG metabolism (180). Notably, the bidirectional modulation of glucose, lipid, and energy metabolism by testosterone, DHT, and AR-induced activity in females and males are among the most important sexually dimorphic aspects of metabolic regulation (164, 172, 181). While a reduction in testosterone, DHT, and/or AR signaling has been associated with abdominal obesity, hyperglycemia, insulin resistance, leptin resistance, metabolic syndrome, and type 2 diabetes in males, it has been related to an improved metabolic phenotype in females (164, 172, 181–184).

We observed similar reductions in TRLs, TAGs, and TRL-C in both sexes after the n-3 intervention, but the mechanisms may have been different in females and males because of the sexual dimorphism in glucose-insulin homeostasis, possibly affecting the balance between hepatic secretion, peripheral lipolysis, and vascular clearance of TRLs. Several lines of evidence have shown that NEFAs contribute the largest fraction to VLDL-TAG production in both the fasted and fed states in different metabolic conditions (185–187), yet indirectly via a cytosolic TAG pool (188–190). It has also been proposed that the TAG-lowering effect of n-3 PUFAs is best explained by an effect on the NEFA pool (187), which, at least in obesity, may partly occur via suppression of adipose tissue inflammation and thereby reducing intracellular lipolysis in adipocytes mediated by adipose triacylglycerol lipase and hormone-sensitive lipase. Recent experimental evidence from an animal model of aging, where rats received a fish oil intervention, point to potential mechanisms involved in n-3 PUFA-induced changes in adipose tissue function, inflammation, and lipolysis, and circulating lipid levels (191). This study showed that the expression of several proteins involved in lipid metabolism, notably ER lipid raft-associated protein 1 (Erlin1) and fatty acid binding protein 1 (Fabp1), were significantly changed (down-regulated Erlin1 and Fabp1) after the fish oil-derived n-3 PUFA intervention. However, the spatiotemporal sequence of events from hepatic NEFA influx to VLDL efflux is complex and incompletely understood (92, 138, 190, 192–195), and not all studies showing lowered TAGs concurrently found a significant reduction in NEFAs (89). While NEFAs in the present study increased non-significantly in females and decreased significantly in males after n-3 supplementation, TAG levels were significantly reduced in both sexes and to a similar degree, in contrast to a previous study of healthy adults showing a greater TAG-lowering effect of EPA + DHA in males compared with females (45).

In the present study, elevated insulin levels in males after the n-3 intervention probably reduced the NEFA flux from adipose tissues to the liver (196–198), as insulin suppresses the activity of adipose triacylglycerol lipase and hormone-sensitive lipase in insulin-sensitive states. By blocking lipolysis in adipocytes, as well as restraining intrahepatic ketogenesis and enhancing peripheral ketone body clearance, insulin also lowers circulating ketone body concentrations (146, 147, 199). This may explain the large (non-significant) sex differences in fasting serum levels of ketone bodies (increased in females and decreased in males), possibly reflecting sexual dimorphism in hepatic beta-oxidation of FAs and ketogenesis after n-3 supplementation. Additionally, we observed after the n-3 intervention that measures of body adiposity changed (non-significantly) in opposite directions among females (decreased) and males (increased), which may be related to sexual dimorphism in glucose and insulin responses.

A widely held hypothesis, which was initially based on short-term experiments with acute insulin infusions (194, 196, 200, 201), is that insulin inhibits hepatic production and secretion of larger, TAG-rich VLDLs (VLDL1) (141, 189, 202), and that this effect is partly lost if the liver becomes insulin resistant, leading to increased hepatic VLDL1-TAG output (139–141, 202–204), despite efficient suppression of serum NEFAs (205). However, others have questioned that hypertriacylglycerolemia is due to hepatic insulin resistance (196). Studies have demonstrated that higher insulin signaling in the long term (chronic hyperinsulinemia) does not inhibit but rather stimulates hepatic VLDL1-TAG synthesis and secretion, even when NEFA levels are lowered (138, 192–194, 196). The stimulation of *de novo* lipogenesis, decreased FA oxidation, and increased TAG esterification associated with chronic hyperinsulinemia presumably override the effects of reduced NEFA flux to the liver (194). Other lines of evidence point to the presence of differential insulin resistance between tissues or metabolic pathways within the liver, in which insulin fails to suppress gluconeogenesis but continues to activate lipogenesis, thereby increasing the hepatic output of both glucose and TAGs (206), although recent animal and human data have questioned the importance of selective hepatic insulin resistance (207, 208). Furthermore, elevated glucose levels have been shown to decrease FA oxidation and increase hepatic TAG synthesis and secretion (194, 209–212), of which a large proportion may originate from other sources than plasma NEFAs in hyperglycemic hyperinsulinemic individuals (194, 209). In an analysis of factors that predicted overproduction of TRLs, only liver fat and plasma glucose were identified as significant predictors in the multiple regression modeling (140). Importantly, recent experiments with primary hepatocytes, involving diverse *in vivo* and *in vitro* assays, have shown that insulin enhances in the fed state VLDL1-TAG production and secretion via the phosphatidic acid (PA) pathway (92, 213). This response is downregulated when fasting causes insulin levels to drop, thereby preventing the release of TAGs into an already NEFA-rich bloodstream in the fasted state (195). These experiments showed that higher insulin signaling elevated dramatically in lipid droplets the content of PA (generated by phospholipase-D1 after its activation by ADP-ribosylation factor 1), and that PA signaling induced transport of lipid droplets to smooth endoplasmic reticulum inside hepatocytes, where lipid droplets were catabolized to produce lipoproteins (92, 213).

Based on these lines of evidence, and other supporting studies (189, 190, 214, 215), it is tempting to speculate that the higher fasting glucose and insulin levels observed in the current study among males after n-3 supplementation, possibly in part caused by modulated AR expression or actions, (1) reduced the NEFA flux from adipose tissues, but (2) enhanced the output of hepatic VLDL1-TAG, possibly recruited at least in part from an intracellular phospholipid pool via the PA pathway (190, 214), such as PA-rich activated LDs (92, 213), and (3) stimulated lipolytic conversion of TRLs and subsequent FA uptake and accumulation of TAGs in peripheral tissues by glucose-, insulin-, and eventually n-3 PUFA-stimulated actions of lipoprotein lipase (LPL) (198, 210, 216–221). These effects would ultimately lead to reduced circulating TRL, TAG, and NEFA levels, as well as ketone bodies, and increased adiposity. In females, the TAG-lowering effects of n-3 PUFAs may be partly explained by reduced hepatic secretion of TRLs involving mechanisms normally attributed to EPA and DHA, i.e., less available substrates for TAG synthesis, decreased activity of TAG-synthesizing enzymes, and/or stimulated degradation of apoB-100 (49, 187, 222–227). It is known that females, compared with males, secrete more TAG-rich VLDLs from the liver (228). This is matched with accelerated VLDL1-TAG clearance rates, collectively contributing to lower plasma VLDL1-TAG levels in obesity in females (228). If n-3 PUFAs increase the hepatic expression of SHP (176), which inhibits estrogen receptor-induced activity (178, 180, 229), then this mechanism may also contribute to lower TAG levels, as estrogen has been shown to increase hepatic production of VLDL1-TAG (230).

The sex-specific responses in LDL and HDL measures after the n-3 intervention may also be related to the sexual dimorphism in glucose-insulin homeostasis and associated changes in VLDL metabolism. Higher circulating TRL levels increase the activity of cholesteryl ester transfer protein (CETP) (202, 231–234), which exchanges TAGs from TRLs for cholesteryl esters from LDLs and HDLs (231–234). LPL promotes the exchange of lipids between lipoproteins (218, 235), and the compositional changes make LDL and HDL particles better substrates for hepatic lipase (HL) and to a lesser degree LPL (232–234, 236). HL activity is normally higher in males than females (43, 237, 238) and is elevated in abdominal obesity and hyperglycemic and insulin-resistant states (234, 237–240). Increased hepatic production of TRLs and subsequent CETP-mediated lipid exchange and lipolytic actions of LPL and HL typically result in fewer larger cholesterol-enriched LDLs and HDLs, more numerous smaller TAG-enriched LDLs and HDLs, and smaller mean LDL and HDL particle sizes (141, 202, 232, 233, 236, 241–243). The effects of n-3 PUFAs on these pathways may be modulated by glucose and insulin responses and thus explain some of the sex differences observed in the present study, of which the sexual dimorphism in small, dense LDLs may translate into a lower CVD risk in females compared with males (244–248). Finally, while multiple lines of evidence point to several specific mechanisms possibly explaining the sex-dependent responses we observed after n-3 supplementation, we are not aware of experimental data that could indicate reasons behind the sex differences in VLDL-related measures or Lp(a) after the n-6 intervention.

Strengths and limitations of our crossover trial have been discussed previously (83). Other strengths related specifically to the present work include the supplementation of similar PUFA doses per kg body weight across sexes. Moreover, we used cLMMs to obtain baseline-adjusted effect estimates, and we controlled for cross-level bias in the mixed modeling. Among additional limitations, we were not able to recruit the same number of females and males, and the underrepresentation of females in this study may have limited our ability to detect some clinically significant sex differences. Furthermore, our sample included both pre- and postmenopausal females and we lacked data on the time of last menstruation. However, controlling for menopausal status in the mixed modeling had a negligible impact on effect and precision estimates. It should also be recognized that the present study was exploratory since sex-specific responses were not defined as a primary outcome. Finally, while our results show that sex-dependent responses to PUFA supplementation occur even in people with a relatively high pre-treatment n-3 index and low proportion of LA in RBCMs (83), which is typically observed in Nordic countries (59, 249), the findings may not be generalizable to people with a lower EPA + DHA/LA ratio in blood or with other anthropometric traits.

## 5. Conclusion

Our findings demonstrate sexually dimorphic responses after n-3 (but not n-6) supplementation in circulating markers of glycemic control/insulin sensitivity, which improved in females but worsened in males. These differential changes in glucose-insulin homeostasis may partly be related to the sex differences we observed in several components of the lipoprotein-lipid profile following the n-3 intervention. After n-6 supplementation, Lp(a) decreased significantly in females and increased non-significantly in males. These insights motivate greater attention to individual differences in PUFA-mediated regulation of glucose and lipid metabolism, at least in part dependent on differences in body composition and sex-specific hormonal signaling.

## Data availability statement

The raw data supporting the conclusions of this article will be made available by the authors upon request, without undue reservation.

## Ethics statement

This study involved human participants and was reviewed and approved by The Regional Committee for Medical and Health Research Ethics (2014/2336/REK South-East). The patients/ participants provided their written informed consent to participate in this study.

## Author contributions

JL-B, EG, BB, JS, JN, RB, ER, and ON: conceptualization. JL-B, EG, PB, BB, JS, JN, RB, ER, and ON: methodology. JL-B: software, formal analysis, and writing—original draft preparation. JL-B, EG, PB, JN, ER, GM, SD, and ON: validation. JL-B, EG, PB, BB, ES, and ER: investigation. JN, ER, and ON: resources. JL-B and EG: data curation and visualization. JL-B, EG, PB, BB, ES, JN, GM, SD, and ON: writing—review and editing. ER, GM, SD, and ON: supervision. JL-B, EG, BB, ES, JS, JN, RB, ER, and ON: project administration. ER and ON: funding acquisition. All authors contributed to the article and approved the submitted version.

## Funding

This work was financially supported by the Western Norway Regional Health Authority (Bergen), Trond Mohn Foundation (Bergen), and the University of Bergen.

## Conflict of interest

The authors declare that the research was conducted in the absence of any commercial or financial relationships that could be construed as a potential conflict of interest.

## Publisher’s note

All claims expressed in this article are solely those of the authors and do not necessarily represent those of their affiliated organizations, or those of the publisher, the editors and the reviewers. Any product that may be evaluated in this article, or claim that may be made by its manufacturer, is not guaranteed or endorsed by the publisher.

## Glossary

**Table:** 

AA	Arachidonic acid
apo	Apolipoprotein(s)
AMM	ANCOVA mixed model
B1	The first baseline visit/measurements before any intervention
BMI	Body mass index
CETP	Cholesteryl ester transfer protein
cLMM	Constrained linear mixed-effects model
CVD	Cardiovascular disease
DHA	Docosahexaenoic acid
DMBP	Department of Medical Biochemistry and Pharmacology at Haukeland University Hospital
EPA	Eicosapentaenoic acid
FAs	Fatty acids
FSH	Follicle-stimulating hormone
HDLs	High-density lipoprotein particles
HDL-C	HDL cholesterol
HL	Hepatic lipase
HOMA2-IR	Homeostasis model assessment of insulin resistance index 2
HOMA2-%B	Homeostasis model assessment of beta-cell function index 2
HOMA2-%S	Homeostasis model assessment of insulin sensitivity index 2
IDLs	Intermediate-density lipoprotein particles
INCP	Insulin C-peptide
LA	Linoleic acid
LDLs	Low-density lipoprotein particles
LDL-C	LDL cholesterol
LLA	Lipoprotein-lipid-apolipoprotein
Lp(a)	Lipoprotein (a)
LPL	Lipoprotein lipase
NEFAs	Non-esterified fatty acids
NMR	Nuclear magnetic resonance
non-HDL-C	Non-HDL cholesterol
n-3	Omega-3
n-6	Omega-6
psb	Period-specific baselines
PUFAs	Polyunsaturated fatty acids
QUICKI	Quantitative insulin sensitivity check index
RBCMs	Red blood cell membranes
RCT	Randomized controlled trial
rQUICKI	Revised QUICKI
sab	Subject-averaged baselines
SHBG	Sex hormone–binding globulin
s%	Sympercent
TAGs	Triacylglycerols
TC	Total cholesterol
TRLs	TAG-rich lipoproteins
TRL-C	TRL cholesterol
VLDLs	Very-low-density lipoprotein particles
WC	Waist circumference
wt%	Weight percentage

## Footnotes

^1^http://www.dtu.ox.ac.uk/homacalculator/index.php

^2^https://bevital.no

^3^https://www.r-project.org

^4^https://tidyverse.tidyverse.org
