# Supplementary material for: Sex-specific responses in glucose-insulin homeostasis and lipoprotein-lipid components after high-dose supplementation with marine n-3 PUFAs in abdominal obesity: a randomized double-blind crossover study
Source: Front Nutr. 2023 Jun 19;10:1020678. doi: 10.3389/fnut.2023.1020678 (PMC10315503; doi:10.3389/fnut.2023.1020678)
Supplement: Supplementary file 1 [file Data_Sheet_1.PDF]

Supplementary Material

Table of contents

SUPPLEMENTARY TEXT **2**

MATERIALS AND METHODS 2
STATISTICAL ANALYSES 2
RESULTS 2
COVARIABLE-ADJUSTED EFFECT ESTIMATES 2

SUPPLEMENTARY TABLES **3**

SUPPLEMENTARY TABLE 1 BASELINE CHARACTERISTICS BY SEQUENCE AND PERIOD[1] 3
SUPPLEMENTARY TABLE 2 BASELINE CHARACTERISTICS IN BIOCHEMICAL VARIABLES SHOWING ARITHMETIC MEANS (SDS) BY SEQUENCE AND PERIOD[1] 6
SUPPLEMENTARY TABLE 3 ABSOLUTE SEX-SPECIFIC DIFFERENCES AT PRE-TREATMENT BASELINE IN MEAN DIETARY INTAKES PER DAY[1] 8
SUPPLEMENTARY TABLE 4 ABSOLUTE SEX-SPECIFIC DIFFERENCES AT PRE-TREATMENT BASELINE IN RBCM FATTY ACID LEVELS[1] 9
SUPPLEMENTARY TABLE 5 SEX-SPECIFIC RESPONSES IN ABSOLUTE CHANGES FOR RBCM FATTY ACID LEVELS AFTER SEVEN WK OF SUPPLEMENTATION WITH N-3 OR N-6 PUFAS[1] 10
SUPPLEMENTARY TABLE 6 BETWEEN-SEX DIFFERENCES (FEMALES VS. MALES) IN BASELINE- AND PERIOD-ADJUSTED FOLLOW-UP SCORES AFTER SEVEN WK OF SUPPLEMENTATION WITH N-3 OR N-6 PUFAS (SENSITIVITY ANALYSIS)[1] 12
SUPPLEMENTARY TABLE 7 RELATIVE SEX-SPECIFIC DIFFERENCES AT PRE-TREATMENT BASELINE IN LIPOPROTEIN-LIPID-APOLIPOPROTEIN PROFILES[1] 17
SUPPLEMENTARY TABLE 8 ABSOLUTE SEX-SPECIFIC DIFFERENCES AT PRE-TREATMENT BASELINE IN LIPOPROTEIN-LIPID-APOLIPOPROTEIN PROFILES[1] 18
SUPPLEMENTARY TABLE 9 SEX-SPECIFIC RESPONSES IN RELATIVE CHANGES FOR CIRCULATING LEVELS OF LIPOPROTEIN SUBFRACTIONS, LIPIDS, AND APOLIPOPROTEINS AFTER SEVEN WK OF SUPPLEMENTATION WITH N-3 OR N-6 PUFAS[1] 19
SUPPLEMENTARY TABLE 10 SEX-SPECIFIC RESPONSES IN ABSOLUTE CHANGES FOR CIRCULATING LEVELS OF LIPOPROTEIN SUBFRACTIONS, LIPIDS, AND APOLIPOPROTEINS AFTER SEVEN WK OF SUPPLEMENTATION WITH N-3 OR N-6 PUFAS[1] 22
SUPPLEMENTARY TABLE 11 RELATIVE SEX-SPECIFIC DIFFERENCES AT PRE-TREATMENT BASELINE IN ANTHROPOMETRIC MEASURES, CIRCULATING MARKERS OF GLYCEMIC CONTROL / INSULIN SENSITIVITY AND LIVER FUNCTION, AND HORMONE AND KETONE LEVELS[1] 25
SUPPLEMENTARY TABLE 12 ABSOLUTE SEX-SPECIFIC DIFFERENCES AT PRE-TREATMENT BASELINE IN ANTHROPOMETRIC MEASURES, CIRCULATING MARKERS OF GLYCEMIC CONTROL / INSULIN SENSITIVITY AND LIVER FUNCTION, AND HORMONE LEVELS[1] 27
SUPPLEMENTARY TABLE 13 SEX-SPECIFIC RESPONSES IN RELATIVE CHANGES FOR ANTHROPOMETRIC MEASURES, CIRCULATING MARKERS OF GLYCEMIC CONTROL / INSULIN SENSITIVITY AND LIVER FUNCTION, AND HORMONE AND KETONE LEVELS AFTER SEVEN WK OF SUPPLEMENTATION WITH N-3 OR N-6 PUFAS[1] 28
SUPPLEMENTARY TABLE 14 SEX-SPECIFIC RESPONSES IN ABSOLUTE CHANGES FOR ANTHROPOMETRIC MEASURES, CIRCULATING MARKERS OF GLYCEMIC CONTROL / INSULIN SENSITIVITY AND LIVER FUNCTION, AND HORMONE AND KETONE LEVELS AFTER SEVEN WK OF SUPPLEMENTATION WITH N-3 OR N-6 PUFAS[1] 32

SUPPLEMENTARY FIGURES **36**

SUPPLEMENTARY FIGURE 1 DISTRIBUTION OF DATA POINTS FOR RBCM FATTY ACIDS 36
SUPPLEMENTARY FIGURE 2 DISTRIBUTION OF DATA POINTS FOR LIPOPROTEIN SUBFRACTIONS 37
SUPPLEMENTARY FIGURE 3 DISTRIBUTION OF DATA POINTS FOR LP(A) AND BLOOD LIPIDS 38
SUPPLEMENTARY FIGURE 4 DISTRIBUTION OF DATA POINTS FOR APOLIPOPROTEINS, HOMA2-IR, AND BMI 39
SUPPLEMENTARY FIGURE 5 FLOW DIAGRAM OF THE STUDY PARTICIPANTS 40
SUPPLEMENTARY FIGURE 6 LIPID LEVELS AT BASELINE 41

REFERENCES **42**

SUPPLEMENTARY TEXT

MATERIALS AND METHODS

Statistical analyses

Model validation

The validity of the mixed models was examined in several ways. First, homogeneity of variance was evaluated by visual inspection of plots showing residuals versus fitted values. In case of heterogeneity, we included in the model a variance structure selected through a data-driven approach using as selection criteria the second-order Akaike information criteria (AICc), where the lowest value indicates the best-fitted model. Second, the Shapiro-Wilk test for normality, the D'Agostino test for skewness, and graphical tools (boxplots, quantile- quantile plots, and histograms) were used to assess the distribution of standardized residuals, as well as random effect means (only visual inspection), from the mixed models. Third, cases with extreme values of standardized residuals (cut-off value: z-score >3.29) were further assessed by the Cook's distance (cut-off value: $D > 4/(N - k - 1)$; k = number of explanatory variables, N = number of observations) and standardized dfbetas (cut-off value: >0.3). Potential outliers were excluded one-by-one in sensitivity analyses to explore the influence of these observations on the estimated regression coefficients (change in magnitude/direction), inferential statistics (change in nominal significance), and diagnostic plots (change in patterns). In this model validation procedure, we identified one influential outlier for ALA, two for GGT, and two for estrogen, which was excluded in the final analyses.

In the data-driven approach to select best-fitted variance structures, we used the *aictab* function from the R package *AICcmodavg* v2.3–1. The *CookD* function in the R package *predictmeans* v1.0.6, and the *influence*, *cooks.distance*, and *dfbetas* functions in the R package *influence.ME* v0.9-9 were used to explore potential influential outliers in the mixed models by Cook's distance and DFbetas.

RESULTS

Covariable-adjusted effect estimates

After additionally controlling for age, total energy intake normalized to body weight, alcohol intake normalized to body weight, physical activity level, and serum levels of vitamin D_3, between-sex differences in change scores differed from the primary period- and baseline-adjusted analyses in nominal significance after n-3 supplementation for total HDLs (females vs. males: −11.4%* [−3.65 µmol/L] vs. −4.56% [−1.43 µmol/L], $p = 0.071$; *: significant within-sex change), mean HDL size (+1.41% [+0.17 nm] vs. −0.10% [+0.010 nm], $p = 0.137$), n-3 index (+35.9%* [+3.33 wt%] vs. +50.0%* [+4.33 wt%], $p = 0.007$), EPA (+149%* [+2.37 wt%] vs. +216%* [+2.93 wt%], $p = 0.014$), and DHA (+13.6%* [+0.99 wt%] vs. +20.0%* [+1.38 wt%], $p = 0.022$), and after n-6 supplementation for total VLDLs (+34.4%* [+9.09 nmol/L] vs. +2.72% [+1.09 nmol/L], $p = 0.065$), large VLDLs (+39.5%* [−0.13 nmol/L] vs. −20.8% [−1.74 nmol/L], $p = 0.008$), Lp(a) (−12.4%* [−19.7 mg/L] vs. −0.95% [−3.01 mg/L], $p = 0.069$), and bilirubin (+6.36% [+0.16 µmol/L] vs. −11.8% [−1.33 µmol/L], $p = 0.040$). When we in a separate model included a binary variable for pre-treatment menopausal status among females (0: premenopausal; 1: postmenopausal) and the corresponding age categories among males (0: <55 years; 1: ≥55 years), effect and precision estimates were negligible affected, and the results did not differ from the primary period- and baseline-adjusted analyses in nominal significance for any of the reported variables (data not shown).

SUPPLEMENTARY TABLES

SUPPLEMENTARY TABLE 1 Baseline characteristics by sequence and period[1]

	Sequence AB		Sequence BA	
**Characteristics**	**Period 1 (n = 20)**	**Period 2 (n = 19)**	**Period 1 (n = 19)**	**Period 2 (n = 19)**
**Male (%)**[2]	12 (60)	11 (58)	11 (58)	11 (58)
**Age, years**				
females	55.0 (12.4)		57.6 (5.3)	
males	58.3 (8.4)		51.7 (9.8)	
**BMI, kg/m^2**				
females	28.5 (4.45)	28.7 (4.37)	28.4 (4.85)	28.4 (5.12)
males	30.2 (4.12)	30.6 (4.59)	29.3 (3.61)	29.7 (3.56)
**Waist circumference, cm**				
females	98.8 (11.4)	98.1 (10.4)	101 (11.7)	100.0 (13.1)
males	108 (9.24)	107 (9.67)	106 (8.36)	105 (7.61)
**Visceral fat area, cm^2**				
females	122 (46.1)	137 (54.7)	145 (56.7)	158 (56.2)
males	183 (64.3)	195 (68.8)	182 (60.4)	181 (43.2)
**Energy intake, kcal/d**[3]				
females	1981 (352)	1938 (379)	2002 (506)	1724 (384)
males	2460 (416)	2531 (382)	2327 (661)	2347 (689)
**Carbohydrate, E%**[3]				
females	39.0 (6.93)	39.0 (6.09)	39.3 (6.37)	42.1 (7.00)
males	38.3 (7.49)	40.4 (7.79)	33.9 (6.78)	35.4 (7.91)
**Protein, E%**[3]				
females	15.3 (1.43)	15.9 (1.95)	16.9 (2.39)	18.7 (2.13)
males	17.2 (2.91)	16.6 (2.27)	19.5 (5.22)	19.1 (4.76)
**Fat, E%**[3]				
females	39.2 (3.91)	38.6 (4.18)	36.7 (6.09)	33.1 (5.29)
males	37.7 (5.05)	36.6 (5.36)	41.0 (8.09)	40.9 (6.50)
**SFAs, E%**[3]				
females	13.4 (3.10)	13.8 (2.18)	14.2 (2.15)	12.6 (2.09)
males	14.1 (2.92)	14.1 (3.75)	15.5 (5.26)	15.8 (3.73)
**MUFAs, E%**[3]				
females	11.9 (3.42)	12.5 (2.58)	12.0 (2.71)	11.9 (2.42)
males	12.7 (2.24)	12.6 (2.31)	14.4 (3.31)	14.4 (2.81)
**PUFAs, E%**[3]				
females	5.92 (2.46)	6.04 (1.40)	5.29 (1.71)	4.79 (0.84)
males	6.16 (2.15)	5.36 (1.78)	6.23 (1.48)	5.17 (1.61)
**n-3 PUFAs, E%**[3]				
females	0.88 (0.57)	0.92 (0.43)	0.93 (0.30)	0.82 (0.24)
males	1.38 (0.75)	1.03 (0.36)	1.12 (0.37)	0.97 (0.34)
**n-6 PUFAs, E%**[3]				
females	3.88 (1.96)	3.34 (0.79)	2.54 (0.76)	3.07 (0.64)
males	3.63 (0.86)	3.52 (1.07)	4.13 (0.80)	3.80 (1.21)
**Physical activity score**				
females	13.0 (12.4)	9.75 (10.7)	10.6 (9.61)	12.8 (9.74)
males	7.36 (5.78)	6.00 (5.92)	9.27 (7.55)	9.73 (9.48)
**n-3 index, wt%, RBCMs**				
females	8.60 (7.02, 10.5)	9.97 (8.35, 11.9)	8.92 (8.15, 9.77)	9.04 (7.91, 10.3)
males	8.55 (6.95, 10.5)	9.62 (8.44, 11.0)	7.99 (6.87, 9.29)	7.93 (6.58, 9.57)
**LA, wt%, RBCMs**				
females	8.95 (8.34, 9.60)	8.77 (8.10, 9.50)	8.66 (8.08, 9.29)	8.57 (8.00, 9.18)
males	8.72 (7.92, 9.61)	8.60 (7.63, 9.70)	8.75 (7.96, 9.60)	8.75 (8.02, 9.55)
**Total VLDLs, nmol/L**				
females	33.0 (17.2, 63.4)	33.4 (22.8, 49.0)	33.5 (12.9, 86.8)	38.1 (19.7, 73.5)
males	51.4 (29.1, 90.7)	55.0 (37.6, 80.3)	50.1 (36.2, 69.4)	51.3 (32.7, 80.5)
**Large VLDLs, nmol/L**				
females	0.98 (0.32, 2.98)	1.93 (0.44, 8.47)	3.28 (0.85, 12.6)	2.75 (0.64, 11.9)
males	5.80 (2.82, 11.9)	7.39 (3.87, 14.1)	2.33 (0.77, 7.01)	3.76 (1.36, 10.4)
**Small VLDLs, nmol/L**				
females	12.0 (4.68, 31.0)	12.9 (5.30, 31.2)	22.8 (8.69, 60.1)	15.3 (5.02, 46.6)
males	23.2 (16.1, 33.4)	21.4 (6.92, 66.5)	23.6 (13.8, 40.2)	27.1 (15.3, 47.9)
**Total LDLs, nmol/L**				
females	1151 (963, 1375)	1094 (794, 1506)	1370 (1113, 1687)	1357 (983, 1874)
males	1405 (1093, 1805)	1412 (1105, 1805)	1335 (1054, 1692)	1364 (1070, 1737)

Characteristics	Sequence AB Period 1 (n = 20)	Sequence AB Period 2 (n = 19)	Sequence BA Period 1 (n = 19)	Sequence BA Period 2 (n = 19)
**Large LDLs, nmol/L**				
females	599 (507, 707)	588 (427, 809)	532 (349, 812)	519 (312, 864)
males	420 (266, 662)	354 (202, 621)	463 (334, 642)	499 (386, 644)
**Small LDLs, nmol/L**				
females	201 (76.0, 532)	186 (59.0, 589)	375 (114, 1234)	401 (123, 1304)
males	667 (426, 1044)	787 (548, 1129)	602 (420, 862)	666 (516, 860)
**Total HDLs, µmol/L**				
females	32.6 (29.1, 36.5)	32.6 (27.4, 38.7)	32.0 (27.4, 37.5)	30.7 (26.5, 35.6)
males	28.7 (24.7, 33.3)	29.8 (26.3, 33.8)	28.6 (25.4, 32.1)	28.6 (24.5, 33.4)
**Large HDLs, µmol/L**				
females	10.8 (7.52, 15.5)	11.4 (7.40, 17.7)	8.32 (5.49, 12.6)	8.04 (5.08, 12.7)
males	3.81 (2.36, 6.13)	5.12 (3.67, 7.14)	4.86 (3.16, 7.47)	5.42 (3.44, 8.53)
**Small HDLs, µmol/L**				
females	14.9 (11.7, 19.0)	9.94 (6.13, 16.1)	14.6 (8.89, 23.9)	14.4 (12.0, 17.3)
males	19.3 (16.1, 23.2)	18.0 (14.7, 22.1)	17.8 (16.0, 19.9)	16.9 (15.6, 18.3)
**Lp(a), mg/L**				
females	200 (96.7, 414)	191 (86.9, 422)	73.9 (33.7, 162)	76.7 (33.3, 177)
males	53.5 (27.8, 103)	48.4 (26.7, 87.7)	147 (48.2, 448)	137 (45.7, 412)
**TAGs, mmol/L**				
females	1.04 (0.77, 1.40)	1.01 (0.63, 1.61)	1.35 (0.82, 2.22)	1.20 (0.64, 2.24)
males	1.56 (1.00, 2.42)	1.64 (1.12, 2.41)	1.23 (0.81, 1.86)	1.28 (0.83, 1.96)
**NEFAs, mmol/L**				
females	0.59 (0.34, 1.04)	0.43 (0.26, 0.70)	0.61 (0.41, 0.90)	0.51 (0.30, 0.85)
males	0.54 (0.35, 0.84)	0.42 (0.26, 0.68)	0.33 (0.16, 0.67)	0.48 (0.31, 0.74)
**TC, mmol/L**				
females	5.91 (5.46, 6.40)	6.01 (5.20, 6.94)	6.21 (5.42, 7.11)	6.27 (5.46, 7.20)
males	5.47 (4.65, 6.44)	5.60 (4.65, 6.73)	5.34 (4.45, 6.40)	5.42 (4.58, 6.42)
**LDL-C, mmol/L**				
females	3.79 (3.23, 4.44)	3.82 (3.03, 4.80)	4.17 (3.49, 4.99)	4.36 (3.60, 5.27)
males	3.78 (3.08, 4.64)	3.95 (3.15, 4.96)	3.67 (2.91, 4.63)	3.85 (3.16, 4.69)
**HDL-C, mmol/L**				
females	1.83 (1.38, 2.45)	1.89 (1.34, 2.65)	1.57 (1.13, 2.20)	1.57 (1.18, 2.09)
males	1.16 (0.96, 1.41)	1.18 (0.95, 1.47)	1.23 (1.01, 1.50)	1.22 (0.96, 1.55)
**non-HDL-C, mmol/L**				
females	3.96 (3.27, 4.81)	3.96 (3.05, 5.14)	4.55 (3.81, 5.43)	4.62 (3.76, 5.66)
males	4.26 (3.44, 5.28)	4.35 (3.40, 5.57)	4.06 (3.21, 5.14)	4.15 (3.33, 5.17)
**ApoB, g/L**				
females	1.01 (0.88, 1.16)	1.03 (0.85, 1.24)	1.19 (1.03, 1.38)	1.20 (1.00, 1.43)
males	1.09 (0.91, 1.31)	1.13 (0.92, 1.39)	1.06 (0.86, 1.30)	1.08 (0.90, 1.29)
**ApoA-I, g/L**				
females	1.69 (1.47, 1.95)	1.71 (1.42, 2.07)	1.62 (1.36, 1.92)	1.58 (1.37, 1.82)
males	1.36 (1.23, 1.51)	1.36 (1.22, 1.52)	1.38 (1.21, 1.58)	1.36 (1.17, 1.57)
**ApoA-II, g/L**				
females	0.47 (0.44, 0.50)	0.49 (0.46, 0.52)	0.46 (0.42, 0.50)	0.48 (0.44, 0.52)
males	0.47 (0.43, 0.52)	0.45 (0.40, 0.51)	0.47 (0.42, 0.52)	0.47 (0.40, 0.54)
**ApoC-II, g/L**				
females	0.20 (0.17, 0.25)	0.21 (0.17, 0.26)	0.23 (0.19, 0.28)	0.22 (0.17, 0.29)
males	0.22 (0.17, 0.30)	0.23 (0.17, 0.32)	0.17 (0.12, 0.24)	0.19 (0.14, 0.26)
**ApoC-III, g/L**				
females	0.30 (0.26, 0.35)	0.33 (0.28, 0.39)	0.34 (0.27, 0.43)	0.33 (0.24, 0.44)
males	0.28 (0.20, 0.39)	0.28 (0.20, 0.40)	0.24 (0.18, 0.33)	0.27 (0.19, 0.40)
**ApoE, g/L**				
females	0.083 (0.069, 0.099)	0.093 (0.081, 0.11)	0.091 (0.071, 0.12)	0.093 (0.082, 0.10)
males	0.096 (0.074, 0.12)	0.10 (0.078, 0.13)	0.082 (0.063, 0.11)	0.089 (0.072, 0.11)
**Glucose**				
females	5.15 (4.80, 5.53)	5.53 (5.16, 5.92)	4.87 (4.66, 5.09)	5.21 (4.77, 5.69)
males	5.61 (5.24, 6.02)	5.82 (5.34, 6.34)	5.20 (4.70, 5.76)	5.47 (5.01, 5.98)
**Insulin**				
females	4.90 (3.08, 7.78)	9.04 (6.53, 12.5)	9.83 (5.40, 17.9)	10.7 (5.28, 21.6)
males	11.6 (6.62, 20.4)	15.4 (9.86, 24.0)	9.10 (5.29, 15.7)	8.80 (4.51, 17.2)
**INCP**				
females	0.48 (0.36, 0.65)	0.66 (0.53, 0.83)	0.67 (0.46, 0.98)	0.69 (0.42, 1.13)
males	0.86 (0.58, 1.25)	0.98 (0.72, 1.33)	0.73 (0.54, 0.99)	0.72 (0.50, 1.04)
**HOMA2-IR**				
females	1.07 (0.78, 1.47)	1.50 (1.18, 1.92)	1.46 (0.99, 2.17)	1.53 (0.91, 2.57)
males	1.95 (1.33, 2.87)	2.26 (1.65, 3.10)	1.63 (1.19, 2.23)	1.63 (1.12, 2.37)

Characteristics	Sequence AB Period 1 (n = 20)	Sequence AB Period 2 (n = 19)	Sequence BA Period 1 (n = 19)	Sequence BA Period 2 (n = 19)
**Estrogen**[4]				
**females**	128 (53.4, 305)	119 (44.6, 315)	98.6 (38.5, 252)	93.6 (58.8, 149)
**males**	117 (90.5, 151)	125 (98.9, 157)	116 (93.9, 143)	106 (77.1, 147)
**Testosterone**				
**females**	0.71 (0.45, 1.13)	0.74 (0.45, 1.23)	0.78 (0.38, 1.60)	0.69 (0.34, 1.41)
**males**	14.0 (10.1, 19.4)	14.8 (10.8, 20.4)	13.9 (10.6, 18.1)	13.6 (11.2, 16.5)
**SHBG**				
**females**	54.3 (34.5, 85.5)	49.8 (35.0, 70.9)	54.4 (29.2, 101)	48.5 (27.9, 84.3)
**males**	37.8 (23.7, 60.1)	35.8 (22.3, 57.4)	32.1 (18.2, 56.4)	30.3 (19.0, 48.4)
**Vitamin D_3**				
**females**	76.2 (59.1, 98.3)	66.2 (49.4, 88.8)	73.1 (63.4, 84.4)	61.1 (47.5, 78.5)
**males**	71.7 (57.5, 89.3)	53.0 (41.2, 68.2)	76.5 (58.6, 99.9)	50.9 (37.9, 68.6)

[1] Values are geometric means (1 SD ranges) and arithmetic means (SDs) of fasting blood levels and other measurements, respectively, at baseline in period 1 (before randomization) and period 2 (after the washout phase) of each sequence. Pooled values per stratum of sex at the first baseline visit before any intervention are reported elsewhere (Table 2, Supplementary Tables 3, 4, 7, 8, 11, and 12). Pooled values for all participants have been previously reported (1). Sequence AB and BA received the n-3 and n-6 intervention first, respectively. One participant was lost to follow-up after one month during the first intervention period. The n-3 index is the total sum of EPA (C20:5n-3) and DHA (C22:6n-3) measured in RBCMs (wt%). Abbreviations: Apo, apolipoprotein; E%, energy percentage of total energy intake; HDLs, HDL particles; HDL-C, HDL cholesterol; HOMA2-IR, homeostasis model assessment of insulin resistance index 2 (computer model); INCP insulin C-peptide; LA, linoleic acid (C18:2n6); LDLs, LDL particles; LDL-C, LDL cholesterol; Lp(a), lipoprotein (a); MUFAs; monounsaturated fatty acids; n-3, omega-3 PUFAs; n-6, omega-6 PUFAs; NEFAs, non-esterified fatty acids; non-HDL-C, non-HDL cholesterol; PUFAs; polyunsaturated fatty acids; RBCMs, red blood cell membranes; SFAs; saturated fatty acids; SHBG, sex hormone-binding globulin; TAGs, triacylglycerols; TC, total cholesterol; VLDLs, VLDL particles; wt%, weight percentage of total fatty acids.
[2] Values are numbers (%) of males in each sequence and period.
[3] Values for period 1 and period 2 are from the first and second pre-treatment dietary recording, respectively.
[4] Two outliers were excluded from the analyses of estrogen.

SUPPLEMENTARY TABLE 2 Baseline characteristics in biochemical variables showing arithmetic means (SDs) by sequence and period[1]

	Sequence AB		Sequence BA	
**Characteristics**	**Period 1** (n = 20)	**Period 2** (n = 19)	**Period 1** (n = 19)	**Period 2** (n = 19)
**n-3 index, wt%, RBCMs**				
females	8.76 (1.87)	10.1 (1.84)	8.95 (0.81)	9.11 (1.20)
males	8.72 (1.76)	9.70 (1.21)	8.07 (1.18)	8.06 (1.46)
**LA, wt%, RBCMs**				
females	8.96 (0.64)	8.79 (0.71)	8.68 (0.59)	8.59 (0.60)
males	8.76 (0.85)	8.66 (1.07)	8.78 (0.83)	8.78 (0.76)
**Total VLDLs, nmol/L**				
females	39.3 (22.8)	35.5 (12.9)	45.9 (31.5)	45.1 (26.3)
males	58.4 (28.7)	59.0 (25.4)	52.5 (16.2)	55.8 (22.5)
**Large VLDLs, nmol/L**				
females	1.52 (1.35)	4.36 (5.43)	5.94 (6.13)	5.56 (6.00)
males	7.14 (4.46)	8.93 (5.99)	3.94 (4.39)	5.69 (5.13)
**Small VLDLs, nmol/L**				
females	16.8 (12.7)	16.3 (9.05)	30.5 (19.7)	22.9 (17.4)
males	24.7 (9.64)	29.7 (16.9)	26.7 (13.7)	30.8 (15.5)
**Total LDLs, nmol/L**				
females	1167 (208)	1142 (348)	1396 (289)	1418 (430)
males	1447 (381)	1450 (344)	1369 (321)	1399 (319)
**Large LDLs, nmol/L**				
females	606 (102)	616 (203)	572 (224)	568 (212)
males	451 (143)	394 (152)	487 (170)	514 (135)
**Small LDLs, nmol/L**				
females	285 (217)	306 (264)	584 (443)	626 (477)
males	733 (345)	838 (331)	634 (199)	687 (187)
**Total HDLs, µmol/L**				
females	32.8 (3.89)	33.0 (6.02)	32.4 (4.92)	31.0 (4.53)
males	29.0 (4.65)	30.0 (3.75)	28.7 (3.59)	28.9 (4.48)
**Large HDLs, µmol/L**				
females	11.4 (3.64)	12.4 (5.13)	8.96 (3.70)	8.81 (4.05)
males	4.22 (2.06)	5.39 (1.87)	5.29 (2.31)	5.96 (2.87)
**Small HDLs, µmol/L**				
females	15.3 (3.61)	10.9 (4.31)	15.9 (6.16)	14.6 (2.60)
males	19.6 (3.52)	18.4 (3.67)	17.9 (1.94)	17.0 (1.29)
**Lp(a), mg/L**				
females	249 (169)	245 (168)	95.2 (71.2)	99.5 (66.4)
males	65.1 (43.9)	57.3 (37.0)	244 (234)	224 (209)
**TAGs, mmol/L**				
females	1.08 (0.33)	1.11 (0.55)	1.51 (0.79)	1.42 (0.87)
males	1.70 (0.78)	1.76 (0.70)	1.33 (0.56)	1.40 (0.67)
**NEFAs, mmol/L**				
females	0.67 (0.34)	0.48 (0.21)	0.64 (0.20)	0.56 (0.25)
males	0.59 (0.26)	0.46 (0.20)	0.40 (0.25)	0.52 (0.21)
**TC, mmol/L**				
females	5.92 (0.48)	6.06 (0.87)	6.26 (0.92)	6.33 (0.89)
males	5.54 (0.95)	5.68 (1.04)	5.42 (1.02)	5.49 (0.91)
**LDL-C, mmol/L**				
females	3.83 (0.58)	3.90 (0.83)	4.24 (0.86)	4.42 (0.85)
males	3.86 (0.85)	4.04 (0.94)	3.76 (0.91)	3.92 (0.76)
**HDL-C, mmol/L**				
females	1.90 (0.53)	1.99 (0.69)	1.65 (0.54)	1.62 (0.49)
males	1.18 (0.23)	1.21 (0.26)	1.25 (0.27)	1.25 (0.32)
**non-HDL-C, mmol/L**				
females	4.03 (0.72)	4.08 (0.96)	4.61 (0.86)	4.70 (0.92)
males	4.36 (1.01)	4.47 (1.12)	4.16 (1.00)	4.24 (0.91)
**ApoB, g/L**				
females	1.02 (0.13)	1.04 (0.19)	1.20 (0.18)	1.22 (0.21)
males	1.11 (0.21)	1.15 (0.24)	1.08 (0.22)	1.09 (0.20)
**ApoA-I, g/L**				
females	1.71 (0.24)	1.74 (0.35)	1.64 (0.27)	1.60 (0.23)
males	1.37 (0.14)	1.37 (0.14)	1.40 (0.19)	1.37 (0.22)
**ApoA-II, g/L**				
females	0.47 (0.030)	0.49 (0.034)	0.46 (0.034)	0.48 (0.041)
males	0.48 (0.042)	0.46 (0.049)	0.47 (0.049)	0.47 (0.066)

Characteristics	Sequence AB Period 1 (n = 20)	Sequence AB Period 2 (n = 19)	Sequence BA Period 1 (n = 19)	Sequence BA Period 2 (n = 19)
**ApoC-II, g/L**				
**females**	0.21 (0.037)	0.21 (0.041)	0.24 (0.053)	0.23 (0.055)
**males**	0.23 (0.073)	0.24 (0.071)	0.18 (0.065)	0.20 (0.064)
**ApoC-III g/L**				
**females**	0.30 (0.042)	0.34 (0.058)	0.35 (0.092)	0.34 (0.092)
**males**	0.29 (0.095)	0.30 (0.091)	0.25 (0.074)	0.29 (0.11)
**ApoE, g/L**				
**females**	0.084 (0.015)	0.093 (0.013)	0.094 (0.023)	0.093 (0.011)
**males**	0.099 (0.032)	0.10 (0.031)	0.085 (0.023)	0.090 (0.020)
**Glucose**				
**females**	5.16 (0.38)	5.54 (0.39)	4.88 (0.22)	5.22 (0.47)
**males**	5.62 (0.40)	5.84 (0.51)	5.23 (0.54)	5.49 (0.47)
**Insulin**				
**females**	5.33 (2.14)	9.47 (3.11)	11.5 (7.08)	13.4 (10.4)
**males**	13.2 (6.42)	16.7 (6.76)	10.8 (8.58)	11.0 (9.34)
**INCP**				
**females**	0.50 (0.18)	0.68 (0.18)	0.71 (0.25)	0.76 (0.34)
**males**	0.91 (0.32)	1.02 (0.31)	0.77 (0.30)	0.77 (0.33)
**HOMA2-IR**				
**females**	1.12 (0.42)	1.55 (0.44)	1.56 (0.56)	1.71 (0.81)
**males**	2.08 (0.78)	2.36 (0.74)	1.71 (0.67)	1.74 (0.76)
**Estrogen**[2]				
**females**	179 (159)	180 (175)	159 (211)	102 (47.3)
**males**	120 (30.6)	128 (29.8)	118 (23.1)	112 (38.1)
**Testosterone**				
**females**	0.78 (0.33)	0.82 (0.40)	0.97 (0.69)	0.83 (0.47)
**males**	14.7 (4.87)	15.5 (4.56)	14.3 (3.64)	13.8 (2.98)
**SHBG**				
**females**	59.8 (30.0)	52.8 (20.0)	63.4 (35.0)	54.9 (27.5)
**males**	41.6 (19.5)	39.5 (17.8)	35.6 (13.2)	33.0 (12.6)
**Vitamin D_3**				
**females**	78.3 (18.6)	68.6 (17.9)	73.8 (10.6)	62.8 (16.6)
**males**	73.2 (15.6)	54.4 (12.7)	79.0 (21.8)	53.0 (15.2)

[1] Values are arithmetic means (SDs) of fasting blood levels at baseline in period 1 (before randomization) and period 2 (after the washout phase) of each sequence. Pooled values per stratum of sex at the first baseline visit before any intervention are reported elsewhere (Table 2, Supplementary Tables 3, 4, 7, 8, 11, and 12). Pooled values for all participants have been previously reported (1). Sequence AB and BA received the n-3 and n-6 intervention first, respectively. One participant was lost to follow-up after one month during the first intervention period. The n-3 index is the total sum of EPA (C20:5n-3) and DHA (C22:6n-3) measured in RBCMs (wt%). Abbreviations: Apo, apolipoprotein; HDLs, HDL particles; HDL-C, HDL cholesterol; HOMA2-IR, homeostasis model assessment of insulin resistance index 2 (computer model); INCP, insulin C-peptide; LA, linoleic acid (C18:2n6); LDLs, LDL particles; LDL-C, LDL cholesterol; Lp(a), lipoprotein (a); n-3, omega-3 PUFAs; n-6, omega-6 PUFAs; NEFAs, non-esterified fatty acids; non-HDL-C, non-HDL cholesterol; PUFAs; polyunsaturated fatty acids; RBCMs, red blood cell membranes; SHBG, sex hormone-binding globulin; TAGs, triacylglycerols; TC, total cholesterol; VLDLs, VLDL particles; wt%, weight percentage of total fatty acids.

[2] Two outliers were excluded from the analyses of estrogen.

SUPPLEMENTARY TABLE 3 Absolute sex-specific differences at pre-treatment baseline in mean dietary intakes per day[1]

Variable and treatment	Females[2]	Males[2]	Absolute difference[3]	P-value[4]
**Dietary intake not normalized**				
Energy, kcal	1992 (421)	2394 (543)	−402 (−721, −82.5)	0.019
Carbohydrate, g	198 (64.7)	218 (73.7)	−20.0 (−65.1, 25.2)	0.392
Protein, g	83.9 (20.6)	106 (30.3)	−21.6 (−38.8, −4.43)	0.019
Fat, g	79.6 (18.3)	106 (23.4)	−26.4 (−40.2, −12.6)	0.001
Fiber, g	19.5 (4.27)	19.7 (6.08)	−0.25 (−3.73, 3.23)	0.888
Added sugar, g	27.9 (12.8)	28.8 (29.9)	−0.91 (−16.5, 14.7)	0.909
Alcohol, g	12.5 (11.8)	15.5 (13.4)	−3.00 (−11.2, 5.22)	0.479
SFAs, g	30.3 (8.15)	39.5 (13.4)	−9.16 (−16.6, −1.75)	0.021
MUFAs, g	26.4 (8.01)	36.3 (11.3)	−9.93 (−16.4, −3.44)	0.005
PUFAs, g	12.3 (5.05)	16.7 (6.43)	−4.43 (−8.23, −0.64)	0.028
TRFAs, g	0.63 (0.20)	0.89 (0.51)	−0.26 (−0.52, 0.010)	0.068
n-3 PUFAs, g	1.97 (1.06)	3.31 (1.75)	−1.34 (−2.31, −0.38)	0.010
n-6 PUFAs, g	7.04 (3.69)	10.4 (3.53)	−3.40 (−5.72, −1.08)	0.007
Cholesterol, mg	253 (96.4)	394 (154)	−141 (−227, −55.1)	0.003
**Dietary intake per 100 kg body weight**				
Energy, kcal	2574 (799)	2527 (695)	47.0 (−429, 523)	0.848
Carbohydrate, g	255 (103)	231 (87.7)	24.1 (−36.7, 84.9)	0.442
Protein, g	103 (32.6)	112 (27.7)	−9.13 (−28.3, 10.1)	0.358
Fat, g	110 (38.7)	111 (36.7)	−1.76 (−25.9, 22.4)	0.888
Fiber, g	25.3 (8.39)	20.9 (7.39)	4.38 (−0.66, 9.41)	0.097
Added sugar, g	35.9 (17.7)	30.8 (34.0)	5.06 (−13.2, 23.3)	0.591
Alcohol, g	15.5 (14.5)	16.2 (13.7)	−0.75 (−9.77, 8.27)	0.872
SFAs, g	39.6 (14.8)	41.7 (15.6)	−2.10 (−12.0, 7.75)	0.678
MUFAs, g	34.6 (13.3)	38.4 (14.1)	−3.81 (−12.7, 5.06)	0.405
PUFAs, g	16.1 (8.17)	17.5 (7.04)	−1.39 (−6.24, 3.46)	0.578
TRFAs, g	0.83 (0.35)	0.94 (0.57)	−0.12 (−0.43, 0.20)	0.468
n-3 PUFAs, g	2.62 (1.68)	3.51 (1.90)	−0.90 (−2.06, 0.27)	0.140
n-6 PUFAs, g	9.19 (5.70)	11.0 (4.13)	−1.81 (−4.93, 1.31)	0.263
Cholesterol, mg	330 (136)	418 (173)	−88.6 (−191, 13.7)	0.098
**Dietary intake per 100 kg fat-free mass**				
Energy, kcal	4174 (1087)	3628 (811)	546 (−64.9, 1157)	0.089
Carbohydrate, g	414 (150)	332 (112)	81.3 (−2.83, 166)	0.067
Protein, g	167 (45.8)	160 (32.7)	6.82 (−18.5, 32.1)	0.600
Fat, g	177 (52.3)	161 (46.5)	15.9 (−16.0, 47.8)	0.335
Fiber, g	40.9 (11.2)	30.2 (9.59)	10.7 (4.00, 17.4)	0.004
Added sugar, g	57.8 (25.6)	44.6 (46.0)	13.1 (−12.0, 38.3)	0.313
Alcohol, g	26.1 (24.5)	21.8 (18.9)	4.23 (−9.75, 18.2)	0.557
SFAs, g	63.9 (20.4)	61.0 (21.2)	2.88 (−10.7, 16.5)	0.681
MUFAs, g	55.7 (19.1)	55.1 (17.3)	0.60 (−11.2, 12.4)	0.921
PUFAs, g	26.1 (12.2)	24.8 (9.29)	1.31 (−5.60, 8.23)	0.712
TRFAs, g	1.33 (0.47)	1.38 (0.82)	−0.053 (−0.50, 0.40)	0.819
n-3 PUFAs, g	4.22 (2.51)	4.86 (2.55)	−0.63 (−2.28, 1.02)	0.458
n-6 PUFAs, g	14.9 (8.65)	15.7 (5.25)	−0.84 (−5.34, 3.66)	0.717
Cholesterol, mg	533 (206)	598 (230)	−65.0 (−208, 78.3)	0.380

[1] Dietary intakes per day were analyzed with GLS models adjusted for heterogeneity of variance by using the *gls* function in the R package *nlme* v3.1-157. Abbreviations: MUFAs, monounsaturated fatty acids; n-3, omega-3 PUFAs; n-6, omega-6 PUFAs; PUFAs, polyunsaturated fatty acids; SFAs, saturated fatty acids; TRFAs, trans fatty acids.
[2] Values are arithmetic means (SDs) of dietary intakes at the first baseline measurement before any intervention.
[3] Absolute model-adjusted differences between sexes (females vs. males) at baseline.
[4] P-values for absolute between-sex differences.

SUPPLEMENTARY TABLE 4 Absolute sex-specific differences at pre-treatment baseline in RBCM fatty acid levels[1]

Variable and treatment	Females[2]	Males[2]	Absolute difference[3]	P-value[4]
**Relative contents of RBCM fatty acids**				
**n-6/n-3 ratio**	2.40 (0.44)	2.50 (0.49)	−0.092 (−0.39, 0.21)	0.551
**n-3 index, wt%**	8.86 (1.40)	8.41 (1.51)	0.45 (−0.49, 1.38)	0.354
**ALA, wt%[5]**	0.20 (0.052)	0.20 (0.035)	0.002 (−0.028, 0.032)	0.890
**EPA, wt%**	1.50 (0.54)	1.38 (0.46)	0.12 (−0.19, 0.44)	0.449
**DPA, wt%**	2.97 (0.28)	3.10 (0.28)	−0.13 (−0.31, 0.049)	0.164
**DHA, wt%**	7.36 (0.92)	7.03 (1.12)	0.33 (−0.34, 0.99)	0.340
**LA, wt%**	8.82 (0.61)	8.77 (0.82)	0.053 (−0.42, 0.53)	0.828
**GLA, wt%**	0.047 (0.011)	0.058 (0.017)	−0.011 (−0.020, −0.002)	0.019
**DGLA, wt%**	1.51 (0.20)	1.67 (0.30)	−0.16 (−0.32, −0.004)	0.052
**AA, wt%**	15.4 (1.44)	15.1 (1.49)	0.30 (−0.63, 1.24)	0.530
**Concentrations of RBCM fatty acids**				
**TFAs, µg/mL**	760 (58.7)	743 (43.6)	16.8 (−15.2, 48.9)	0.310
**ALA, µg/mL[5]**	1.48 (0.41)	1.44 (0.26)	0.040 (−0.19, 0.27)	0.734
**EPA, µg/mL**	11.4 (4.12)	10.2 (3.24)	1.21 (−1.11, 3.52)	0.313
**DPA, µg/mL**	22.6 (2.79)	23.0 (2.16)	−0.42 (−1.98, 1.13)	0.595
**DHA, µg/mL**	56.0 (8.82)	52.1 (8.01)	3.85 (−1.47, 9.18)	0.164
**LA, µg/mL**	67.2 (8.24)	65.2 (7.44)	1.95 (−3.01, 6.91)	0.446
**GLA, µg/mL**	0.35 (0.088)	0.44 (0.14)	−0.087 (−0.16, −0.014)	0.026
**DGLA, µg/mL**	11.5 (1.81)	12.5 (2.60)	−0.97 (−2.44, 0.51)	0.207
**AA, µg/mL**	117 (13.2)	113 (13.5)	4.67 (−3.87, 13.2)	0.291

[1] Fasting RBCM fatty acid levels were analyzed with GLS models adjusted for heterogeneity of variance by using the *gls* function in the R package *nlme* v3.1-157. Abbreviations: AA, arachidonic acid; ALA, α-linolenic acid; DGLA, dihomo-γ-linolenic acid; DHA, docosahexaenoic acid; DPA, docosapentaenoic acid; EPA, eicosapentaenoic acid; GLA, γ-linolenic acid; GLS, generalized least squares; LA, linoleic acid; n-3, omega-3 PUFAs; n-6, omega-6 PUFAs; PUFAs, polyunsaturated fatty acids; RBCMs, red blood cell membranes; TFAs, total fatty acids; wt%, weight percentage of total fatty acids.
[2] Values are arithmetic means (SDs) of fasting RBCM fatty acid levels at the first baseline measurement before any intervention.
[3] Absolute model-adjusted differences between sexes (females vs. males) at baseline.
[4] P-values for absolute between-sex differences.
[5] One outlier was excluded from the analyses of ALA.

frontiers

SUPPLEMENTARY TABLE 5 Sex-specific responses in absolute changes for RBCM fatty acid levels after seven wk of supplementation with n-3 or n-6 PUFAs[1]

Variable and treatment	Baseline[2]	Follow-up[2]	Absolute change[3]	Time[4]	wTXbSEX[5]	bTXwSEX[6]
**Relative contents of RBCM fatty acids**						
**n-6/n-3 ratio**						
**n-3 : females**	2.40 (0.45)	1.50 (0.27)	−0.73 (−0.85, −0.61)	<0.001	0.116	<0.001
**n-3 : males**	2.54 (0.55)	1.46 (0.27)	−0.86 (−0.97, −0.75)	<0.001		<0.001
**n-6 : females**	2.15 (0.37)	2.46 (0.36)	+0.16 (0.089, 0.24)	<0.001	0.116	
**n-6 : males**	2.31 (0.49)	2.70 (0.45)	+0.24 (0.17, 0.30)	<0.001		
**n-3 index, wt%**						
**n-3 : females**	8.93 (1.53)	12.6 (1.48)	+3.18 (2.59, 3.76)	<0.001	0.072	<0.001
**n-3 : males**	8.40 (1.62)	12.7 (1.73)	+3.88 (3.38, 4.38)	<0.001		<0.001
**n-6 : females**	9.53 (1.50)	8.86 (1.27)	−0.31 (−0.58, −0.040)	0.025	0.651	
**n-6 : males**	8.88 (1.43)	8.10 (1.14)	−0.39 (−0.63, −0.16)	0.001		
**ALA, wt%[7]**						
**n-3 : females**	0.18 (0.049)	0.16 (0.031)	−0.034 (−0.057, −0.012)	0.003	0.846	0.520
**n-3 : males**	0.18 (0.038)	0.15 (0.035)	−0.032 (−0.050, −0.013)	0.001		0.086
**n-6 : females**	0.20 (0.048)	0.15 (0.032)	−0.040 (−0.055, −0.025)	<0.001	0.703	
**n-6 : males**	0.19 (0.044)	0.14 (0.018)	−0.044 (−0.056, −0.031)	<0.001		
**EPA, wt%**						
**n-3 : females**	1.54 (0.68)	4.15 (0.96)	+2.22 (1.77, 2.67)	<0.001	0.164	<0.001
**n-3 : males**	1.36 (0.51)	4.32 (0.93)	+2.64 (2.25, 3.02)	<0.001		<0.001
**n-6 : females**	1.92 (0.72)	1.42 (0.52)	−0.26 (−0.39, −0.12)	<0.001	0.751	
**n-6 : males**	1.64 (0.55)	1.16 (0.30)	−0.23 (−0.34, −0.11)	<0.001		
**DPA, wt%**						
**n-3 : females**	2.94 (0.29)	3.75 (0.28)	+0.53 (0.43, 0.63)	<0.001	0.036	<0.001
**n-3 : males**	3.06 (0.25)	4.00 (0.25)	+0.66 (0.58, 0.75)	<0.001		<0.001
**n-6 : females**	3.25 (0.40)	2.99 (0.26)	−0.11 (−0.16, −0.046)	0.001	0.388	
**n-6 : males**	3.37 (0.39)	3.07 (0.26)	−0.14 (−0.19, −0.088)	<0.001		
**DHA, wt%**						
**n-3 : females**	7.39 (0.96)	8.45 (0.64)	+0.91 (0.62, 1.21)	<0.001	0.160	<0.001
**n-3 : males**	7.05 (1.16)	8.36 (0.92)	+1.19 (0.94, 1.44)	<0.001		<0.001
**n-6 : females**	7.62 (0.86)	7.44 (0.83)	−0.062 (−0.22, 0.096)	0.438	0.316	
**n-6 : males**	7.25 (0.99)	6.93 (0.93)	−0.17 (−0.30, −0.032)	0.016		
**LA, wt%**						
**n-3 : females**	8.78 (0.63)	7.22 (0.67)	−1.60 (−1.90, −1.29)	<0.001	0.964	<0.001
**n-3 : males**	8.77 (0.79)	7.27 (0.91)	−1.59 (−1.85, −1.33)	<0.001		<0.001
**n-6 : females**	8.74 (0.63)	9.77 (0.50)	+0.99 (0.68, 1.29)	<0.001	0.346	
**n-6 : males**	8.72 (0.94)	10.1 (1.00)	+1.18 (0.92, 1.44)	<0.001		
**GLA, wt%**						
**n-3 : females**	0.048 (0.013)	0.026 (0.005)	−0.021 (−0.028, −0.014)	<0.001	0.827	<0.001
**n-3 : males**	0.056 (0.019)	0.033 (0.014)	−0.022 (−0.029, −0.015)	<0.001		<0.001
**n-6 : females**	0.046 (0.013)	0.047 (0.012)	+0.000 (−0.008, 0.007)	0.933	0.707	
**n-6 : males**	0.055 (0.014)	0.057 (0.015)	+0.002 (−0.005, 0.008)	0.644		
**DGLA, wt%**						
**n-3 : females**	1.51 (0.19)	1.14 (0.18)	−0.37 (−0.43, −0.30)	<0.001	0.046	<0.001
**n-3 : males**	1.68 (0.30)	1.21 (0.24)	−0.47 (−0.54, −0.39)	<0.001		<0.001
**n-6 : females**	1.49 (0.20)	1.50 (0.18)	−0.003 (−0.069, 0.064)	0.933	0.454	
**n-6 : males**	1.65 (0.29)	1.71 (0.29)	+0.034 (−0.037, 0.11)	0.345		
**AA, wt%**						
**n-3 : females**	15.5 (1.47)	13.9 (1.24)	−1.19 (−1.55, −0.83)	<0.001	0.025	<0.001
**n-3 : males**	15.2 (1.55)	13.3 (1.14)	−1.74 (−2.04, −1.43)	<0.001		<0.001
**n-6 : females**	14.9 (1.35)	15.2 (1.27)	+0.13 (−0.18, 0.45)	0.410	0.880	
**n-6 : males**	14.8 (1.57)	15.2 (1.34)	+0.16 (−0.11, 0.43)	0.231		
**Concentrations of RBCM fatty acids**						
**TFAs, µg/mL**						
**n-3 : females**	773 (71.1)	784 (77.6)	+6.04 (−22.3, 34.4)	0.674	0.483	0.699
**n-3 : males**	747 (42.4)	744 (42.5)	−6.46 (−27.3, 14.4)	0.541		0.889
**n-6 : females**	782 (84.7)	789 (84.7)	+11.2 (−17.2, 39.5)	0.436	0.294	
**n-6 : males**	753 (53.9)	742 (43.9)	−7.53 (−28.4, 13.3)	0.476		
**ALA, µg/mL**						
**n-3 : females**	1.39 (0.41)	1.21 (0.35)	−0.24 (−0.41, −0.059)	0.009	0.992	0.456
**n-3 : males**	1.35 (0.28)	1.13 (0.23)	−0.24 (−0.38, −0.092)	0.002		0.078
**n-6 : females**	1.51 (0.38)	1.15 (0.23)	−0.29 (−0.41, −0.16)	<0.001	0.523	
**n-6 : males**	1.42 (0.38)	1.04 (0.13)	−0.34 (−0.44, −0.24)	<0.001		

Variable and treatment	Baseline[2]	Follow-up[2]	Absolute change[3]	Time[4]	wTXbSEX[5]	bTXwSEX[6]
**EPA, µg/mL**						
**n-3 : females**	11.7 (4.66)	32.7 (8.93)	+18.3 (14.2, 22.4)	<0.001	0.600	<0.001
**n-3 : males**	10.0 (3.68)	32.1 (7.59)	+19.7 (16.3, 23.2)	<0.001		<0.001
**n-6 : females**	15.1 (6.34)	11.2 (4.31)	−1.66 (−2.65, −0.66)	0.001	0.815	
**n-6 : males**	12.3 (4.26)	8.64 (2.33)	−1.81 (−2.66, −0.95)	<0.001		
**DPA, µg/mL**						
**n-3 : females**	22.7 (2.60)	29.5 (4.12)	+5.32 (3.85, 6.79)	<0.001	0.735	<0.001
**n-3 : males**	22.8 (1.49)	29.7 (2.14)	+5.64 (4.40, 6.89)	<0.001		<0.001
**n-6 : females**	25.5 (4.71)	23.6 (2.84)	−0.44 (−1.45, 0.58)	0.398	0.253	
**n-6 : males**	25.3 (3.20)	22.8 (1.98)	−1.19 (−2.05, −0.33)	0.007		
**DHA, µg/mL**						
**n-3 : females**	57.0 (8.21)	66.3 (9.22)	+7.97 (4.53, 11.4)	<0.001	0.640	<0.001
**n-3 : males**	52.5 (8.04)	62.2 (8.08)	+8.97 (6.51, 11.4)	<0.001		<0.001
**n-6 : females**	59.7 (10.9)	58.9 (10.9)	+0.49 (−2.95, 3.93)	0.779	0.320	
**n-6 : males**	54.5 (8.08)	51.5 (7.77)	−1.65 (−4.11, 0.82)	0.188		
**LA, µg/mL**						
**n-3 : females**	68.1 (9.84)	56.5 (6.90)	−11.7 (−15.2, −8.15)	<0.001	0.977	<0.001
**n-3 : males**	65.5 (7.50)	54.0 (7.23)	−11.7 (−14.7, −8.74)	<0.001		<0.001
**n-6 : females**	68.3 (8.66)	77.0 (8.58)	+8.81 (5.29, 12.3)	<0.001	0.959	
**n-6 : males**	65.8 (9.69)	74.7 (9.07)	+8.93 (5.94, 11.9)	<0.001		
**GLA, µg/mL**						
**n-3 : females**	0.36 (0.13)	0.21 (0.056)	−0.16 (−0.21, −0.099)	<0.001	0.654	<0.001
**n-3 : males**	0.42 (0.15)	0.25 (0.10)	−0.17 (−0.22, −0.12)	<0.001		<0.001
**n-6 : females**	0.36 (0.11)	0.38 (0.10)	+0.012 (−0.045, 0.068)	0.678	0.830	
**n-6 : males**	0.42 (0.13)	0.42 (0.12)	+0.004 (−0.048, 0.055)	0.890		
**DGLA, µg/mL**						
**n-3 : females**	11.7 (2.03)	8.87 (1.50)	−2.70 (−3.50, −1.91)	<0.001	0.146	<0.001
**n-3 : males**	12.6 (2.53)	8.99 (1.96)	−3.48 (−4.15, −2.80)	<0.001		<0.001
**n-6 : females**	11.7 (2.06)	11.9 (2.19)	+0.17 (−0.35, 0.70)	0.517	0.993	
**n-6 : males**	12.5 (2.68)	12.7 (2.53)	+0.17 (−0.28, 0.62)	0.456		
**AA, µg/mL**						
**n-3 : females**	119 (14.0)	109 (12.6)	−8.91 (−13.3, −4.57)	<0.001	0.062	<0.001
**n-3 : males**	114 (14.8)	98.9 (10.1)	−14.4 (−18.2, −10.6)	<0.001		<0.001
**n-6 : females**	116 (14.0)	120 (15.7)	+2.42 (−1.92, 6.76)	0.272	0.328	
**n-6 : males**	112 (13.9)	113 (11.3)	−0.44 (−4.24, 3.36)	0.818		

[1] Fasting RBCM fatty acid levels were analyzed with cLMMs adjusted for the main effects of period and subject-averaged baselines. Abbreviations: AA, arachidonic acid; ALA, α-linolenic acid; bTXwSEX, between-treatment within-sex; DGLA, dihomo-γ-linolenic acid; DHA, docosahexaenoic acid; DPA, docosapentaenoic acid; EPA, eicosapentaenoic acid; GLA, γ-linolenic acid; LA, linoleic acid; cLMM, constrained linear mixed-effects model; n-3, omega-3 PUFAs; n-6, omega-6 PUFAs; PUFAs, polyunsaturated fatty acids; RBCM, red blood cell membrane; TFAs, total fatty acids; wTXbSEX, within-treatment between-sex; wt%, weight percentage of total fatty acids.
[2] Values are arithmetic means (SDs) of fasting RBCM levels at baseline and follow-up within treatments and sexes.
[3] Absolute model-adjusted mean change scores (95% CIs) from baseline to follow-up.
[4] P-values for absolute changes from baseline to follow-up within treatments and sexes (time effects).
[5] P-values for absolute changes from baseline to follow-up within treatments and between sexes (sex differences in time effects). The first and second values refer to between-sex differences after the n-3 and n-6 interventions, respectively.
[6] P-values for absolute changes from baseline to follow-up between treatments and within sexes (group differences in time effects within each stratum of sex). The first and second values refer to between-treatment differences in females and males, respectively.
[7] One influential outlier was excluded from the final analyses of ALA.

SUPPLEMENTARY TABLE 6 Between-sex differences (females vs. males) in baseline- and period-adjusted follow-up scores after seven wk of supplementation with n-3 or n-6 PUFAs (sensitivity analysis)[1]

Variable	Absolute (n = 38)[2]	Relative (n = 38)[3]	P-value[4]	bTXbSEX[5]	P-value[5]
**Lipoprotein subfractions, plasma**					
**Total VLDLs, nmol/L**					
n-3	−0.12 (−14.1, 13.8)	−11.3 (−48.1, 51.7)	0.654	−15.3 (−48.4, 39.0)	0.500
n-6	2.06 (−8.19, 12.3)	4.76 (−15.3, 29.5)	0.659		
**Large VLDLs, nmol/L**					
n-3	0.54 (−1.04, 2.13)	−1.93 (−48.7, 87.5)	0.952	−6.40 (−55.0, 94.6)	0.855
n-6	−0.15 (−1.74, 1.43)	4.78 (−26.1, 48.5)	0.788		
**Medium VLDLs, nmol/L**					
n-3	−4.52 (−12.9, 3.88)	−13.7 (−51.2, 52.6)	0.602	1.84 (−46.4, 93.7)	0.954
n-6	−1.88 (−8.13, 4.37)	−15.3 (−40.7, 21.0)	0.351		
**Small VLDLs, nmol/L**					
n-3	2.31 (−7.89, 12.5)	−2.35 (−52.4, 100)	0.947	−27.0 (−65.9, 56.2)	0.403
n-6	3.80 (−6.17, 13.8)	33.8 (−7.21, 93.0)	0.115		
**Total LDLs, nmol/L**					
n-3	−123 (−233, −13.9)	−6.54 (−13.4, 0.82)	0.079	−3.33 (−13.8, 8.38)	0.552
n-6	−34.9 (−144, 74.5)	−3.33 (−10.4, 4.30)	0.372		
**IDLs, nmol/L**					
n-3	−45.4 (−121, 30.3)	−26.5 (−69.3, 76.1)	0.480	−17.1 (−70.7, 135)	0.716
n-6	−32.9 (−109, 42.8)	−11.3 (−48.8, 53.7)	0.661		
**Large LDLs, nmol/L**					
n-3	109 (1.91, 216)	16.6 (−5.98, 44.5)	0.157	9.44 (−16.1, 42.7)	0.493
n-6	45.4 (−66.6, 157)	6.50 (−14.4, 32.5)	0.561		
**Small LDLs, nmol/L**					
n-3	−191 (−319, −63.1)	−42.9 (−61.0, −16.5)	0.005	−21.2 (−52.3, 30.1)	0.340
n-6	−38.8 (−167, 89.0)	−27.5 (−50.5, 6.05)	0.095		
**Total HDLs, µmol/L**					
n-3	−1.58 (−3.48, 0.32)	−5.66 (−11.6, 0.72)	0.079	−6.98 (−12.0, −1.67)	0.012
n-6	0.67 (−1.24, 2.58)	1.42 (−5.06, 8.35)	0.666		
**Large HDLs, µmol/L**					
n-3	0.36 (−0.90, 1.62)	1.61 (−12.1, 17.4)	0.824	−3.18 (−20.1, 17.3)	0.734
n-6	0.45 (−0.50, 1.40)	4.95 (−10.6, 23.1)	0.543		
**Medium HDLs, µmol/L**					
n-3	−2.48 (−4.37, −0.59)	−39.2 (−64.7, 4.70)	0.072	−46.7 (−69.1, −8.01)	0.025
n-6	0.11 (−1.78, 2.00)	14.1 (−23.7, 70.7)	0.510		
**Small HDLs, µmol/L**					
n-3	−1.15 (−3.17, 0.87)	0.20 (−11.2, 13.1)	0.974	0.065 (−11.5, 13.2)	0.991
n-6	−0.56 (−2.59, 1.47)	0.13 (−12.5, 14.6)	0.985		
**VLDL size, nm**					
n-3	1.68 (−3.89, 7.24)	3.45 (−7.58, 15.8)	0.545	4.38 (−5.14, 14.8)	0.369
n-6	−0.55 (−3.77, 2.67)	−0.89 (−7.17, 5.81)	0.783		
**LDL size, nm**					
n-3	0.18 (−0.007, 0.36)	0.84 (−0.020, 1.71)	0.055	0.52 (−0.80, 1.86)	0.430
n-6	0.069 (−0.21, 0.35)	0.32 (−0.99, 1.65)	0.627		
**HDL size, nm**					
n-3	0.24 (0.065, 0.42)	2.50 (0.57, 4.47)	0.012	1.18 (−1.06, 3.46)	0.295
n-6	0.11 (−0.066, 0.29)	1.31 (−0.60, 3.26)	0.173		
**Lp(a) and blood lipids, serum**					
**Lp(a), mg/L**					
n-3	−4.17 (−20.0, 11.7)	−2.03 (−13.2, 10.6)	0.734	10.9 (−5.48, 30.1)	0.197
n-6	−18.8 (−34.7, −2.90)	−11.6 (−21.8, −0.23)	0.046		
**TAGs, mmol/L**					
n-3	−0.019 (−0.18, 0.14)	−2.60 (−15.7, 12.6)	0.714	−5.29 (−21.0, 13.5)	0.547
n-6	−0.003 (−0.24, 0.23)	2.84 (−11.0, 18.9)	0.698		
**NEFAs, mmol/L**					
n-3	0.17 (0.025, 0.32)	51.3 (10.1, 108)	0.012	20.8 (−16.4, 74.4)	0.304
n-6	0.14 (−0.011, 0.29)	25.3 (−9.02, 72.5)	0.162		
**PLs, mmol/L**					
n-3	0.060 (−0.10, 0.22)	2.06 (−3.56, 7.99)	0.470	−0.93 (−7.89, 6.55)	0.795
n-6	0.097 (−0.064, 0.26)	3.02 (−2.65, 9.01)	0.293		
**TC, mmol/L**					
n-3	0.17 (−0.17, 0.50)	2.75 (−3.13, 8.98)	0.356	2.30 (−4.99, 10.2)	0.536
n-6	0.032 (−0.30, 0.37)	0.44 (−5.31, 6.53)	0.881		

Variable	Absolute (n = 38)[2]	Relative (n = 38)[3]	P-value[4]	bTXbSEX[5]	P-value[5]
**FC, mmol/L**					
n-3	0.049 (−0.048, 0.15)	3.17 (−2.86, 9.58)	0.300	2.53 (−5.06, 10.7)	0.514
n-6	0.011 (−0.086, 0.11)	0.63 (−5.25, 6.88)	0.834		
**LDL-C, mmol/L**					
n-3	0.085 (−0.20, 0.37)	2.50 (−4.62, 10.2)	0.491	5.01 (−4.56, 15.6)	0.306
n-6	−0.095 (−0.38, 0.19)	−2.39 (−9.18, 4.90)	0.500		
**HDL-C, mmol/L**					
n-3	0.032 (−0.088, 0.15)	1.97 (−6.04, 10.7)	0.631	−3.35 (−10.8, 4.77)	0.396
n-6	0.083 (−0.038, 0.20)	5.51 (−2.78, 14.5)	0.192		
**Non-HDL-C, mmol/L**					
n-3	0.066 (−0.22, 0.35)	2.16 (−4.44, 9.20)	0.521	5.46 (−3.29, 15.0)	0.221
n-6	−0.12 (−0.41, 0.16)	−3.13 (−9.39, 3.55)	0.339		
**TRL-C, mmol/L**					
n-3	−0.039 (−0.11, 0.032)	−5.43 (−26.1, 21.0)	0.649	11.8 (−18.0, 52.4)	0.469
n-6	−0.047 (−0.12, 0.024)	−15.4 (−33.9, 8.27)	0.177		
**TAGs/HDL-C ratio**					
n-3	−0.070 (−0.20, 0.058)	−5.00 (−19.6, 12.3)	0.537	−1.75 (−17.3, 16.7)	0.836
n-6	−0.18 (−0.36, −0.006)	−3.31 (−18.2, 14.3)	0.686		
**TC/HDL-C ratio**					
n-3	−0.019 (−0.27, 0.24)	−1.30 (−7.84, 5.69)	0.699	6.26 (−1.57, 14.7)	0.116
n-6	−0.21 (−0.46, 0.046)	−7.12 (−13.3, −0.54)	0.035		
**Apolipoproteins, serum**					
**ApoB, g/L**					
n-3	0.007 (−0.057, 0.072)	1.33 (−4.30, 7.29)	0.642	2.25 (−5.43, 10.6)	0.567
n-6	−0.012 (−0.076, 0.053)	−0.90 (−6.40, 4.92)	0.750		
**ApoA-I, g/L**					
n-3	−0.011 (−0.088, 0.066)	−1.04 (−6.25, 4.46)	0.697	−0.85 (−6.21, 4.82)	0.757
n-6	0.004 (−0.076, 0.083)	−0.19 (−5.53, 5.44)	0.943		
**ApoA-II, g/L**					
n-3	−0.017 (−0.043, 0.009)	−4.60 (−11.3, 2.58)	0.196	−3.58 (−10.8, 4.25)	0.350
n-6	−0.003 (−0.030, 0.023)	−1.06 (−6.02, 4.16)	0.676		
**ApoC-II, g/L**					
n-3	0.007 (−0.014, 0.028)	2.21 (−7.64, 13.1)	0.665	0.49 (−13.3, 16.4)	0.946
n-6	0.005 (−0.016, 0.026)	1.70 (−8.10, 12.6)	0.737		
**ApoC-III, g/L**					
n-3	0.010 (−0.026, 0.046)	2.84 (−9.45, 16.8)	0.658	−3.77 (−19.6, 15.2)	0.667
n-6	0.021 (−0.015, 0.058)	6.86 (−5.93, 21.4)	0.298		
**ApoE, g/L**					
n-3	0.002 (−0.007, 0.010)	1.28 (−7.36, 10.7)	0.774	1.60 (−8.10, 12.3)	0.750
n-6	0.001 (−0.008, 0.009)	−0.31 (−8.81, 8.98)	0.944		
**ApoB/ApoA-I ratio**					
n-3	0.023 (−0.023, 0.070)	2.74 (−3.86, 9.79)	0.414	6.57 (−1.77, 15.6)	0.122
n-6	0.003 (−0.042, 0.047)	−3.59 (−9.79, 3.03)	0.271		
**ApoC-II/ApoC-III ratio**					
n-3	0.013 (−0.034, 0.060)	2.19 (−4.38, 9.22)	0.512	1.20 (−7.85, 11.1)	0.798
n-6	0.007 (−0.040, 0.054)	0.99 (−5.52, 7.95)	0.766		
**Fatty acids, RBCMs**					
**n-6/n-3 ratio**					
n-3	0.12 (0.006, 0.23)	7.83 (0.63, 15.5)	0.033	11.6 (3.92, 19.9)	0.004
n-6	−0.12 (−0.23, −0.006)	−3.39 (−6.80, 0.14)	0.059		
**n-3 index**					
n-3	−0.62 (−1.31, 0.085)	−5.29 (−10.5, 0.17)	0.057	−8.01 (−13.2, −2.50)	0.006
n-6	0.20 (−0.081, 0.49)	2.95 (−0.50, 6.53)	0.092		
**ALA, wt%**					
n-3	−0.001 (−0.024, 0.022)	0.72 (−11.4, 14.5)	0.911	−1.86 (−15.0, 13.3)	0.791
n-6	0.006 (−0.006, 0.017)	2.63 (−6.11, 12.2)	0.557		
**EPA, wt%**					
n-3	−0.36 (−0.95, 0.23)	−11.6 (−21.0, −1.11)	0.032	−17.4 (−28.7, −4.32)	0.012
n-6	0.059 (−0.064, 0.18)	7.00 (−4.60, 20.0)	0.239		
**DPA, wt%**					
n-3	−0.17 (−0.30, −0.042)	−4.07 (−6.92, −1.14)	0.008	−3.84 (−7.38, −0.16)	0.041
n-6	−0.007 (−0.095, 0.081)	−0.24 (−3.19, 2.80)	0.872		
**DHA, wt%**					
n-3	−0.26 (−0.66, 0.13)	−2.49 (−5.86, 0.99)	0.153	−5.62 (−9.70, −1.35)	0.012
n-6	0.15 (−0.056, 0.35)	3.31 (−0.28, 7.03)	0.070		

Variable	Absolute (n = 38)[2]	Relative (n = 38)[3]	P-value[4]	bTXbSEX[5]	P-value[5]
**LA, wt%**					
**n-3**	−0.028 (−0.38, 0.32)	−0.65 (−5.65, 4.62)	0.800	2.06 (−3.60, 8.05)	0.473
**n-6**	−0.23 (−0.58, 0.13)	−2.65 (−6.30, 1.15)	0.163		
**GLA, wt%**					
**n-3**	−0.005 (−0.011, 0.002)	−8.35 (−23.6, 9.94)	0.337	0.36 (−17.1, 21.5)	0.970
**n-6**	−0.008 (−0.016, 0.001)	−8.68 (−20.4, 4.83)	0.190		
**DGLA, wt%**					
**n-3**	0.063 (−0.020, 0.15)	3.72 (−5.11, 13.4)	0.410	7.16 (−1.01, 16.0)	0.085
**n-6**	−0.075 (−0.16, 0.009)	−3.21 (−7.07, 0.80)	0.111		
**AA, wt%**					
**n-3**	0.56 (0.22, 0.90)	4.12 (1.21, 7.11)	0.007	4.29 (1.67, 6.97)	0.002
**n-6**	−0.020 (−0.36, 0.32)	−0.16 (−2.08, 1.79)	0.866		
**Glycemic control / insulin sensitivity, serum**					
**Glucose, mmol/L**					
**n-3**	−0.47 (−0.74, −0.20)	−8.12 (−12.3, −3.72)	0.001	−5.43 (−9.99, −0.65)	0.028
**n-6**	−0.15 (−0.42, 0.12)	−2.84 (−7.28, 1.80)	0.219		
**HbA1c, %**					
**n-3**	0.034 (−0.035, 0.10)	0.63 (−0.64, 1.92)	0.320	0.44 (−1.40, 2.31)	0.635
**n-6**	0.012 (−0.064, 0.088)	0.20 (−1.18, 1.59)	0.774		
**Insulin, mU/L**					
**n-3**	−4.82 (−7.17, −2.48)	−41.6 (−52.5, −28.2)	<0.001	−30.5 (−46.7, −9.41)	0.009
**n-6**	−1.56 (−3.90, 0.78)	−16.0 (−31.6, 3.28)	0.096		
**INCP, nmol/L**					
**n-3**	−0.20 (−0.30, −0.096)	−25.9 (−35.1, −15.3)	<0.001	−18.9 (−30.7, −5.07)	0.011
**n-6**	−0.038 (−0.14, 0.062)	−8.55 (−19.9, 4.43)	0.180		
**HOMA2-IR**					
**n-3**	−0.48 (−0.73, −0.24)	−27.8 (−37.2, −16.9)	<0.001	−20.2 (−32.1, −6.18)	0.008
**n-6**	−0.096 (−0.34, 0.15)	−9.50 (−21.3, 4.08)	0.156		
**HOMA2-%S**					
**n-3**	25.0 (14.9, 35.1)	38.3 (20.6, 58.6)	<0.001	25.6 (6.87, 47.5)	0.007
**n-6**	9.43 (−0.68, 19.5)	10.2 (−3.94, 26.4)	0.160		
**HOMA2-%B**					
**n-3**	−8.25 (−19.8, 3.28)	−6.87 (−15.7, 2.85)	0.154	−4.37 (−15.1, 7.75)	0.452
**n-6**	−2.37 (−13.9, 9.15)	−2.61 (−11.8, 7.55)	0.592		
**QUICKI**					
**n-3**	0.014 (0.009, 0.019)	9.65 (6.04, 13.4)	<0.001	6.68 (2.12, 11.5)	0.005
**n-6**	0.004 (−0.001, 0.009)	2.78 (−0.60, 6.29)	0.105		
**rQUICKI**					
**n-3**	0.007 (−0.006, 0.020)	4.16 (−2.96, 11.8)	0.250	3.96 (−4.11, 12.7)	0.336
**n-6**	0.000 (−0.013, 0.013)	0.20 (−6.62, 7.51)	0.956		
**LP-IR**					
**n-3**	−1.52 (−8.52, 5.49)	−13.2 (−34.0, 14.1)	0.300	−0.26 (−30.6, 43.3)	0.988
**n-6**	−7.38 (−14.9, 0.11)	−13.0 (−33.2, 13.2)	0.291		
**Anthropometrics**					
**Body weight, kg**					
**n-3**	−1.14 (−2.01, −0.26)	−1.28 (−2.25, −0.29)	0.013	−1.14 (−2.52, 0.25)	0.105
**n-6**	−0.21 (−1.09, 0.66)	−0.14 (−1.14, 0.87)	0.782		
**BMI, kg/m^2**					
**n-3**	−0.31 (−0.58, −0.036)	−1.02 (−1.94, −0.093)	0.032	−1.17 (−2.54, 0.22)	0.095
**n-6**	0.030 (−0.24, 0.30)	0.15 (−0.78, 1.10)	0.740		
**Body fat mass, kg**					
**n-3**	−0.90 (−1.74, −0.056)	−3.68 (−6.38, −0.91)	0.011	−3.12 (−7.07, 1.01)	0.132
**n-6**	−0.10 (−0.94, 0.74)	−0.58 (−3.39, 2.30)	0.680		
**Body fat, %**					
**n-3**	−0.64 (−1.50, 0.23)	−2.02 (−4.53, 0.55)	0.119	−2.09 (−5.32, 1.24)	0.208
**n-6**	−0.026 (−0.94, 0.89)	0.074 (−2.65, 2.87)	0.957		
**Waist circumference, cm**					
**n-3**	−0.32 (−1.50, 0.87)	−0.38 (−1.58, 0.83)	0.523	−0.52 (−1.93, 0.92)	0.467
**n-6**	0.17 (−0.61, 0.95)	0.14 (−0.63, 0.91)	0.724		
**Waist/hip ratio**					
**n-3**	−0.010 (−0.023, 0.003)	−1.06 (−2.40, 0.29)	0.119	−0.83 (−2.14, 0.49)	0.209
**n-6**	−0.002 (−0.010, 0.007)	−0.24 (−1.15, 0.69)	0.609		
**Waist/height ratio**					
**n-3**	−0.002 (−0.009, 0.005)	−0.33 (−1.51, 0.86)	0.574	−0.53 (−1.95, 0.91)	0.457
**n-6**	0.001 (−0.003, 0.006)	0.20 (−0.55, 0.95)	0.593		

Variable	Absolute (n = 38)[2]	Relative (n = 38)[3]	P-value[4]	bTXbSEX[5]	P-value[5]
**Visceral fat area, cm^2**					
n-3	−11.9 (−24.7, 0.98)	−6.44 (−15.5, 3.57)	0.192	−2.06 (−13.6, 11.0)	0.738
n-6	−8.57 (−21.4, 4.28)	−4.47 (−13.7, 5.75)	0.367		
**Fat-free mass, kg**					
n-3	−0.38 (−1.47, 0.71)	−0.78 (−2.74, 1.21)	0.428	−0.29 (−1.99, 1.45)	0.737
n-6	0.16 (−1.17, 1.49)	−0.50 (−2.59, 1.64)	0.636		
**Liver function markers, serum**					
**ALAT, U/L**					
n-3	−0.25 (−5.78, 5.28)	−3.72 (−18.4, 13.5)	0.643	−6.57 (−20.9, 10.3)	0.412
n-6	1.28 (−4.09, 6.65)	3.05 (−12.1, 20.8)	0.703		
**Albumin, g/L**					
n-3	0.81 (−0.56, 2.17)	1.72 (−1.33, 4.87)	0.263	0.30 (−2.49, 3.16)	0.832
n-6	0.65 (−0.65, 1.96)	1.42 (−1.49, 4.43)	0.332		
**ALP, U/L**					
n-3	1.85 (−2.19, 5.89)	1.94 (−3.84, 8.05)	0.509	0.033 (−8.74, 9.64)	0.994
n-6	1.53 (−2.51, 5.58)	1.90 (−3.87, 8.02)	0.516		
**ASAT, U/L**					
n-3	−1.08 (−4.11, 1.94)	−5.61 (−15.9, 5.95)	0.318	−7.30 (−18.6, 5.53)	0.243
n-6	0.99 (−2.04, 4.02)	1.83 (−9.28, 14.3)	0.752		
**Bile acids, µmol/L**					
n-3	−0.76 (−1.94, 0.41)	−25.1 (−46.1, 4.10)	0.083	−4.55 (−32.1, 34.1)	0.783
n-6	−1.12 (−2.30, 0.050)	−21.5 (−44.0, 10.1)	0.155		
**Bilirubin, µmol/L**					
n-3	−0.42 (−1.81, 0.96)	−5.98 (−22.9, 14.7)	0.533	0.058 (−22.9, 29.8)	0.996
n-6	−0.034 (−1.42, 1.35)	−6.04 (−23.0, 14.6)	0.529		
**CK, U/L**					
n-3	−11.1 (−37.8, 15.7)	−17.7 (−35.3, 4.72)	0.110	−3.98 (−23.3, 20.2)	0.715
n-6	−9.62 (−36.5, 17.3)	−14.3 (−32.4, 8.72)	0.196		
**GGT, U/L**					
n-3	−4.56 (−8.07, −1.04)	−15.8 (−28.6, −0.73)	0.041	−3.87 (−18.0, 12.8)	0.618
n-6	−1.39 (−6.73, 3.94)	−12.4 (−25.7, 3.27)	0.111		
**LD, U/L**					
n-3	1.66 (−5.31, 8.63)	1.14 (−3.46, 5.96)	0.625	−0.80 (−6.90, 5.69)	0.797
n-6	2.56 (−4.41, 9.53)	1.96 (−2.68, 6.82)	0.404		
**Additional hormones, serum**					
**Estrogen, pmol/L**					
n-3	−16.0 (−29.6, −2.37)	−14.5 (−29.4, 3.48)	0.104	−2.25 (−36.4, 50.3)	0.915
n-6	−34.4 (−103, 33.9)	−12.5 (−36.9, 21.2)	0.409		
**Testosterone, nmol/L**					
n-3	−2.18 (−4.32, −0.030)	−27.0 (−51.5, 9.89)	0.127	−3.38 (−17.5, 13.2)	0.662
n-6	−1.80 (−3.95, 0.35)	−24.5 (−49.8, 13.7)	0.173		
**SHBG, nmol/L**					
n-3	−2.19 (−10.1, 5.68)	−0.23 (−13.4, 14.9)	0.973	−8.74 (−19.7, 3.75)	0.156
n-6	4.07 (−3.80, 11.9)	9.32 (−5.06, 25.9)	0.208		
**FAI**					
n-3	−4.00 (−10.4, 2.38)	−28.6 (−57.3, 19.5)	0.192	5.55 (−11.9, 26.5)	0.548
n-6	−5.68 (−12.1, 0.71)	−32.4 (−59.6, 13.2)	0.132		
**FSH, IU/L**					
n-3	−1.81 (−6.05, 2.43)	−10.8 (−30.3, 14.3)	0.357	−10.4 (−37.5, 28.4)	0.537
n-6	2.35 (−2.59, 7.30)	−0.34 (−25.8, 33.8)	0.981		
**TSH, mU/L**					
n-3	−0.16 (−0.48, 0.15)	−1.60 (−19.2, 19.8)	0.869	−2.81 (−23.5, 23.5)	0.810
n-6	0.030 (−0.28, 0.34)	1.25 (−16.8, 23.2)	0.899		
**FT4, pmol/L**					
n-3	0.44 (−0.44, 1.33)	2.45 (−2.69, 7.87)	0.346	2.34 (−3.95, 9.04)	0.464
n-6	−0.015 (−0.89, 0.86)	0.11 (−4.91, 5.40)	0.965		
**IGF-1, nmol/L**					
n-3	−3.54 (−7.46, 0.37)	−7.89 (−22.7, 9.70)	0.346	3.60 (−14.7, 25.9)	0.714
n-6	−3.67 (−7.58, 0.24)	−11.1 (−22.4, 1.80)	0.087		
**Vitamin D$_3$, nmol/L**					
n-3	6.15 (0.35, 11.9)	7.07 (−2.10, 17.1)	0.130	1.14 (−9.51, 13.0)	0.837
n-6	3.64 (−2.16, 9.44)	5.86 (−3.16, 15.7)	0.203		
**Ketone bodies, serum**					
**3-hydroxybutyrate, µmol/L**					
n-3	36.0 (−2.65, 74.7)	54.1 (−7.53, 157)	0.095	43.8 (−17.1, 149)	0.189
n-6	22.4 (−20.5, 65.3)	7.12 (−35.8, 78.7)	0.786		

Variable	Absolute (n = 38)[2]	Relative (n = 38)[3]	P-value[4]	bTXbSEX[5]	P-value[5]
**Acetoacetate, µmol/L**					
**n-3**	11.8 (−7.37, 31.0)	20.6 (−19.7, 81.2)	0.356	17.4 (−21.3, 75.0)	0.421
**n-6**	5.37 (−10.7, 21.4)	2.77 (−31.7, 54.6)	0.893		

[1] Pooled period data of fasting blood levels and other measurements at follow-up were analyzed with ANCOVA mixed models including the interaction 'treatment × sex' and its daughter terms as fixed effects and 'subjects' as the random factor in the random intercept models. The models were adjusted for period-specific baselines (within-subject effect) and the main effect of period. Additionally, we included subject-averaged baselines (between-subject effect) as a fixed covariable to control for cross-level bias. Values are negative/positive when the baseline- and period-adjusted follow-up scores were lower/higher among females compared to males. Abbreviations: AA, arachidonic acid; ALA, α-linolenic acid; ALAT, alanine aminotransferase; Apo, apolipoprotein; ALP, alkaline phosphatase; ASAT, aspartate aminotransferase; bTXbSEX, between-treatment between-sex; CK, creatine kinase; DGLA, dihomo-γ-linolenic acid; DHA, docosahexaenoic acid; DPA, docosapentaenoic acid; EPA, eicosapentaenoic acid; FAI, free androgen index; FC, free cholesterol; FSH, follicle-stimulating hormone; FT4, free thyroxine; GLA, γ-linolenic acid; GGT, γ-glutamyl transpeptidase; HbA1c, glycated hemoglobin; HDLs, HDL particles; HDL-C, HDL cholesterol; HOMA2-IR, homeostasis model assessment of insulin resistance index 2 (computer model); HOMA2-%B, homeostasis model assessment of β-cell function index 2 (computer model); HOMA2-%S, homeostasis model assessment of insulin sensitivity index 2 (computer model); IDLs, intermediate-density lipoprotein particles; IGF-1, insulin-like growth factor-1; INCP, insulin C-peptide; LA, linoleic acid; LD, lactate dehydrogenase; LDLs, LDL particles; LDL-C, LDL cholesterol; Lp(a), lipoprotein (a); LP-IR, lipoprotein-based insulin resistance index; n-3, omega-3 PUFAs; n-6, omega-6 PUFAs; NEFAs, non-esterified fatty acids; non-HDL-C, non-HDL cholesterol; PLs, phospholipids; PUFAs, polyunsaturated fatty acids; QUICKI, quantitative insulin sensitivity check index; RBCMs, red blood cell membranes; rQUICKI, revised QUICKI; SHBG, sex hormone-binding globulin; TAGs, triacylglycerols; TC, total cholesterol; TRL-C, TAG-rich lipoprotein cholesterol; TSH, thyroid-stimulating hormone; VLDLs, VLDL particles; wt%, weight percentage of total fatty acids.
[2] Absolute between-sex (females vs. males) differences (95% CIs) in baseline- and period-adjusted follow-up scores from the mixed models.
[3] Relative between-sex (females vs. males) differences as percentages (95% CIs) in baseline- and period-adjusted follow-up scores calculated from the model estimates: $\% = (\exp^{\text{estimate}} - 1) \times 100$. Data were transformed by the natural logarithm before the analyses.
[4] P-values for relative between-sex differences.
[5] Relative effect estimates as percentages (95% CIs; first column) and p-values (second column) for between-treatment (bTX) differences in between-sex (bSEX) responses (females vs. males after n-3 vs. n-6; from the two-way interaction term 'treatment × sex').

SUPPLEMENTARY TABLE 7 Relative sex-specific differences at pre-treatment baseline in lipoprotein-lipid-apolipoprotein profiles[1]

Variable and treatment	Females[2]	Males[2]	Relative difference[3]	P-value[4]
**Lipoprotein subfractions, plasma**				
**Total VLDLs, nmol/L**	33.2 (15.1, 73.1)	50.8 (32.1, 80.3)	−34.5 (−57.4, 0.64)	0.061
**Large VLDLs, nmol/L**	1.79 (0.46, 6.90)	3.75 (1.36, 10.3)	−52.2 (−77.2, 0.40)	0.059
**Medium VLDLs, nmol/L**	15.0 (7.27, 31.1)	19.7 (8.59, 45.3)	−23.9 (−54.2, 26.6)	0.300
**Small VLDLs, nmol/L**	15.8 (5.98, 42.0)	23.4 (14.9, 36.6)	−32.2 (−60.6, 16.7)	0.170
**Total LDLs, nmol/L**	1256 (1020, 1546)	1371 (1079, 1743)	−8.41 (−20.8, 5.89)	0.243
**IDLs, nmol/L**	262 (159, 432)	225 (115, 442)	16.4 (−21.2, 71.8)	0.450
**Large LDLs, nmol/L**	567 (417, 770)	441 (299, 651)	28.5 (1.53, 62.6)	0.044
**Small LDLs, nmol/L**	275 (91.5, 824)	635 (425, 949)	−56.7 (−75.4, −24.1)	0.006
**Total HDLs, µmol/L**	32.3 (28.3, 36.9)	28.6 (25.1, 32.7)	13.0 (3.80, 23.0)	0.008
**Large HDLs, µmol/L**	9.47 (6.35, 14.1)	4.28 (2.70, 6.79)	121 (67.5, 193)	<0.001
**Medium HDLs, µmol/L**	5.74 (2.83, 11.6)	3.90 (1.52, 9.96)	38.7 (−15.6, 93.1)	0.171
**Small HDLs, µmol/L**	14.8 (10.1, 21.5)	18.6 (15.9, 21.7)	−20.7 (−34.8, −3.60)	0.026
**VLDL size, nm**	47.9 (40.2, 57.2)	49.5 (42.5, 57.7)	−3.24 (−12.8, 7.39)	0.540
**LDL size, nm**	21.4 (20.6, 22.1)	20.9 (20.3, 21.5)	2.00 (−0.072, 4.11)	0.066
**HDL size, nm**	9.70 (9.11, 10.3)	8.88 (8.52, 9.27)	9.13 (5.37, 13.0)	<0.001
**Lp(a) and blood lipids, serum**				
**Lp(a), mg/L**	122 (49.7, 297)	86.7 (31.2, 241)	40.2 (−24.6, 161)	0.292
**TAGs, mmol/L**	1.18 (0.78, 1.80)	1.39 (0.90, 2.15)	−14.7 (−35.2, 12.1)	0.261
**NEFAs, mmol/L**	0.60 (0.38, 0.96)	0.43 (0.23, 0.79)	40.5 (−1.79, 101)	0.071
**PLs, mmol/L**	3.08 (2.82, 3.36)	2.70 (2.34, 3.13)	13.8 (5.75, 22.4)	0.001
**TC, mmol/L**	6.06 (5.42, 6.77)	5.41 (4.57, 6.40)	12.0 (2.64, 22.3)	0.015
**FC, mmol/L**	1.67 (1.48, 1.87)	1.49 (1.25, 1.78)	11.7 (1.88, 22.5)	0.024
**LDL-C, mmol/L**	3.98 (3.35, 4.72)	3.73 (3.01, 4.62)	6.61 (−6.04, 21.0)	0.327
**HDL-C, mmol/L**	1.70 (1.25, 2.32)	1.20 (0.98, 1.45)	42.1 (19.7, 68.8)	<0.001
**Non-HDL-C, mmol/L**	4.25 (3.50, 5.15)	4.17 (3.34, 5.19)	1.95 (−10.8, 16.5)	0.779
**TRL-C, mmol/L**	0.25 (0.12, 0.52)	0.40 (0.24, 0.66)	−38.8 (−59.9, −6.81)	0.028
**TAG/HDL-C ratio**	0.70 (0.35, 1.40)	1.16 (0.68, 2.00)	−40.0 (−59.3, −11.5)	0.014
**TC/HDL-C ratio**	3.57 (2.62, 4.86)	4.52 (3.50, 5.84)	−21.2 (−34.0, −5.84)	0.013
**Apolipoproteins, serum**				
**ApoB, g/L**	1.10 (0.93, 1.29)	1.08 (0.89, 1.30)	2.00 (−8.96, 14.3)	0.735
**ApoA-I, g/L**	1.66 (1.42, 1.93)	1.37 (1.22, 1.54)	20.6 (10.9, 31.2)	<0.001
**ApoA-II, g/L**	0.46 (0.43, 0.50)	0.47 (0.43, 0.52)	−1.31 (−6.66, 4.34)	0.644
**ApoC-II, g/L**	0.22 (0.18, 0.27)	0.20 (0.14, 0.28)	11.7 (−6.38, 33.2)	0.228
**ApoC-III, g/L**	0.32 (0.26, 0.39)	0.26 (0.19, 0.36)	23.1 (4.22, 45.4)	0.019
**ApoE, g/L**	0.087 (0.070, 0.11)	0.089 (0.068, 0.12)	−1.81 (−16.2, 15.1)	0.822
**ApoB/ApoA-I ratio**	0.66 (0.51, 0.86)	0.78 (0.63, 0.98)	−15.4 (−27.3, −1.61)	0.037
**ApoC-II/ApoC-III ratio**	0.68 (0.60, 0.77)	0.75 (0.63, 0.88)	−9.29 (−17.6, −0.11)	0.055

[1] Fasting pre-treatment blood levels were analyzed with GLS models adjusted for heterogeneity of variance by using the *gls* function in the R package *nlme* v3.1-157. Data were transformed by the natural logarithm before the analyses. Total VLDLs and large VLDLs also include chylomicrons if present. Abbreviations: Apo, apolipoprotein; FC, free cholesterol; GLS, generalized least squares; HDLs, high-density lipoprotein particles; HDL-C, HDL cholesterol; IDLs, intermediate-density lipoprotein particles; LDLs, low-density lipoprotein particles; LDL-C, LDL cholesterol; Lp(a), lipoprotein (a); NEFAs, non-esterified fatty acids; non-HDL-C, non-HDL cholesterol; PLs, phospholipids; TAGs, triacylglycerols; TC, total cholesterol; TRL-C, TAG-rich lipoprotein cholesterol; VLDLs, very-low-density lipoprotein particles.
[2] Values are geometric means (1 SD ranges) of fasting blood levels measured at the first baseline visit before any intervention.
[3] Relative model-adjusted differences (95% CIs) between sexes (females vs. males) as percentages calculated from the model estimates: $\% = (\exp^{estimate} - 1) \times 100$.
[4] P-values for relative between-sex differences.

SUPPLEMENTARY TABLE 8 Absolute sex-specific differences at pre-treatment baseline in lipoprotein-lipid-apolipoprotein profiles[1]

Variable and treatment	Females[2]	Males[2]	Absolute difference[3]	P-value[4]
**Lipoprotein subfractions, plasma**				
**Total VLDLs, nmol/L**	42.6 (26.7)	55.6 (23.2)	−13.0 (−28.8, 2.78)	0.115
**Large VLDLs, nmol/L**	3.73 (4.85)	5.61 (4.62)	−1.88 (−4.89, 1.13)	0.229
**Medium VLDLs, nmol/L**	19.0 (13.4)	26.6 (20.3)	−7.56 (−18.3, 3.17)	0.176
**Small VLDLs, nmol/L**	22.7 (16.9)	25.7 (11.6)	−3.01 (−12.3, 6.27)	0.529
**Total LDLs, nmol/L**	1282 (271)	1410 (348)	−128 (−332, 75.0)	0.224
**IDLs, nmol/L**	294 (144)	275 (179)	18.3 (−87.4, 124)	0.736
**Large LDLs, nmol/L**	590 (164)	469 (155)	121 (16.9, 225)	0.029
**Small LDLs, nmol/L**	435 (371)	686 (283)	−251 (−457, −46.3)	0.021
**Total HDLs, µmol/L**	32.6 (4.29)	28.9 (4.08)	3.72 (1.06, 6.38)	0.009
**Large HDLs, µmol/L**	10.2 (3.76)	4.74 (2.20)	5.43 (3.38, 7.48)	<0.001
**Medium HDLs, µmol/L**	6.79 (3.29)	5.29 (3.68)	1.50 (−0.75, 3.74)	0.200
**Small HDLs, µmol/L**	15.6 (4.89)	18.8 (2.94)	−3.19 (−5.87, −0.51)	0.025
**VLDL size, nm**	48.7 (9.01)	50.1 (7.50)	−1.42 (−6.62, 3.78)	0.596
**LDL size, nm**	21.4 (0.77)	20.9 (0.59)	0.42 (−0.005, 0.85)	0.060
**HDL size, nm**	9.71 (0.60)	8.89 (0.38)	0.82 (0.49, 1.16)	<0.001
**Lp(a) and blood lipids, serum**				
**Lp(a), mg/L**	172 (148)	151 (185)	21.6 (−87.4, 131)	0.700
**TAGs, mmol/L**	1.29 (0.62)	1.52 (0.70)	−0.23 (−0.66, 0.20)	0.296
**NEFAs, mmol/L**	0.66 (0.27)	0.50 (0.26)	0.16 (−0.013, 0.33)	0.078
**PLs, mmol/L**	3.09 (0.28)	2.73 (0.41)	0.36 (0.12, 0.59)	0.005
**TC, mmol/L**	6.09 (0.73)	5.48 (0.96)	0.61 (0.052, 1.17)	0.039
**FC, mmol/L**	1.68 (0.21)	1.52 (0.28)	0.16 (0.002, 0.32)	0.055
**LDL-C, mmol/L**	4.03 (0.74)	3.81 (0.86)	0.22 (−0.30, 0.74)	0.414
**HDL-C, mmol/L**	1.77 (0.53)	1.22 (0.24)	0.56 (0.28, 0.84)	<0.001
**Non-HDL-C, mmol/L**	4.32 (0.82)	4.26 (0.99)	0.054 (−0.54, 0.64)	0.859
**TRL-C, mmol/L**	0.29 (0.27)	0.45 (0.22)	−0.16 (−0.31, −0.004)	0.052
**TAG/HDL-C ratio**	0.88 (0.65)	1.34 (0.78)	−0.47 (−0.94, 0.000)	0.058
**TC/HDL-C ratio**	3.73 (1.13)	4.67 (1.24)	−0.94 (−1.71, −0.18)	0.020
**Apolipoproteins, serum**				
**ApoB, g/L**	1.11 (0.18)	1.09 (0.21)	0.016 (−0.11, 0.14)	0.805
**ApoA-I, g/L**	1.67 (0.25)	1.38 (0.16)	0.29 (0.15, 0.43)	<0.001
**ApoA-II, g/L**	0.46 (0.031)	0.47 (0.044)	−0.007 (−0.033, 0.018)	0.580
**ApoC-II, g/L**	0.22 (0.046)	0.21 (0.073)	0.015 (−0.023, 0.054)	0.435
**ApoC-III, g/L**	0.33 (0.073)	0.27 (0.086)	0.054 (0.002, 0.11)	0.050
**ApoE, g/L**	0.089 (0.020)	0.092 (0.028)	−0.003 (−0.019, 0.013)	0.720
**ApoB/ApoA-I ratio**	0.68 (0.17)	0.80 (0.19)	−0.12 (−0.24, −0.006)	0.046
**ApoC-II/ApoC-III ratio**	0.68 (0.083)	0.76 (0.12)	−0.074 (−0.14, −0.006)	0.040

[1] Fasting pre-treatment blood levels were analyzed with GLS models adjusted for heterogeneity of variance by using the *gls* function in the R package *nlme* v3.1-157. Total VLDLs and large VLDLs also include chylomicrons if present. Abbreviations: Apo, apolipoprotein; FC, free cholesterol; GLS, generalized least squares; HDLs, high-density lipoprotein particles; HDL-C, HDL cholesterol; IDLs, intermediate-density lipoprotein particles; LDLs, low-density lipoprotein particles; LDL-C, LDL cholesterol; Lp(a), lipoprotein (a); n-3, omega-3 PUFAs; n-6, omega-6 PUFAs; NEFAs, non-esterified fatty acids; non-HDL-C, non-HDL cholesterol; PLs, phospholipids; PUFAs, polyunsaturated fatty acids; TAGs, triacylglycerols; TC, total cholesterol; TRL-C, TAG-rich lipoprotein cholesterol; VLDLs, very-low-density lipoprotein particles.

[2] Values are arithmetic means (SDs) of fasting blood levels at the first baseline measurement before any intervention.

[3] Absolute model-adjusted differences between sexes (females vs. males) at baseline.

[4] P-values for absolute between-sex differences.

SUPPLEMENTARY TABLE 9 Sex-specific responses in relative changes for circulating levels of lipoprotein subfractions, lipids, and apolipoproteins after seven wk of supplementation with n-3 or n-6 PUFAs[1]

Variable and treatment	Baseline[2]	Follow-up[2]	Relative change[3]	Time[4]	wTXbSEX[5]	bTXwSEX[6]
**Lipoprotein subfractions, plasma**						
**Total VLDLs, nmol/L**						
**n-3 : females**	35.5 (18.8, 67.1)	22.4 (9.50, 53.0)	−36.2 (−57.9, −3.51)	0.034	0.719	<0.001
**n-3 : males**	51.3 (31.0, 85.0)	30.4 (14.7, 63.0)	−42.2 (−59.4, −17.8)	0.003		0.001
**n-6 : females**	33.4 (16.6, 67.4)	47.7 (31.2, 72.9)	+37.0 (10.5, 70.0)	0.004	0.041	
**n-6 : males**	52.5 (37.0, 74.4)	53.0 (38.0, 73.8)	+2.13 (−15.0, 22.7)	0.820		
**Large VLDLs, nmol/L**						
**n-3 : females**	1.64 (0.42, 6.42)	0.96 (0.24, 3.87)	−52.5 (−71.2, −21.7)	0.004	0.321	<0.001
**n-3 : males**	4.71 (1.95, 11.4)	1.51 (0.53, 4.35)	−65.7 (−77.2, −48.3)	<0.001		<0.001
**n-6 : females**	2.52 (0.63, 10.1)	2.74 (1.00, 7.54)	+34.9 (−18.2, 122)	0.238	0.254	
**n-6 : males**	4.14 (1.43, 12.0)	4.09 (2.01, 8.35)	−7.17 (−38.3, 39.8)	0.719		
**Medium VLDLs, nmol/L**						
**n-3 : females**	14.2 (6.43, 31.6)	10.2 (3.91, 26.9)	−21.7 (−53.1, 30.8)	0.347	0.834	0.434
**n-3 : males**	20.2 (10.0, 40.6)	14.9 (5.70, 39.0)	−15.9 (−45.7, 30.2)	0.434		0.346
**n-6 : females**	12.2 (5.12, 29.2)	12.6 (5.47, 29.0)	−5.03 (−31.2, 31.1)	0.752	0.723	
**n-6 : males**	17.6 (8.46, 36.6)	17.8 (10.5, 29.9)	+2.55 (−22.6, 35.8)	0.859		
**Small VLDLs, nmol/L**						
**n-3 : females**	13.6 (4.96, 37.1)	8.41 (2.39, 29.5)	−43.5 (−70.4, 7.71)	0.082	0.578	<0.001
**n-3 : males**	25.1 (15.6, 40.3)	8.90 (1.99, 39.7)	−55.7 (−75.1, −21.2)	0.006		0.002
**n-6 : females**	16.5 (6.47, 41.8)	28.5 (18.6, 43.8)	+97.1 (38.2, 181)	<0.001	0.021	
**n-6 : males**	22.5 (9.46, 53.4)	26.4 (14.2, 48.9)	+14.2 (−15.6, 54.6)	0.386		
**Total LDLs, nmol/L**						
**n-3 : females**	1250 (958, 1630)	1280 (1027, 1596)	+3.50 (−4.50, 12.2)	0.398	0.283	0.035
**n-3 : males**	1385 (1088, 1763)	1510 (1162, 1961)	+9.24 (3.08, 15.8)	0.003		<0.001
**n-6 : females**	1224 (920, 1628)	1160 (888, 1514)	−6.25 (−13.5, 1.60)	0.115	0.665	
**n-6 : males**	1373 (1083, 1741)	1324 (1047, 1675)	−4.19 (−9.58, 1.53)	0.147		
**IDLs, nmol/L**						
**n-3 : females**	216 (117, 396)	75.4 (19.0, 298)	−65.4 (−81.5, −35.6)	0.001	0.398	0.323
**n-3 : males**	198 (94.7, 413)	88.9 (18.1, 437)	−51.0 (−71.1, −16.7)	0.009		0.515
**n-6 : females**	213 (107, 425)	111 (53.3, 231)	−49.5 (−72.9, −5.96)	0.032	0.657	
**n-6 : males**	197 (91.7, 425)	113 (36.7, 349)	−39.4 (−64.3, 2.96)	0.064		
**Large LDLs, nmol/L**						
**n-3 : females**	558 (384, 810)	735 (521, 1037)	+32.5 (9.53, 60.3)	0.004	0.633	0.001
**n-3 : males**	458 (316, 663)	541 (383, 763)	+24.7 (5.64, 47.1)	0.010		0.001
**n-6 : females**	561 (391, 805)	519 (323, 836)	−6.37 (−22.6, 13.3)	0.495	0.922	
**n-6 : males**	408 (257, 647)	409 (307, 544)	−7.53 (−21.6, 9.12)	0.350		
**Small LDLs, nmol/L**						
**n-3 : females**	284 (94.2, 857)	237 (77.1, 726)	−13.7 (−40.9, 26.2)	0.444	0.140	0.654
**n-3 : males**	666 (465, 956)	783 (593, 1032)	+16.3 (3.22, 31.1)	0.014		0.253
**n-6 : females**	264 (80.7, 867)	267 (74.7, 954)	−2.59 (−33.4, 42.4)	0.891	0.611	
**n-6 : males**	688 (472, 1003)	726 (533, 989)	+7.92 (−4.25, 21.6)	0.209		
**Total HDLs, µmol/L**						
**n-3 : females**	31.6 (27.8, 36.0)	28.4 (24.5, 32.9)	−11.3 (−16.4, −5.80)	<0.001	0.036	<0.001
**n-3 : males**	28.7 (24.7, 33.3)	28.0 (24.4, 32.2)	−3.29 (−8.34, 2.03)	0.218		0.005
**n-6 : females**	32.3 (27.5, 37.9)	33.7 (28.8, 39.5)	+5.41 (−0.71, 11.9)	0.084	0.676	
**n-6 : males**	29.2 (25.8, 32.9)	30.0 (25.2, 35.8)	+3.63 (−1.77, 9.34)	0.189		
**Large HDLs, µmol/L**						
**n-3 : females**	9.32 (6.08, 14.3)	10.1 (6.39, 16.1)	+7.44 (−5.54, 22.2)	0.272	0.911	0.255
**n-3 : males**	4.51 (2.76, 7.35)	4.96 (2.73, 9.03)	+6.42 (−4.65, 18.8)	0.264		0.183
**n-6 : females**	9.75 (6.27, 15.2)	9.75 (6.25, 15.2)	−1.93 (−13.8, 11.5)	0.764	0.911	
**n-6 : males**	4.99 (3.43, 7.27)	4.66 (2.96, 7.34)	−2.87 (−13.0, 8.42)	0.601		
**Medium HDLs, µmol/L**						
**n-3 : females**	6.73 (3.83, 11.8)	3.82 (1.39, 10.5)	−31.2 (−54.4, 3.85)	0.075	0.097	0.003
**n-3 : males**	4.12 (1.61, 10.6)	4.12 (1.78, 9.54)	+7.96 (−24.1, 53.6)	0.668		0.815
**n-6 : females**	7.52 (3.76, 15.0)	7.61 (4.55, 12.7)	+25.0 (−5.59, 65.4)	0.118	0.309	
**n-6 : males**	4.71 (2.11, 10.5)	4.37 (1.62, 11.8)	+3.74 (−18.5, 32.0)	0.763		
**Small HDLs, µmol/L**						
**n-3 : females**	14.7 (11.9, 18.1)	11.0 (6.12, 19.6)	−19.6 (−35.5, 0.27)	0.053	0.186	0.006
**n-3 : males**	18.1 (15.5, 21.2)	16.9 (14.7, 19.6)	−5.92 (−13.0, 1.70)	0.124		<0.001
**n-6 : females**	12.0 (7.21, 20.1)	13.9 (9.08, 21.2)	+4.36 (−3.54, 12.9)	0.285	0.848	
**n-6 : males**	17.9 (15.3, 21.1)	19.0 (16.4, 22.1)	+5.45 (−1.99, 13.5)	0.154		

Variable and treatment	Baseline[2]	Follow-up[2]	Relative change[3]	Time[4]	wTXbSEX[5]	bTXwSEX[6]
**VLDL size, nm**						
**n-3 : females**	45.8 (38.7, 54.1)	48.7 (41.7, 57.0)	+1.69 (−7.19, 11.4)	0.717	0.323	0.432
**n-3 : males**	50.9 (44.5, 58.2)	48.2 (41.4, 56.1)	−3.83 (−9.77, 2.50)	0.227		0.822
**n-6 : females**	50.2 (41.2, 61.2)	47.2 (42.0, 52.9)	−1.57 (−10.2, 7.84)	0.731	0.758	
**n-6 : males**	49.4 (41.5, 58.7)	48.5 (42.9, 54.8)	−3.27 (−9.24, 3.10)	0.305		
**LDL size, nm**						
**n-3 : females**	21.4 (20.8, 22.0)	21.3 (20.8, 21.8)	−0.21 (−1.25, 0.84)	0.692	0.962	0.029
**n-3 : males**	20.9 (20.4, 21.5)	20.9 (20.6, 21.2)	−0.18 (−1.06, 0.72)	0.695		0.149
**n-6 : females**	21.3 (20.5, 22.2)	21.1 (20.4, 21.8)	−1.26 (−2.29, −0.22)	0.018	0.472	
**n-6 : males**	20.9 (20.3, 21.4)	20.8 (20.4, 21.2)	−0.77 (−1.65, 0.12)	0.089		
**HDL size, nm**						
**n-3 : females**	9.73 (9.15, 10.3)	9.97 (9.29, 10.7)	+2.09 (0.46, 3.74)	0.012	0.045	0.023
**n-3 : males**	8.97 (8.56, 9.40)	9.02 (8.58, 9.49)	−0.093 (−1.45, 1.28)	0.893		0.243
**n-6 : females**	9.81 (9.12, 10.6)	9.76 (9.15, 10.4)	−0.046 (−1.64, 1.57)	0.955	0.368	
**n-6 : males**	9.07 (8.71, 9.44)	8.94 (8.59, 9.31)	−1.00 (−2.35, 0.36)	0.146		
**Lp(a) and blood lipids, serum**						
**Lp(a), mg/L**						
**n-3 : females**	124 (50.1, 306)	124 (54.4, 285)	+2.57 (−9.09, 15.7)	0.677	0.765	0.005
**n-3 : males**	83.9 (31.0, 227)	84.5 (30.2, 236)	+0.15 (−9.62, 11.0)	0.977		0.950
**n-6 : females**	119 (48.0, 295)	102 (40.5, 258)	−15.8 (−25.4, −5.05)	0.006	0.028	
**n-6 : males**	84.3 (29.8, 239)	84.8 (30.0, 240)	+0.52 (−9.29, 11.4)	0.920		
**TAGs, mmol/L**						
**n-3 : females**	1.12 (0.69, 1.80)	0.96 (0.62, 1.49)	−15.6 (−26.9, −2.50)	0.022	0.769	0.002
**n-3 : males**	1.42 (0.92, 2.19)	1.15 (0.82, 1.60)	−17.9 (−27.4, −7.23)	0.002		0.005
**n-6 : females**	1.17 (0.72, 1.90)	1.21 (0.81, 1.80)	+5.92 (−8.31, 22.4)	0.431	0.380	
**n-6 : males**	1.42 (0.93, 2.16)	1.36 (0.95, 1.95)	−2.64 (−13.9, 10.1)	0.667		
**NEFAs, mmol/L**						
**n-3 : females**	0.55 (0.32, 0.93)	0.57 (0.37, 0.89)	+6.43 (−21.3, 44.0)	0.683	0.065	0.686
**n-3 : males**	0.51 (0.33, 0.79)	0.35 (0.20, 0.61)	−26.7 (−43.3, −5.25)	0.018		0.256
**n-6 : females**	0.51 (0.32, 0.81)	0.54 (0.31, 0.94)	+0.87 (−23.5, 33.0)	0.951	0.301	
**n-6 : males**	0.37 (0.20, 0.68)	0.39 (0.24, 0.64)	−16.6 (−34.1, 5.47)	0.128		
**PLs, mmol/L**						
**n-3 : females**	3.07 (2.81, 3.35)	3.01 (2.76, 3.29)	−2.84 (−7.73, 2.32)	0.273	0.813	0.595
**n-3 : males**	2.75 (2.41, 3.13)	2.70 (2.37, 3.09)	−2.04 (−6.26, 2.36)	0.354		0.920
**n-6 : females**	3.13 (2.78, 3.53)	3.06 (2.71, 3.45)	−1.31 (−6.29, 3.92)	0.613	0.886	
**n-6 : males**	2.76 (2.38, 3.20)	2.71 (2.39, 3.08)	−1.80 (−6.02, 2.62)	0.416		
**TC, mmol/L**						
**n-3 : females**	6.09 (5.44, 6.82)	6.10 (5.50, 6.77)	+0.032 (−5.09, 5.43)	0.990	0.800	0.049
**n-3 : males**	5.45 (4.64, 6.40)	5.43 (4.62, 6.38)	−0.85 (−5.18, 3.68)	0.707		0.146
**n-6 : females**	6.11 (5.33, 7.00)	5.76 (4.98, 6.66)	−5.51 (−10.4, −0.42)	0.035	0.721	
**n-6 : males**	5.46 (4.56, 6.55)	5.24 (4.43, 6.20)	−4.33 (−8.51, 0.042)	0.052		
**FC, mmol/L**						
**n-3 : females**	1.69 (1.49, 1.91)	1.70 (1.53, 1.88)	−0.098 (−5.56, 5.68)	0.972	0.745	0.236
**n-3 : males**	1.53 (1.31, 1.79)	1.52 (1.28, 1.80)	−1.30 (−5.91, 3.53)	0.588		0.578
**n-6 : females**	1.70 (1.48, 1.96)	1.63 (1.43, 1.86)	−3.81 (−9.07, 1.75)	0.174	0.776	
**n-6 : males**	1.53 (1.26, 1.86)	1.50 (1.26, 1.77)	−2.78 (−7.32, 1.98)	0.244		
**LDL-C, mmol/L**						
**n-3 : females**	4.06 (3.38, 4.88)	4.06 (3.44, 4.80)	+0.91 (−5.54, 7.80)	0.787	0.673	0.012
**n-3 : males**	3.82 (3.13, 4.65)	3.80 (3.08, 4.67)	−0.95 (−6.36, 4.78)	0.738		0.166
**n-6 : females**	3.99 (3.25, 4.90)	3.70 (3.04, 4.51)	−8.00 (−13.9, −1.71)	0.014	0.486	
**n-6 : males**	3.81 (3.03, 4.78)	3.64 (2.94, 4.50)	−5.14 (−10.3, 0.35)	0.066		
**HDL-C, mmol/L**						
**n-3 : females**	1.69 (1.27, 2.26)	1.78 (1.34, 2.35)	+4.07 (−2.40, 11.0)	0.220	0.700	0.964
**n-3 : males**	1.19 (0.96, 1.47)	1.28 (1.05, 1.55)	+5.80 (0.17, 11.7)	0.043		0.129
**n-6 : females**	1.72 (1.23, 2.42)	1.78 (1.29, 2.45)	+3.93 (−2.53, 10.8)	0.237	0.606	
**n-6 : males**	1.21 (0.98, 1.48)	1.23 (0.99, 1.52)	+1.67 (−3.74, 7.38)	0.550		
**Non-HDL-C, mmol/L**						
**n-3 : females**	4.28 (3.48, 5.27)	4.23 (3.55, 5.04)	−0.74 (−6.82, 5.74)	0.816	0.637	0.011
**n-3 : males**	4.21 (3.40, 5.20)	4.10 (3.30, 5.10)	−2.69 (−7.79, 2.69)	0.317		0.241
**n-6 : females**	4.25 (3.39, 5.32)	3.88 (3.14, 4.79)	−9.00 (−14.6, −3.06)	0.004	0.427	
**n-6 : males**	4.20 (3.31, 5.33)	3.97 (3.20, 4.91)	−5.90 (−10.8, −0.70)	0.027		
**TRL-C, mmol/L**						
**n-3 : females**	0.21 (0.096, 0.44)	0.18 (0.094, 0.34)	−19.7 (−35.4, −0.19)	0.048	0.871	0.969
**n-3 : males**	0.35 (0.18, 0.66)	0.27 (0.14, 0.53)	−21.5 (−34.8, −5.60)	0.011		0.202
**n-6 : females**	0.24 (0.11, 0.51)	0.18 (0.087, 0.37)	−20.0 (−35.6, −0.62)	0.044	0.474	
**n-6 : males**	0.36 (0.20, 0.64)	0.31 (0.18, 0.52)	−11.3 (−26.3, 6.70)	0.201		

Variable and treatment	Baseline[2]	Follow-up[2]	Relative change[3]	Time[4]	wTXbSEX[5]	bTXwSEX[6]
**TAG/HDL-C ratio**						
**n-3 : females**	0.66 (0.32, 1.36)	0.54 (0.28, 1.07)	−18.9 (−29.6, −6.53)	0.004	0.663	<0.001
**n-3 : males**	1.19 (0.69, 2.06)	0.90 (0.58, 1.38)	−22.4 (−32.6, −10.6)	0.001		0.003
**n-6 : females**	0.68 (0.31, 1.48)	0.68 (0.35, 1.32)	+1.92 (−11.6, 17.5)	0.792	0.542	
**n-6 : males**	1.18 (0.69, 2.00)	1.11 (0.70, 1.77)	−4.20 (−16.8, 10.3)	0.549		
**TC/HDL-C ratio**						
**n-3 : females**	3.59 (2.64, 4.90)	3.43 (2.62, 4.49)	−3.88 (−9.69, 2.30)	0.211	0.539	0.060
**n-3 : males**	4.58 (3.52, 5.95)	4.25 (3.32, 5.45)	−6.30 (−11.1, −1.19)	0.017		0.871
**n-6 : females**	3.54 (2.55, 4.93)	3.24 (2.44, 4.32)	−9.09 (−14.6, −3.24)	0.003	0.408	
**n-6 : males**	4.53 (3.44, 5.96)	4.27 (3.35, 5.44)	−5.91 (−10.8, −0.79)	0.025		
**Apolipoproteins, serum**						
**ApoB, g/L**						
**n-3 : females**	1.10 (0.92, 1.31)	1.11 (0.98, 1.27)	+0.86 (−4.33, 6.33)	0.748	0.715	0.012
**n-3 : males**	1.08 (0.91, 1.30)	1.09 (0.89, 1.32)	−0.42 (−4.80, 4.15)	0.852		0.041
**n-6 : females**	1.11 (0.93, 1.33)	1.03 (0.87, 1.23)	−6.37 (−11.2, −1.29)	0.015	0.776	
**n-6 : males**	1.09 (0.89, 1.34)	1.03 (0.85, 1.26)	−5.43 (−9.59, −1.09)	0.015		
**ApoA-I, g/L**						
**n-3 : females**	1.64 (1.42, 1.88)	1.60 (1.41, 1.81)	−4.25 (−8.26, −0.065)	0.047	0.052	0.013
**n-3 : males**	1.36 (1.20, 1.54)	1.38 (1.21, 1.57)	+1.16 (−2.46, 4.93)	0.531		0.406
**n-6 : females**	1.66 (1.39, 1.99)	1.67 (1.42, 1.97)	+1.49 (−2.76, 5.93)	0.494	0.639	
**n-6 : males**	1.37 (1.22, 1.55)	1.38 (1.20, 1.59)	+2.84 (−0.85, 6.66)	0.131		
**ApoA-II, g/L**						
**n-3 : females**	0.47 (0.44, 0.51)	0.44 (0.37, 0.51)	−7.89 (−12.8, −2.73)	0.003	0.221	0.005
**n-3 : males**	0.47 (0.42, 0.53)	0.45 (0.40, 0.50)	−3.70 (−8.07, 0.88)	0.111		0.043
**n-6 : females**	0.47 (0.44, 0.51)	0.47 (0.42, 0.53)	−0.088 (−5.38, 5.50)	0.974	0.716	
**n-6 : males**	0.46 (0.41, 0.51)	0.47 (0.42, 0.53)	+1.24 (−3.36, 6.05)	0.601		
**ApoC-II, g/L**						
**n-3 : females**	0.22 (0.17, 0.27)	0.20 (0.16, 0.25)	−10.3 (−19.2, −0.47)	0.041	0.999	0.177
**n-3 : males**	0.21 (0.15, 0.28)	0.18 (0.14, 0.24)	−10.3 (−17.9, −1.99)	0.017		0.122
**n-6 : females**	0.22 (0.18, 0.27)	0.21 (0.17, 0.26)	−2.64 (−12.3, 8.05)	0.612	0.979	
**n-6 : males**	0.20 (0.14, 0.28)	0.20 (0.14, 0.27)	−2.82 (−11.1, 6.21)	0.525		
**ApoC-III, g/L**						
**n-3 : females**	0.31 (0.25, 0.39)	0.29 (0.23, 0.38)	−9.38 (−19.6, 2.10)	0.105	0.833	0.090
**n-3 : males**	0.28 (0.20, 0.39)	0.25 (0.19, 0.33)	−7.86 (−16.8, 2.01)	0.114		0.182
**n-6 : females**	0.34 (0.27, 0.41)	0.33 (0.26, 0.41)	+2.02 (−9.45, 14.9)	0.740	0.776	
**n-6 : males**	0.26 (0.19, 0.37)	0.27 (0.20, 0.37)	−0.25 (−9.90, 10.4)	0.961		
**ApoE, g/L**						
**n-3 : females**	0.088 (0.075, 0.10)	0.094 (0.078, 0.11)	+3.98 (−4.69, 13.4)	0.376	0.820	0.175
**n-3 : males**	0.092 (0.073, 0.12)	0.094 (0.075, 0.12)	+2.69 (−3.72, 9.54)	0.416		0.068
**n-6 : females**	0.092 (0.076, 0.11)	0.088 (0.071, 0.11)	−2.21 (−10.4, 6.68)	0.612	0.911	
**n-6 : males**	0.091 (0.069, 0.12)	0.089 (0.071, 0.11)	−2.81 (−8.88, 3.67)	0.384		
**ApoB/ApoA-I ratio**						
**n-3 : females**	0.67 (0.52, 0.87)	0.70 (0.57, 0.86)	+4.05 (−1.76, 10.2)	0.174	0.189	<0.001
**n-3 : males**	0.80 (0.64, 1.00)	0.79 (0.62, 1.00)	−1.06 (−5.78, 3.90)	0.667		0.035
**n-6 : females**	0.66 (0.51, 0.87)	0.62 (0.49, 0.78)	−7.52 (−12.7, −2.05)	0.008	0.741	
**n-6 : males**	0.80 (0.63, 1.01)	0.75 (0.59, 0.95)	−6.35 (−10.8, −1.66)	0.009		
**ApoC-II/ApoC-III ratio**						
**n-3 : females**	0.68 (0.61, 0.77)	0.66 (0.58, 0.76)	−1.03 (−6.93, 5.25)	0.739	0.681	0.306
**n-3 : males**	0.74 (0.61, 0.91)	0.73 (0.57, 0.94)	−2.68 (−7.65, 2.56)	0.307		0.970
**n-6 : females**	0.65 (0.59, 0.73)	0.64 (0.54, 0.75)	−4.57 (−10.3, 1.49)	0.135	0.613	
**n-6 : males**	0.75 (0.63, 0.90)	0.73 (0.59, 0.90)	−2.57 (−7.55, 2.67)	0.327		

[1] Fasting blood levels were analyzed with cLMMs adjusted for the main effects of period and subject-averaged baselines. Data were transformed by the natural logarithm before the analyses. Total VLDLs and large VLDLs also include chylomicrons if present. Abbreviations: Apo, apolipoprotein; bTXwSEX, between-treatment within-sex; FC, free cholesterol; HDLs, high-density lipoprotein particles; HDL-C, HDL cholesterol; IDLs, intermediate-density lipoprotein particles; LDLs, low-density lipoprotein particles; LDL-C, LDL cholesterol; cLMM, constrained linear mixed-effects model; Lp(a), lipoprotein (a); n-3, omega-3 PUFAs; n-6, omega-6 PUFAs; NEFAs, non-esterified fatty acids; non-HDL-C, non-HDL cholesterol; PLs, phospholipids; PUFAs, polyunsaturated fatty acids; TAGs, triacylglycerols; TC, total cholesterol; TRL-C, TAG-rich lipoprotein cholesterol; VLDLs, very-low-density lipoprotein particles; wTXbSEX, within-treatment between-sex.
[2] Values are geometric means (1 SD ranges) of fasting blood levels at baseline and follow-up within treatments and sexes.
[3] Relative model-adjusted mean change scores (95% CIs) from baseline to follow-up as percentages calculated from the model estimates: $\% = (\exp^{\text{estimate}} - 1) \times 100$.
[4] P-values for relative changes from baseline to follow-up within treatments and sexes (time effects).
[5] P-values for relative changes from baseline to follow-up within treatments and between sexes (sex differences in time effects). The first and second values refer to between-sex differences after the n-3 and n-6 interventions, respectively.
[6] P-values for relative changes from baseline to follow-up between treatments and within sexes (group differences in time effects within each stratum of sex). The first and second values refer to between-treatment differences in females and males, respectively.

frontiers

SUPPLEMENTARY TABLE 10 Sex-specific responses in absolute changes for circulating levels of lipoprotein subfractions, lipids, and apolipoproteins after seven wk of supplementation with n-3 or n-6 PUFAs[1]

Variable and treatment	Baseline[2]	Follow-up[2]	Absolute change[3]	Time[4]	wTXbSEX[5]	bTXwSEX[6]
**Lipoprotein subfractions, plasma**						
**Total VLDLs, nmol/L**						
**n-3 : females**	42.2 (24.0)	30.6 (23.4)	−10.9 (−23.2, 1.37)	0.081	0.405	0.001
**n-3 : males**	57.2 (25.4)	37.8 (22.6)	−17.7 (−28.1, −7.25)	0.001		0.001
**n-6 : females**	40.7 (23.8)	51.6 (19.8)	+10.1 (−2.13, 22.4)	0.104	0.220	
**n-6 : males**	55.7 (21.0)	55.6 (16.3)	+0.11 (−10.3, 10.5)	0.983		
**Large VLDLs, nmol/L**						
**n-3 : females**	3.54 (4.69)	1.98 (2.17)	−2.37 (−4.42, −0.32)	0.024	0.244	0.013
**n-3 : males**	6.45 (4.74)	2.45 (2.46)	−3.96 (−5.70, −2.22)	<0.001		<0.001
**n-6 : females**	5.15 (5.65)	4.19 (3.70)	−0.16 (−2.21, 1.89)	0.878	0.424	
**n-6 : males**	6.43 (5.72)	5.16 (3.77)	−1.25 (−2.99, 0.49)	0.158		
**Medium VLDLs, nmol/L**						
**n-3 : females**	18.8 (13.8)	14.5 (10.7)	−2.80 (−9.97, 4.36)	0.440	0.662	0.663
**n-3 : males**	25.3 (18.4)	21.1 (16.0)	−0.72 (−6.84, 5.40)	0.816		0.811
**n-6 : females**	16.0 (11.0)	16.4 (11.1)	−0.96 (−7.99, 6.07)	0.788	0.892	
**n-6 : males**	22.1 (14.5)	20.1 (10.1)	−1.60 (−7.72, 4.53)	0.606		
**Small VLDLs, nmol/L**						
**n-3 : females**	19.9 (15.0)	16.0 (19.4)	−5.48 (−14.5, 3.50)	0.229	0.319	0.001
**n-3 : males**	27.8 (13.0)	16.4 (14.3)	−11.5 (−19.3, −3.68)	0.004		0.001
**n-6 : females**	22.4 (15.7)	31.0 (12.6)	+10.1 (1.28, 18.9)	0.025	0.182	
**n-6 : males**	28.2 (15.1)	30.3 (13.3)	+2.26 (−5.22, 9.73)	0.551		
**Total LDLs, nmol/L**						
**n-3 : females**	1292 (351)	1309 (282)	+28.5 (−76.6, 134)	0.592	0.116	0.067
**n-3 : males**	1424 (345)	1559 (408)	+139 (49.5, 228)	0.003		<0.001
**n-6 : females**	1269 (336)	1197 (302)	−83.3 (−188, 21.8)	0.119	0.748	
**n-6 : males**	1410 (327)	1360 (324)	−60.9 (−150, 28.5)	0.180		
**IDLs, nmol/L**						
**n-3 : females**	249 (120)	131 (104)	−127 (−208, −45.8)	0.002	0.351	0.795
**n-3 : males**	252 (184)	176 (159)	−76.6 (−145, −7.74)	0.030		0.887
**n-6 : females**	266 (186)	141 (98.9)	−116 (−198, −35.5)	0.005	0.515	
**n-6 : males**	251 (160)	171 (136)	−81.4 (−150, −12.6)	0.021		
**Large LDLs, nmol/L**						
**n-3 : females**	587 (162)	774 (247)	+187 (89.5, 285)	<0.001	0.248	<0.001
**n-3 : males**	483 (140)	571 (186)	+113 (32.6, 194)	0.006		<0.001
**n-6 : females**	595 (207)	571 (235)	−16.2 (−114, 81.7)	0.744	0.706	
**n-6 : males**	443 (165)	426 (138)	−40.3 (−121, 40.2)	0.323		
**Small LDLs, nmol/L**						
**n-3 : females**	456 (399)	404 (389)	−46.1 (−159, 67.1)	0.421	0.064	0.212
**n-3 : males**	711 (276)	813 (245)	+94.3 (−1.97, 191)	0.055		0.358
**n-6 : females**	445 (381)	485 (428)	+35.0 (−78.2, 148)	0.541	0.911	
**n-6 : males**	736 (286)	762 (266)	+43.4 (−52.9, 140)	0.374		
**Total HDLs, µmol/L**						
**n-3 : females**	31.9 (4.18)	28.7 (4.21)	−3.64 (−5.42, −1.86)	<0.001	0.027	<0.001
**n-3 : males**	29.0 (4.47)	28.3 (3.91)	−0.96 (−2.53, 0.61)	0.229		0.004
**n-6 : females**	32.7 (5.32)	34.1 (5.23)	+1.80 (0.018, 3.58)	0.048	0.638	
**n-6 : males**	29.4 (3.64)	30.5 (5.27)	+1.23 (−0.34, 2.81)	0.124		
**Large HDLs, µmol/L**						
**n-3 : females**	10.1 (3.95)	11.1 (4.44)	+0.71 (−0.31, 1.73)	0.169	0.753	0.439
**n-3 : males**	5.06 (2.58)	5.77 (3.10)	+0.52 (−0.14, 1.18)	0.124		0.046
**n-6 : females**	10.7 (4.67)	10.6 (4.34)	+0.26 (−0.76, 1.28)	0.614	0.522	
**n-6 : males**	5.34 (2.05)	5.12 (2.27)	−0.13 (−0.79, 0.53)	0.689		
**Medium HDLs, µmol/L**						
**n-3 : females**	7.75 (4.15)	5.19 (3.19)	−2.99 (−4.75, −1.24)	0.001	0.022	<0.001
**n-3 : males**	5.54 (3.67)	5.43 (3.82)	−0.34 (−1.76, 1.08)	0.634		0.314
**n-6 : females**	8.62 (3.24)	8.51 (3.89)	+0.32 (−1.43, 2.08)	0.717	0.949	
**n-6 : males**	5.87 (3.17)	6.16 (4.28)	+0.39 (−1.02, 1.81)	0.583		
**Small HDLs, µmol/L**						
**n-3 : females**	15.0 (3.09)	12.3 (4.96)	−1.55 (−3.09, −0.010)	0.049	0.667	<0.001
**n-3 : males**	18.4 (2.97)	17.1 (2.42)	−1.11 (−2.42, 0.20)	0.096		<0.001
**n-6 : females**	13.4 (5.76)	14.9 (5.30)	+1.05 (−0.49, 2.59)	0.181	0.943	
**n-6 : males**	18.2 (2.87)	19.2 (2.83)	+0.97 (−0.34, 2.29)	0.144		

Variable and treatment	Baseline[2]	Follow-up[2]	Absolute change[3]	Time[4]	wTXbSEX[5]	bTXwSEX[6]
**VLDL size, nm**						
**n-3 : females**	46.4 (8.46)	49.3 (8.06)	+0.55 (−4.15, 5.24)	0.818	0.380	0.402
**n-3 : males**	51.3 (6.95)	48.7 (7.52)	−1.97 (−5.14, 1.20)	0.221		0.941
**n-6 : females**	51.1 (10.1)	47.5 (5.45)	−1.30 (−5.99, 3.40)	0.585	0.839	
**n-6 : males**	50.1 (9.06)	48.8 (6.12)	−1.88 (−5.05, 1.29)	0.242		
**LDL size, nm**						
**n-3 : females**	21.4 (0.59)	21.3 (0.51)	−0.050 (−0.27, 0.17)	0.652	0.951	0.032
**n-3 : males**	21.0 (0.53)	20.9 (0.33)	−0.041 (−0.23, 0.15)	0.664		0.157
**n-6 : females**	21.4 (0.80)	21.1 (0.73)	−0.27 (−0.49, −0.049)	0.017	0.472	
**n-6 : males**	20.9 (0.58)	20.8 (0.38)	−0.16 (−0.35, 0.023)	0.085		
**HDL size, nm**						
**n-3 : females**	9.74 (0.60)	9.99 (0.70)	+0.21 (0.057, 0.36)	0.007	0.034	0.014
**n-3 : males**	8.98 (0.42)	9.03 (0.46)	−0.006 (−0.13, 0.12)	0.927		0.236
**n-6 : females**	9.83 (0.72)	9.78 (0.63)	−0.006 (−0.16, 0.14)	0.934	0.387	
**n-6 : males**	9.07 (0.37)	8.95 (0.36)	−0.092 (−0.22, 0.035)	0.153		
**Lp(a) and blood lipids, serum**						
**Lp(a), mg/L**						
**n-3 : females**	174 (146)	167 (132)	−4.02 (−21.2, 13.1)	0.644	0.815	0.004
**n-3 : males**	141 (165)	145 (168)	−1.32 (−16.1, 13.5)	0.860		0.073
**n-6 : females**	170 (147)	148 (133)	−23.1 (−39.6, −6.61)	0.006	0.003	
**n-6 : males**	151 (189)	146 (166)	+8.60 (−4.29, 21.5)	0.189		
**TAGs, mmol/L**						
**n-3 : females**	1.25 (0.66)	1.05 (0.44)	−0.23 (−0.43, −0.026)	0.027	0.543	0.001
**n-3 : males**	1.56 (0.73)	1.21 (0.40)	−0.32 (−0.52, −0.12)	0.002		0.023
**n-6 : females**	1.31 (0.69)	1.31 (0.56)	+0.026 (−0.18, 0.23)	0.798	0.465	
**n-6 : males**	1.54 (0.66)	1.45 (0.53)	−0.080 (−0.28, 0.12)	0.435		
**NEFAs, mmol/L**						
**n-3 : females**	0.62 (0.30)	0.62 (0.22)	+0.030 (−0.12, 0.18)	0.693	0.153	0.969
**n-3 : males**	0.56 (0.24)	0.40 (0.19)	−0.10 (−0.20, 0.001)	0.051		0.362
**n-6 : females**	0.56 (0.22)	0.62 (0.32)	+0.033 (−0.12, 0.18)	0.662	0.295	
**n-6 : males**	0.44 (0.22)	0.44 (0.18)	−0.063 (−0.16, 0.039)	0.223		
**PLs, mmol/L**						
**n-3 : females**	3.08 (0.28)	3.02 (0.27)	−0.093 (−0.24, 0.055)	0.217	0.734	0.494
**n-3 : males**	2.77 (0.38)	2.73 (0.36)	−0.060 (−0.19, 0.067)	0.352		0.944
**n-6 : females**	3.15 (0.38)	3.08 (0.37)	−0.036 (−0.18, 0.11)	0.630	0.852	
**n-6 : males**	2.79 (0.41)	2.73 (0.35)	−0.055 (−0.18, 0.072)	0.393		
**TC, mmol/L**						
**n-3 : females**	6.12 (0.72)	6.13 (0.66)	−0.013 (−0.32, 0.29)	0.936	0.830	0.053
**n-3 : males**	5.52 (0.91)	5.50 (0.89)	−0.056 (−0.32, 0.20)	0.670		0.174
**n-6 : females**	6.16 (0.87)	5.82 (0.86)	−0.32 (−0.63, −0.018)	0.038	0.686	
**n-6 : males**	5.55 (1.01)	5.31 (0.91)	−0.24 (−0.50, 0.018)	0.068		
**FC, mmol/L**						
**n-3 : females**	1.70 (0.22)	1.70 (0.18)	−0.007 (−0.10, 0.085)	0.878	0.809	0.262
**n-3 : males**	1.55 (0.25)	1.54 (0.26)	−0.022 (−0.10, 0.057)	0.580		0.606
**n-6 : females**	1.72 (0.25)	1.65 (0.22)	−0.065 (−0.16, 0.027)	0.164	0.738	
**n-6 : males**	1.56 (0.30)	1.52 (0.26)	−0.045 (−0.12, 0.034)	0.262		
**LDL-C, mmol/L**						
**n-3 : females**	4.12 (0.77)	4.11 (0.65)	+0.016 (−0.25, 0.28)	0.908	0.756	0.015
**n-3 : males**	3.89 (0.79)	3.88 (0.84)	−0.039 (−0.27, 0.19)	0.731		0.183
**n-6 : females**	4.07 (0.83)	3.77 (0.72)	−0.33 (−0.59, −0.062)	0.016	0.463	
**n-6 : males**	3.90 (0.91)	3.72 (0.83)	−0.20 (−0.42, 0.028)	0.085		
**HDL-C, mmol/L**						
**n-3 : females**	1.76 (0.51)	1.84 (0.50)	+0.053 (−0.057, 0.16)	0.341	0.821	0.740
**n-3 : males**	1.22 (0.27)	1.30 (0.26)	+0.068 (−0.005, 0.14)	0.069		0.171
**n-6 : females**	1.82 (0.62)	1.86 (0.58)	+0.072 (−0.038, 0.18)	0.198	0.465	
**n-6 : males**	1.23 (0.26)	1.25 (0.29)	+0.023 (−0.051, 0.096)	0.540		
**Non-HDL-C, mmol/L**						
**n-3 : females**	4.36 (0.87)	4.29 (0.69)	−0.066 (−0.35, 0.22)	0.644	0.751	0.022
**n-3 : males**	4.30 (0.94)	4.20 (0.94)	−0.12 (−0.36, 0.11)	0.303		0.248
**n-6 : females**	4.34 (0.92)	3.96 (0.79)	−0.40 (−0.68, −0.12)	0.006	0.481	
**n-6 : males**	4.32 (1.04)	4.05 (0.90)	−0.26 (−0.50, −0.027)	0.029		
**TRL-C, mmol/L**						
**n-3 : females**	0.24 (0.26)	0.19 (0.17)	−0.079 (−0.15, −0.010)	0.026	0.883	0.703
**n-3 : males**	0.41 (0.25)	0.32 (0.20)	−0.085 (−0.14, −0.027)	0.005		0.462
**n-6 : females**	0.29 (0.27)	0.20 (0.21)	−0.066 (−0.15, 0.018)	0.124	0.977	
**n-6 : males**	0.41 (0.22)	0.34 (0.17)	−0.064 (−0.14, 0.007)	0.078		

Variable and treatment	Baseline[2]	Follow-up[2]	Absolute change[3]	Time[4]	wTXbSEX[5]	bTXwSEX[6]
**TAG/HDL-C ratio**						
**n-3 : females**	0.85 (0.64)	0.66 (0.41)	−0.10 (−0.28, 0.077)	0.261	0.057	0.105
**n-3 : males**	1.37 (0.77)	0.98 (0.39)	−0.35 (−0.54, −0.17)	<0.001		<0.001
**n-6 : females**	0.89 (0.69)	0.84 (0.56)	−0.020 (−0.21, 0.17)	0.833	0.731	
**n-6 : males**	1.36 (0.81)	1.22 (0.53)	−0.066 (−0.25, 0.12)	0.476		
**TC/HDL-C ratio**						
**n-3 : females**	3.75 (1.11)	3.55 (0.94)	−0.19 (−0.48, 0.10)	0.200	0.490	0.197
**n-3 : males**	4.73 (1.30)	4.38 (1.10)	−0.33 (−0.58, −0.076)	0.011		0.903
**n-6 : females**	3.72 (1.19)	3.37 (0.93)	−0.37 (−0.66, −0.076)	0.014	0.768	
**n-6 : males**	4.70 (1.36)	4.39 (1.10)	−0.31 (−0.56, −0.062)	0.015		
**Apolipoproteins, serum**						
**ApoB, g/L**						
**n-3 : females**	1.12 (0.20)	1.12 (0.14)	+0.002 (−0.057, 0.061)	0.942	0.880	0.021
**n-3 : males**	1.10 (0.20)	1.11 (0.22)	−0.004 (−0.054, 0.047)	0.882		0.043
**n-6 : females**	1.12 (0.20)	1.05 (0.18)	−0.072 (−0.13, −0.013)	0.018	0.742	
**n-6 : males**	1.12 (0.23)	1.05 (0.21)	−0.059 (−0.11, −0.009)	0.022		
**ApoA-I, g/L**						
**n-3 : females**	1.65 (0.23)	1.61 (0.19)	−0.059 (−0.14, 0.018)	0.130	0.151	0.050
**n-3 : males**	1.37 (0.18)	1.39 (0.18)	+0.010 (−0.046, 0.066)	0.721		0.826
**n-6 : females**	1.69 (0.30)	1.69 (0.28)	+0.022 (−0.055, 0.099)	0.575	0.912	
**n-6 : males**	1.38 (0.17)	1.40 (0.20)	+0.017 (−0.040, 0.073)	0.561		
**ApoA-II, g/L**						
**n-3 : females**	0.47 (0.035)	0.44 (0.061)	−0.034 (−0.057, −0.010)	0.005	0.291	0.004
**n-3 : males**	0.47 (0.054)	0.45 (0.050)	−0.017 (−0.037, 0.002)	0.084		0.022
**n-6 : females**	0.48 (0.036)	0.48 (0.057)	+0.002 (−0.021, 0.025)	0.887	0.767	
**n-6 : males**	0.46 (0.048)	0.47 (0.057)	+0.006 (−0.013, 0.026)	0.533		
**ApoC-II, g/L**						
**n-3 : females**	0.22 (0.047)	0.20 (0.044)	−0.022 (−0.041, −0.003)	0.021	0.786	0.155
**n-3 : males**	0.22 (0.070)	0.19 (0.050)	−0.026 (−0.042, −0.010)	0.002		0.078
**n-6 : females**	0.22 (0.047)	0.22 (0.046)	−0.006 (−0.025, 0.013)	0.545	0.847	
**n-6 : males**	0.21 (0.074)	0.20 (0.061)	−0.008 (−0.024, 0.008)	0.315		
**ApoC-III, g/L**						
**n-3 : females**	0.32 (0.071)	0.30 (0.074)	−0.029 (−0.063, 0.006)	0.101	0.899	0.066
**n-3 : males**	0.29 (0.10)	0.26 (0.072)	−0.026 (−0.055, 0.003)	0.083		0.150
**n-6 : females**	0.34 (0.075)	0.34 (0.073)	+0.007 (−0.028, 0.041)	0.693	0.694	
**n-6 : males**	0.28 (0.084)	0.28 (0.090)	−0.002 (−0.031, 0.027)	0.887		
**ApoE, g/L**						
**n-3 : females**	0.089 (0.014)	0.095 (0.017)	+0.004 (−0.004, 0.012)	0.366	0.714	0.158
**n-3 : males**	0.095 (0.026)	0.097 (0.024)	+0.002 (−0.005, 0.008)	0.590		0.073
**n-6 : females**	0.094 (0.018)	0.090 (0.018)	−0.002 (−0.010, 0.007)	0.713	0.706	
**n-6 : males**	0.095 (0.028)	0.091 (0.021)	−0.003 (−0.010, 0.003)	0.291		
**ApoB/ApoA-I ratio**						
**n-3 : females**	0.69 (0.18)	0.71 (0.15)	+0.019 (−0.025, 0.064)	0.391	0.356	0.002
**n-3 : males**	0.82 (0.19)	0.81 (0.20)	−0.008 (−0.046, 0.030)	0.676		0.037
**n-6 : females**	0.69 (0.18)	0.63 (0.14)	−0.057 (−0.10, −0.012)	0.013	0.830	
**n-6 : males**	0.82 (0.20)	0.77 (0.20)	−0.050 (−0.088, −0.012)	0.010		
**ApoC-II/ApoC-III ratio**						
**n-3 : females**	0.69 (0.078)	0.67 (0.091)	−0.005 (−0.041, 0.031)	0.791	0.782	0.376
**n-3 : males**	0.76 (0.14)	0.75 (0.18)	−0.011 (−0.042, 0.019)	0.462		0.805
**n-6 : females**	0.66 (0.068)	0.65 (0.11)	−0.026 (−0.062, 0.010)	0.154	0.689	
**n-6 : males**	0.76 (0.14)	0.74 (0.15)	−0.017 (−0.047, 0.014)	0.289		

[1] Fasting blood levels were analyzed with cLMMs adjusted for the main effects of period and subject-averaged baselines. Total VLDLs and large VLDLs also include chylomicrons if present. Abbreviations: Apo, apolipoprotein; bTXwSEX, between-treatment within-sex; FC, free cholesterol; HDLs, high-density lipoprotein particles; HDL-C, HDL cholesterol; IDLs, intermediate-density lipoprotein particles; LDLs, low-density lipoprotein particles; LDL-C, LDL cholesterol; cLMM, constrained linear mixed-effects model; Lp(a), lipoprotein (a); n-3, omega-3 PUFAs; n-6, omega-6 PUFAs; NEFAs, non-esterified fatty acids; non-HDL-C, non-HDL cholesterol; PLs, phospholipids; PUFAs, polyunsaturated fatty acids; TAGs, triacylglycerols; TC, total cholesterol; TRL-C, TAG-rich lipoprotein cholesterol; VLDLs, very-low-density lipoprotein particles; wTXbSEX, within-treatment between-sex.
[2] Values are arithmetic means (SDs) of fasting blood levels at baseline and follow-up within treatments and sexes.
[3] Absolute model-adjusted mean change scores (95% CIs) from baseline to follow-up.
[4] P-values for absolute changes from baseline to follow-up within treatments and sexes (time effects).
[5] P-values for absolute changes from baseline to follow-up within treatments and between sexes (sex differences in time effects). The first and second values refer to between-sex differences after the n-3 and n-6 interventions, respectively.
[6] P-values for absolute changes from baseline to follow-up between treatments and within sexes (group differences in time effects within each stratum of sex). The first and second values refer to between-treatment differences in females and males, respectively.

frontiers

SUPPLEMENTARY TABLE 11 Relative sex-specific differences at pre-treatment baseline in anthropometric measures, circulating markers of glycemic control / insulin sensitivity and liver function, and hormone and ketone levels[1]

Variable and treatment	Females[2]	Males[2]	Relative difference[3]	P-value[4]
**Glycemic control / insulin sensitivity, serum**				
**Glucose, mmol/L**	5.01 (4.69, 5.34)	5.41 (4.93, 5.94)	−7.46 (−12.2, −2.44)	0.007
**HbA1c, %**	5.48 (5.23, 5.75)	5.46 (5.20, 5.74)	0.34 (−2.73, 3.49)	0.834
**Insulin, mU/L**	6.94 (3.70, 13.0)	10.3 (5.91, 17.9)	−32.6 (−53.8, −1.65)	0.048
**INCP, nmol/L**	0.57 (0.39, 0.82)	0.79 (0.56, 1.12)	−28.1 (−42.9, −9.49)	0.008
**HOMA2-IR**	1.25 (0.86, 1.83)	1.78 (1.25, 2.54)	−29.8 (−44.5, −11.1)	0.006
**HOMA2-%S**	79.9 (54.6, 117)	56.1 (39.3, 80.1)	42.4 (12.5, 80.3)	0.006
**HOMA2-%B**	111 (87.6, 140)	120 (93.8, 155)	−7.95 (−21.3, 7.70)	0.308
**QUICKI**	0.16 (0.14, 0.17)	0.14 (0.13, 0.16)	7.46 (1.38, 13.9)	0.021
**rQUICKI**	0.17 (0.15, 0.20)	0.17 (0.14, 0.20)	2.18 (−7.71, 13.1)	0.680
**LP-IR**	21.8 (7.85, 60.3)	53.1 (35.2, 80.0)	−59.0 (−75.8, −30.5)	0.002
**Anthropometrics**				
**Body weight, kg**	79.3 (66.7, 94.4)	95.3 (84.0, 108)	−16.8 (−24.3, −8.53)	0.001
**BMI, kg/m^2**	28.2 (24.2, 32.7)	29.5 (26.0, 33.5)	−4.68 (−12.6, 3.93)	0.284
**Body fat mass, kg**	30.5 (22.5, 41.4)	28.6 (20.6, 39.5)	6.88 (−12.9, 31.1)	0.528
**Body fat, %**	38.5 (33.1, 44.8)	30.1 (24.0, 37.7)	27.9 (13.4, 44.2)	<0.001
**Waist circumference, cm**	99.3 (89.1, 111)	106 (98.1, 115)	−6.59 (−12.0, −0.86)	0.031
**Waist/hip ratio**	0.92 (0.86, 0.98)	0.98 (0.94, 1.02)	−5.79 (−9.01, −2.45)	0.002
**Waist/height ratio**	0.59 (0.54, 0.65)	0.59 (0.54, 0.64)	0.26 (−5.33, 6.17)	0.930
**Visceral fat area, cm^2**	124 (83.9, 185)	175 (132, 233)	−28.9 (−42.6, −12.1)	0.003
**Fat-free mass, kg**	48.2 (43.2, 53.8)	65.3 (59.5, 71.7)	−26.3 (−30.9, −21.3)	<0.001
**Liver function markers, serum**				
**ALAT, U/L**	20.9 (14.8, 29.6)	33.0 (24.1, 45.3)	−36.6 (−48.6, −21.8)	<0.001
**Albumin, g/L**	43.0 (40.7, 45.5)	45.0 (43.0, 47.0)	−4.36 (−7.31, −1.30)	0.008
**ALP, U/L**	67.8 (49.8, 92.4)	69.6 (54.8, 88.5)	−2.62 (−18.2, 16.0)	0.767
**ASAT, U/L**	21.3 (17.4, 26.0)	26.1 (19.0, 35.9)	−18.5 (−30.7, −4.00)	0.019
**Bile acids, µmol/L**	1.86 (1.08, 3.20)	2.86 (1.48, 5.55)	−35.0 (−56.3, −3.38)	0.040
**Bilirubin, µmol/L**	5.78 (4.17, 8.01)	8.98 (5.80, 13.9)	−35.6 (−50.0, −17.1)	0.002
**CK, U/L**	64.9 (45.4, 92.7)	120 (70.2, 204)	−45.8 (−59.1, −28.0)	<0.001
**GGT, U/L[5]**	17.0 (11.0, 26.2)	32.8 (17.7, 60.7)	−48.2 (−63.9, −25.7)	0.001
**LD, U/L**	157 (140, 177)	157 (140, 176)	−0.14 (−7.22, 7.47)	0.970
**Additional hormones, serum**				
**Estrogen, pmol/L[5]**	113 (47.2, 272)	116 (92.6, 146)	−2.59 (−40.0, 58.2)	0.916
**Testosterone, nmol/L**	0.74 (0.42, 1.32)	13.9 (10.4, 18.6)	−94.7 (−96.1, −92.7)	<0.001
**SHBG, nmol/L**	54.3 (32.4, 91.2)	34.8 (20.9, 58.0)	56.2 (11.4, 119)	0.014
**FAI**	1.37 (0.86, 2.17)	40.0 (28.0, 57.2)	−96.6 (−97.4, −95.5)	<0.001
**FSH, IU/L**	25.4 (7.50, 85.7)	4.10 (2.01, 8.34)	519 (212, 1127)	<0.001
**TSH, mU/L**	1.45 (0.96, 2.18)	1.54 (0.91, 2.60)	−5.79 (−31.0, 28.6)	0.709
**FT4, pmol/L**	15.0 (13.0, 17.3)	16.8 (14.8, 19.1)	−10.4 (−17.8, −2.32)	0.017
**IGF-1, nmol/L**	17.3 (13.4, 22.2)	20.9 (14.9, 29.4)	−17.4 (−32.2, 0.58)	0.065
**Vitamin D$_3$, nmol/L**	74.6 (61.1, 91.2)	74.0 (58.2, 94.0)	0.93 (−12.6, 16.5)	0.900
**Ketone bodies, serum**				
**3-hydroxybutyrate, µmol/L**	72.3 (32.0, 163)	53.7 (25.4, 113)	34.6 (−18.0, 121)	0.247
**Acetoacetate, µmol/L**	36.2 (19.6, 67.0)	34.6 (18.1, 65.9)	4.63 (−30.1, 56.7)	0.827

[1] Pre-treatment anthropometric measurements and fasting serum levels of circulating markers were analyzed with GLS models adjusted for heterogeneity of variance by using the *gls* function in the R package *nlme* v3.1-157. Data were transformed by the natural logarithm before the analyses. Abbreviations: ALAT, alanine aminotransferase; ALP, alkaline phosphatase; ASAT, aspartate aminotransferase; CK, creatine kinase; FAI, free androgen index; FSH, follicle-stimulating hormone; FT4, free thyroxine; GLS, generalized least squares; GGT, γ-glutamyl transpeptidase; HbA1c, glycated hemoglobin; HOMA2-IR, homeostasis model assessment of insulin resistance index 2 (computer model); HOMA2-%B, homeostasis model assessment of β-cell function index 2 (computer model); HOMA2-%S, homeostasis model assessment of insulin sensitivity index 2 (computer model); IGF-1, insulin-like growth factor-1; INCP, insulin C-peptide; LD, lactate dehydrogenase; LP-IR, lipoprotein-based insulin resistance index; QUICKI, quantitative insulin sensitivity check index; rQUICKI, revised QUICKI; SHBG, sex hormone-binding globulin; TSH, thyroid-stimulating hormone.

[2] Values are geometric means (1 SD ranges) of anthropometric variables and fasting serum levels measured at the first baseline visit before any intervention.

[3] Relative model-adjusted differences (95% CIs) between sexes (females vs. males) as percentages calculated from the model estimates: % = $(\exp^{\text{estimate}} - 1) \times 100$.

[4] P-values for relative between-sex differences.
[5] Two outliers were excluded from the analyses of GGT and estrogen (different cases).

frontiers

SUPPLEMENTARY TABLE 12 Absolute sex-specific differences at pre-treatment baseline in anthropometric measures, circulating markers of glycemic control / insulin sensitivity and liver function, and hormone levels[1]

Variable and treatment	Females[2]	Males[2]	Absolute difference[3]	P-value[4]
**Glycemic control/insulin sensitivity, serum**				
**Glucose, mmol/L**	5.02 (0.33)	5.43 (0.50)	−0.42 (−0.68, −0.15)	0.004
**HbA1c, %**	5.49 (0.26)	5.47 (0.27)	0.018 (−0.15, 0.19)	0.837
**Insulin, mU/L**	8.41 (5.97)	12.0 (7.50)	−3.59 (−8.04, 0.86)	0.123
**INCP, nmol/L**	0.61 (0.24)	0.84 (0.31)	−0.23 (−0.41, −0.049)	0.018
**HOMA2−IR**	1.34 (0.53)	1.90 (0.73)	−0.56 (−0.98, −0.13)	0.014
**HOMA2−%S**	85.3 (30.2)	59.4 (19.6)	25.9 (8.94, 42.8)	0.005
**HOMA2−%B**	114 (27.7)	124 (31.0)	−10.2 (−29.3, 8.90)	0.302
**QUICKI**	0.16 (0.016)	0.15 (0.012)	0.011 (0.002, 0.020)	0.019
**rQUICKI**	0.17 (0.029)	0.17 (0.026)	0.004 (−0.014, 0.022)	0.675
**LP−IR**	32.3 (25.2)	57.0 (20.1)	−24.7 (−38.9, −10.4)	0.002
**Anthropometrics**				
**Body weight, kg**	80.5 (15.1)	96.0 (12.8)	−15.5 (−24.3, −6.76)	0.001
**BMI, kg/m^2**	28.5 (4.50)	29.8 (3.82)	−1.30 (−3.93, 1.32)	0.336
**Body fat mass, kg**	32.0 (10.7)	30.1 (10.5)	1.90 (−4.92, 8.72)	0.588
**Body fat, %**	38.9 (5.79)	30.8 (6.85)	8.07 (3.93, 12.2)	0.001
**Waist circumference, cm**	99.9 (11.2)	107 (8.69)	−6.78 (−13.0, −0.53)	0.040
**Waist/hip ratio**	0.92 (0.057)	0.98 (0.040)	−0.056 (−0.086, −0.025)	0.001
**Waist/height ratio**	0.60 (0.060)	0.59 (0.049)	0.002 (−0.032, 0.037)	0.892
**Visceral fat area, cm^2**	134 (51.3)	183 (61.1)	−49.2 (−85.8, −12.7)	0.012
**Fat−free mass, kg**	48.4 (5.35)	65.6 (6.08)	−17.1 (−20.9, −13.4)	<0.001
**Liver function, serum**				
**ALAT, U/L**	22.2 (8.71)	34.9 (14.2)	−12.7 (−19.9, −5.46)	0.001
**Albumin, g/L**	43.1 (2.41)	45.0 (1.98)	−1.94 (−3.32, −0.56)	0.009
**ALP, U/L**	71.1 (24.2)	71.8 (20.1)	−0.65 (−14.7, 13.5)	0.929
**ASAT, U/L**	21.7 (4.38)	27.5 (9.58)	−5.79 (−10.3, −1.33)	0.015
**Bile acids, µmol/L**	2.09 (0.94)	3.48 (2.31)	−1.38 (−2.45, −0.31)	0.016
**Bilirubin, µmol/L**	6.06 (1.84)	9.78 (4.09)	−3.72 (−5.62, −1.82)	<0.001
**CK, U/L**	68.8 (24.0)	136 (70.0)	−67.3 (−98.8, −35.8)	<0.001
**GGT, U/L[5]**	18.5 (8.07)	40.7 (35.6)	−22.2 (−37.6, −6.76)	0.008
**LD, U/L**	158 (18.3)	158 (17.5)	−0.18 (−11.6, 11.2)	0.976
**Additional hormones, serum**				
**Estrogen, pmol/L[5]**	170 (177)	119 (26.5)	50.4 (−46.7, 147)	0.316
**Testosterone, nmol/L**	0.87 (0.52)	14.5 (4.20)	−13.6 (−15.4, −11.8)	<0.001
**SHBG, nmol/L**	61.5 (31.3)	38.6 (16.5)	22.8 (5.55, 40.1)	0.014
**FAI**	1.50 (0.66)	42.8 (19.2)	−41.3 (−49.3, −33.3)	<0.001
**FSH, IU/L**	41.5 (33.0)	5.13 (3.56)	36.4 (19.6, 53.1)	<0.001
**TSH, mU/L**	1.58 (0.74)	1.74 (0.85)	−0.16 (−0.68, 0.36)	0.545
**FT4, pmol/L**	15.2 (2.19)	16.9 (2.23)	−1.74 (−3.16, −0.31)	0.022
**IGF-1, nmol/L**	17.8 (4.74)	22.0 (6.69)	−4.19 (−8.02, −0.35)	0.039
**Vitamin D$_3$, nmol/L**	76.0 (14.8)	76.0 (18.6)	0.009 (−10.9, 11.0)	0.999
**Ketone bodies, serum**				
**3-hydroxybutyrate, µmol/L**	98.3 (80.8)	70.5 (58.0)	27.8 (−15.7, 71.3)	0.219
**Acetoacetate, µmol/L**	43.4 (28.3)	42.5 (30.3)	0.86 (−18.0, 19.7)	0.929

[1] Pre-treatment anthropometric measurements and fasting serum levels of circulating markers were analyzed with GLS models adjusted for heterogeneity of variance by using the *gls* function in the R package *nlme* v3.1-157. Abbreviations: ALAT, alanine aminotransferase; ALP, alkaline phosphatase; ASAT, aspartate aminotransferase; CK, creatine kinase; FAI, free androgen index; FSH, follicle-stimulating hormone; FT4, free thyroxine; GLS, generalized least squares; GGT, γ-glutamyl transpeptidase; HbA1c, glycated hemoglobin; HOMA2-IR, homeostasis model assessment of insulin resistance index 2 (computer model); HOMA2-%B, homeostasis model assessment of β-cell function index 2 (computer model); HOMA2-%S, homeostasis model assessment of insulin sensitivity index 2 (computer model); IGF-1, insulin-like growth factor-1; INCP, insulin C-peptide; LD, lactate dehydrogenase; LP-IR, lipoprotein-based insulin resistance index; QUICKI, quantitative insulin sensitivity check index; rQUICKI, revised QUICKI; SHBG, sex hormone-binding globulin; TSH, thyroid-stimulating hormone.
[2] Values are arithmetic means (SDs) of anthropometric variables and fasting serum levels measured at the first baseline visit before any intervention.
[3] Absolute model-adjusted differences between sexes (females vs. males) at baseline.
[4] P-values for absolute between-sex differences.
[5] Two outliers were excluded from the analyses of GGT and estrogen (different cases).

SUPPLEMENTARY TABLE 13 Sex-specific responses in relative changes for anthropometric measures, circulating markers of glycemic control / insulin sensitivity and liver function, and hormone and ketone levels after seven wk of supplementation with n-3 or n-6 PUFAs[1]

Variable and treatment	Baseline[2]	Follow-up[2]	Relative change[3]	Time[4]	wTXbSEX[5]	bTXwSEX[6]
**Glycemic control/insulin sensitivity, serum**						
**Glucose, mmol/L**						
**n-3 : females**	5.18 (4.79, 5.60)	5.08 (4.84, 5.32)	−2.07 (−5.94, 1.95)	0.305	0.029	0.101
**n-3 : males**	5.54 (5.13, 5.99)	5.75 (5.25, 6.29)	+3.90 (0.40, 7.52)	0.029		0.129
**n-6 : females**	5.19 (4.76, 5.66)	5.24 (4.75, 5.77)	+1.07 (−2.92, 5.22)	0.603	0.918	
**n-6 : males**	5.50 (4.93, 6.13)	5.60 (5.15, 6.10)	+1.34 (−2.07, 4.88)	0.441		
**HbA1c, %**						
**n-3 : females**	5.47 (5.19, 5.78)	5.51 (5.30, 5.72)	+0.62 (−0.74, 2.01)	0.370	0.800	0.621
**n-3 : males**	5.45 (5.16, 5.75)	5.48 (5.22, 5.75)	+0.39 (−0.74, 1.54)	0.495		0.679
**n-6 : females**	5.48 (5.22, 5.74)	5.49 (5.25, 5.74)	+0.26 (−1.10, 1.64)	0.708	0.916	
**n-6 : males**	5.47 (5.22, 5.74)	5.47 (5.24, 5.71)	+0.16 (−0.97, 1.31)	0.776		
**Insulin, mU/L**						
**n-3 : females**	7.23 (3.58, 14.6)	6.18 (3.36, 11.4)	−30.5 (−42.1, −16.6)	<0.001	<0.001	<0.001
**n-3 : males**	10.1 (5.45, 18.8)	13.3 (8.51, 20.7)	+16.2 (−0.50, 35.8)	0.058		0.482
**n-6 : females**	9.43 (5.91, 15.0)	8.58 (4.58, 16.1)	−3.13 (−18.0, 14.4)	0.706	0.248	
**n-6 : males**	11.8 (6.80, 20.6)	12.6 (7.35, 21.5)	+10.1 (−4.45, 27.0)	0.181		
**INCP, nmol/L**						
**n-3 : females**	0.57 (0.37, 0.89)	0.55 (0.37, 0.80)	−11.8 (−21.2, −1.34)	0.028	0.001	0.007
**n-3 : males**	0.78 (0.54, 1.14)	0.92 (0.70, 1.20)	+12.5 (2.22, 23.9)	0.017		0.221
**n-6 : females**	0.67 (0.49, 0.90)	0.63 (0.41, 0.98)	+2.35 (−8.54, 14.6)	0.683	0.616	
**n-6 : males**	0.85 (0.61, 1.18)	0.87 (0.64, 1.17)	+6.27 (−3.45, 17.0)	0.212		
**HOMA2-IR**						
**n-3 : females**	1.28 (0.81, 2.02)	1.21 (0.82, 1.79)	−12.4 (−22.1, −1.48)	0.028	0.001	0.005
**n-3 : males**	1.78 (1.22, 2.61)	2.11 (1.61, 2.76)	+13.9 (3.08, 25.9)	0.011		0.177
**n-6 : females**	1.48 (1.08, 2.04)	1.41 (0.90, 2.23)	+2.69 (−8.69, 15.5)	0.656	0.618	
**n-6 : males**	1.92 (1.35, 2.72)	1.97 (1.45, 2.68)	+6.76 (−3.41, 18.0)	0.198		
**HOMA2-%S**						
**n-3 : females**	78.1 (49.6, 123)	82.9 (56.0, 123)	+14.2 (1.60, 28.3)	0.026	0.001	0.006
**n-3 : males**	56.1 (38.3, 82.3)	47.5 (36.2, 62.3)	−12.2 (−20.5, −3.07)	0.011		0.185
**n-6 : females**	67.5 (49.2, 92.7)	70.7 (44.9, 111)	−2.62 (−13.3, 9.42)	0.653	0.615	
**n-6 : males**	52.2 (36.7, 74.1)	50.7 (37.3, 68.9)	−6.34 (−15.2, 3.45)	0.194		
**HOMA2-%B**						
**n-3 : females**	105 (85.2, 129)	105 (83.3, 132)	−4.68 (−12.8, 4.17)	0.287	0.345	0.307
**n-3 : males**	114 (88.1, 148)	119 (92.1, 154)	+0.80 (−6.56, 8.73)	0.836		0.855
**n-6 : females**	116 (94.6, 141)	110 (88.1, 137)	−0.26 (−8.74, 9.01)	0.954	0.768	
**n-6 : males**	122 (96.9, 155)	120 (97.2, 148)	+1.49 (−5.91, 9.48)	0.699		
**QUICKI**						
**n-3 : females**	0.15 (0.14, 0.17)	0.16 (0.14, 0.18)	+4.92 (1.32, 8.64)	0.007	<0.001	0.004
**n-3 : males**	0.14 (0.13, 0.16)	0.14 (0.13, 0.15)	−3.42 (−5.53, −1.27)	0.002		0.187
**n-6 : females**	0.15 (0.14, 0.16)	0.15 (0.14, 0.17)	−0.69 (−4.09, 2.84)	0.696	0.458	
**n-6 : males**	0.14 (0.13, 0.16)	0.14 (0.13, 0.15)	−2.21 (−4.35, −0.032)	0.047		
**rQUICKI**						
**n-3 : females**	0.17 (0.14, 0.20)	0.18 (0.15, 0.20)	+4.07 (−1.98, 10.5)	0.190	0.349	0.081
**n-3 : males**	0.16 (0.14, 0.18)	0.16 (0.14, 0.19)	+0.25 (−4.74, 5.51)	0.922		0.725
**n-6 : females**	0.16 (0.14, 0.19)	0.17 (0.14, 0.20)	−0.91 (−6.67, 5.21)	0.764	0.934	
**n-6 : males**	0.17 (0.14, 0.19)	0.16 (0.14, 0.19)	−0.58 (−5.54, 4.63)	0.822		
**LP-IR**						
**n-3 : females**	18.6 (6.67, 52.0)	20.5 (8.64, 48.7)	−7.59 (−30.0, 21.9)	0.573	0.587	0.040
**n-3 : males**	54.7 (38.0, 78.6)	44.0 (30.2, 64.1)	−15.3 (−27.1, −1.52)	0.031		<0.001
**n-6 : females**	26.5 (9.78, 71.8)	27.1 (12.8, 57.4)	+21.9 (−7.59, 60.8)	0.159	0.296	
**n-6 : males**	50.1 (34.0, 73.8)	53.6 (39.5, 72.7)	+3.17 (−11.2, 19.9)	0.681		
**Anthropometrics**						
**Body weight, kg**						
**n-3 : females**	79.2 (66.3, 94.7)	79.1 (66.7, 93.8)	−0.41 (−1.33, 0.52)	0.382	0.089	0.004
**n-3 : males**	96.0 (84.8, 109)	96.1 (84.5, 109)	+0.64 (−0.14, 1.44)	0.109		0.474
**n-6 : females**	79.6 (67.1, 94.5)	80.2 (67.6, 95.2)	+0.97 (0.039, 1.91)	0.041	0.955	
**n-6 : males**	95.6 (83.8, 109)	96.5 (85.1, 109)	+0.94 (0.14, 1.73)	0.021		
**BMI, kg/m^2**						
**n-3 : females**	28.1 (24.1, 32.8)	28.1 (24.3, 32.5)	−0.41 (−1.33, 0.52)	0.381	0.089	0.004
**n-3 : males**	29.7 (26.3, 33.6)	29.9 (26.3, 33.9)	+0.64 (−0.14, 1.44)	0.108		0.474
**n-6 : females**	28.3 (24.4, 32.7)	28.5 (24.6, 33.0)	+0.97 (0.041, 1.91)	0.041	0.954	
**n-6 : males**	29.7 (26.0, 33.9)	30.0 (26.4, 34.0)	+0.94 (0.15, 1.73)	0.021		

Variable and treatment	Baseline[2]	Follow-up[2]	Relative change[3]	Time[4]	wTXbSEX[5]	bTXwSEX[6]
**Body fat mass, kg**						
**n-3 : females**	30.4 (22.2, 41.7)	30.5 (22.8, 40.9)	−0.76 (−3.48, 2.05)	0.590	0.065	0.066
**n-3 : males**	28.6 (20.7, 39.6)	29.6 (21.7, 40.3)	+2.62 (0.19, 5.12)	0.035		0.847
**n-6 : females**	30.8 (23.2, 41.0)	30.9 (22.8, 41.8)	+2.10 (−0.71, 4.98)	0.142	0.884	
**n-6 : males**	28.9 (20.7, 40.3)	29.8 (21.5, 41.2)	+2.37 (−0.062, 4.85)	0.056		
**Body fat, %**						
**n-3 : females**	38.4 (32.8, 45.0)	38.6 (33.5, 44.5)	−0.56 (−2.89, 1.82)	0.637	0.129	0.305
**n-3 : males**	30.0 (23.9, 37.5)	30.7 (25.0, 37.8)	+1.78 (−0.28, 3.88)	0.090		0.703
**n-6 : females**	38.7 (33.8, 44.2)	38.5 (32.9, 45.1)	+0.77 (−1.59, 3.19)	0.521	0.708	
**n-6 : males**	30.2 (24.0, 38.0)	30.9 (24.7, 38.5)	+1.35 (−0.70, 3.44)	0.196		
**Waist circumference, cm**						
**n-3 : females**	98.8 (88.0, 111)	96.3 (87.3, 106)	−2.54 (−8.37, 3.66)	0.410	0.385	0.291
**n-3 : males**	106 (98.2, 115)	106 (97.8, 115)	+0.16 (−0.67, 1.00)	0.698		0.457
**n-6 : females**	99.0 (89.2, 110)	99.6 (89.4, 111)	+0.68 (−0.16, 1.54)	0.114	0.679	
**n-6 : males**	106 (97.6, 115)	106 (98.5, 115)	+0.45 (−0.27, 1.18)	0.221		
**Waist/hip ratio**						
**n-3 : females**	0.92 (0.86, 0.98)	0.90 (0.82, 0.99)	−1.96 (−7.36, 3.77)	0.492	0.416	0.443
**n-3 : males**	0.97 (0.94, 1.01)	0.98 (0.94, 1.02)	+0.39 (−0.49, 1.28)	0.386		0.493
**n-6 : females**	0.91 (0.86, 0.98)	0.92 (0.86, 0.98)	+0.19 (−0.52, 0.90)	0.600	0.907	
**n-6 : males**	0.97 (0.94, 1.01)	0.98 (0.94, 1.01)	+0.13 (−0.58, 0.84)	0.724		
**Waist/height ratio**						
**n-3 : females**	0.59 (0.53, 0.65)	0.57 (0.52, 0.63)	−2.54 (−8.42, 3.71)	0.414	0.389	0.294
**n-3 : males**	0.59 (0.54, 0.64)	0.59 (0.54, 0.64)	+0.17 (−0.67, 1.01)	0.693		0.456
**n-6 : females**	0.59 (0.54, 0.65)	0.59 (0.54, 0.65)	+0.68 (−0.14, 1.51)	0.103	0.679	
**n-6 : males**	0.59 (0.54, 0.64)	0.59 (0.55, 0.64)	+0.45 (−0.27, 1.18)	0.220		
**Visceral fat area, cm^2**						
**n-3 : females**	130 (87.4, 194)	127 (86.3, 187)	−3.07 (−13.2, 8.29)	0.578	0.344	0.899
**n-3 : males**	176 (135, 228)	183 (142, 235)	+2.56 (−1.49, 6.79)	0.217		0.687
**n-6 : females**	132 (90.9, 191)	128 (86.3, 191)	−2.10 (−12.4, 9.37)	0.704	0.514	
**n-6 : males**	181 (137, 240)	181 (135, 244)	+1.78 (−2.24, 5.98)	0.387		
**Fat-free mass, kg**						
**n-3 : females**	48.2 (43.1, 54.0)	48.1 (43.0, 53.8)	+0.041 (−1.16, 1.25)	0.946	0.922	0.262
**n-3 : males**	65.9 (60.2, 72.1)	65.7 (60.0, 72.0)	+0.12 (−0.91, 1.16)	0.819		0.573
**n-6 : females**	48.3 (43.1, 54.1)	48.7 (43.4, 54.7)	+0.79 (−0.42, 2.01)	0.199	0.659	
**n-6 : males**	65.7 (59.8, 72.3)	65.8 (60.3, 71.7)	+0.44 (−0.59, 1.48)	0.403		
**Liver function markers, serum**						
**ALAT, U/L**						
**n-3 : females**	19.5 (12.1, 31.4)	21.0 (12.5, 35.5)	+8.92 (−4.51, 24.2)	0.201	0.973	0.951
**n-3 : males**	31.1 (21.4, 45.4)	35.8 (24.4, 52.4)	+8.59 (−3.57, 22.3)	0.172		0.499
**n-6 : females**	19.2 (12.5, 29.5)	21.1 (12.9, 34.4)	+9.27 (−4.21, 24.6)	0.185	0.597	
**n-6 : males**	34.7 (23.7, 50.8)	34.3 (24.4, 48.3)	+4.20 (−7.46, 17.3)	0.493		
**Albumin, g/L**						
**n-3 : females**	43.3 (41.1, 45.6)	43.5 (40.5, 46.6)	+0.70 (−2.01, 3.48)	0.614	0.455	0.697
**n-3 : males**	45.1 (43.4, 46.8)	44.8 (42.5, 47.1)	−0.66 (−2.93, 1.67)	0.574		0.939
**n-6 : females**	43.0 (40.8, 45.4)	43.2 (40.7, 45.9)	+0.18 (−2.52, 2.94)	0.899	0.611	
**n-6 : males**	45.0 (42.9, 47.1)	44.7 (42.6, 46.9)	−0.74 (−3.02, 1.58)	0.525		
**ALP, U/L**						
**n-3 : females**	66.3 (49.3, 89.2)	63.4 (45.7, 87.8)	−4.74 (−9.70, 0.49)	0.075	0.572	0.008
**n-3 : males**	66.6 (53.0, 83.6)	63.9 (51.8, 78.8)	−6.63 (−10.8, −2.28)	0.004		0.002
**n-6 : females**	66.7 (47.8, 93.1)	69.1 (51.0, 93.6)	+3.83 (−1.58, 9.53)	0.167	0.547	
**n-6 : males**	70.3 (55.9, 88.5)	69.5 (55.6, 86.9)	+1.63 (−2.90, 6.37)	0.484		
**ASAT, U/L**						
**n-3 : females**	21.4 (16.8, 27.2)	22.3 (17.1, 29.0)	+3.75 (−6.30, 14.9)	0.476	0.934	0.707
**n-3 : males**	26.5 (18.8, 37.3)	27.9 (21.5, 36.2)	+4.33 (−4.33, 13.8)	0.334		0.158
**n-6 : females**	21.6 (17.8, 26.1)	22.7 (17.6, 29.2)	+5.63 (−4.60, 17.0)	0.289	0.299	
**n-6 : males**	26.5 (20.1, 35.1)	26.3 (20.7, 33.4)	−1.54 (−9.72, 7.37)	0.723		
**Bile acids, µmol/L**						
**n-3 : females**	1.91 (1.06, 3.44)	2.10 (1.17, 3.76)	+0.79 (−26.1, 37.5)	0.960	0.557	0.738
**n-3 : males**	3.06 (1.46, 6.44)	3.33 (2.02, 5.49)	+13.8 (−12.7, 48.2)	0.336		0.654
**n-6 : females**	2.27 (1.33, 3.87)	2.19 (1.22, 3.93)	+5.25 (−22.8, 43.5)	0.744	0.537	
**n-6 : males**	2.79 (1.65, 4.72)	3.49 (1.91, 6.39)	+19.5 (−8.25, 55.7)	0.184		
**Bilirubin, µmol/L**						
**n-3 : females**	5.49 (3.23, 9.32)	6.16 (4.50, 8.45)	+14.8 (−5.14, 38.9)	0.155	0.215	0.144
**n-3 : males**	9.40 (6.04, 14.6)	8.69 (5.94, 12.7)	−1.94 (−16.6, 15.3)	0.811		0.141
**n-6 : females**	5.25 (4.02, 6.87)	5.32 (3.66, 7.72)	−0.94 (−18.1, 19.8)	0.922	0.281	
**n-6 : males**	8.32 (5.28, 13.1)	7.66 (5.33, 11.0)	−13.6 (−26.5, 1.60)	0.077		

Variable and treatment	Baseline[2]	Follow-up[2]	Relative change[3]	Time[4]	wTXbSEX[5]	bTXwSEX[6]
**CK, U/L**						
n-3 : females	71.4 (48.9, 104)	70.4 (49.5, 100)	−0.19 (−18.9, 22.8)	0.985	0.610	0.369
n-3 : males	136 (82.1, 224)	118 (76.3, 181)	−6.96 (−22.0, 11.0)	0.420		0.947
n-6 : females	69.7 (44.7, 109)	76.3 (43.7, 133)	+8.07 (−12.1, 32.9)	0.459	0.262	
n-6 : males	118 (71.0, 195)	117 (80.0, 171)	−7.42 (−22.4, 10.5)	0.389		
**GGT, U/L[7]**						
n-3 : females	17.1 (11.3, 25.8)	16.0 (10.7, 24.0)	−8.34 (−17.7, 2.10)	0.112	0.103	0.244
n-3 : males	33.6 (18.6, 60.8)	35.1 (19.7, 62.5)	+3.06 (−5.90, 12.9)	0.512		0.978
n-6 : females	17.6 (10.9, 28.3)	17.0 (9.94, 29.0)	−2.29 (−14.3, 11.4)	0.727	0.529	
n-6 : males	34.9 (18.3, 66.7)	35.7 (19.7, 64.8)	+3.20 (−7.64, 15.3)	0.575		
**LD, U/L**						
n-3 : females	154 (134, 178)	155 (138, 173)	+1.86 (−3.72, 7.77)	0.518	0.798	0.823
n-3 : males	153 (135, 173)	153 (138, 171)	+0.89 (−3.83, 5.84)	0.715		0.982
n-6 : females	150 (135, 166)	156 (139, 176)	+2.67 (−2.96, 8.63)	0.356	0.626	
n-6 : males	151 (135, 169)	153 (133, 176)	+0.82 (−3.90, 5.77)	0.736		
**Additional hormones, serum**						
**Estrogen, pmol/L[7]**						
n-3 : females	111 (54.7, 224)	90.3 (46.7, 175)	−19.0 (−30.5, −5.56)	0.008	0.173	0.667
n-3 : males	112 (83.6, 149)	110 (84.6, 142)	−8.18 (−16.6, 1.09)	0.082		0.001
n-6 : females	109 (43.2, 274)	105 (42.7, 258)	−14.3 (−33.8, 10.9)	0.237	0.120	
n-6 : males	120 (96.6, 149)	124 (94.9, 162)	+6.11 (−2.57, 15.6)	0.171		
**Testosterone, nmol/L**						
n-3 : females	0.70 (0.40, 1.24)	0.71 (0.42, 1.22)	−2.56 (−13.5, 9.79)	0.668	0.529	0.660
n-3 : males	13.8 (10.6, 17.9)	14.4 (10.6, 19.6)	+2.41 (−7.35, 13.2)	0.639		0.821
n-6 : females	0.76 (0.42, 1.37)	0.73 (0.40, 1.31)	−0.26 (−11.5, 12.4)	0.966	0.847	
n-6 : males	14.3 (10.7, 19.1)	14.2 (11.0, 18.5)	+1.26 (−8.38, 11.9)	0.804		
**SHBG, nmol/L**						
n-3 : females	51.5 (31.6, 83.9)	52.5 (34.5, 79.8)	+1.18 (−10.1, 13.8)	0.845	0.731	0.334
n-3 : males	33.8 (21.2, 54.1)	35.1 (21.4, 57.6)	+3.71 (−4.21, 12.3)	0.366		0.292
n-6 : females	51.9 (32.1, 83.8)	55.4 (34.6, 88.5)	+7.33 (−4.61, 20.8)	0.237	0.376	
n-6 : males	33.9 (20.3, 56.5)	34.1 (21.0, 55.5)	+0.71 (−6.98, 9.03)	0.861		
**FAI**						
n-3 : females	1.36 (0.82, 2.26)	1.36 (0.84, 2.18)	−3.68 (−16.5, 11.1)	0.605	0.779	0.600
n-3 : males	40.7 (28.3, 58.7)	41.0 (29.6, 56.7)	−1.25 (−10.7, 9.19)	0.804		0.672
n-6 : females	1.47 (0.93, 2.33)	1.32 (0.78, 2.23)	−7.08 (−19.5, 7.22)	0.311	0.373	
n-6 : males	42.3 (30.0, 59.7)	41.7 (28.6, 61.0)	+0.55 (−9.07, 11.2)	0.914		
**FSH, IU/L**						
n-3 : females	26.8 (8.79, 81.7)	31.9 (10.4, 97.3)	18.6 (−25.8, 89.6)	0.473	0.516	0.856
n-3 : males	4.20 (2.07, 8.52)	4.32 (2.18, 8.55)	1.43 (−5.64, 9.03)	0.697		0.191
n-6 : females	27.1 (7.62, 96.2)	30.1 (7.83, 115)	11.4 (−30.3, 78.1)	0.649	0.871	
n-6 : males	4.32 (2.12, 8.78)	4.56 (2.21, 9.41)	7.16 (−0.31, 15.2)	0.060		
**TSH, mU/L**						
n-3 : females	1.56 (1.13, 2.16)	1.69 (1.14, 2.51)	+5.59 (−12.2, 27.0)	0.561	0.999	0.863
n-3 : males	1.59 (0.95, 2.66)	1.67 (0.92, 3.04)	+5.57 (−9.50, 23.2)	0.487		0.682
n-6 : females	1.64 (0.92, 2.95)	1.66 (1.09, 2.55)	+3.96 (−13.6, 25.1)	0.678	0.879	
n-6 : males	1.58 (0.94, 2.66)	1.62 (0.97, 2.70)	+2.05 (−12.5, 19.0)	0.794		
**FT4, pmol/L**						
n-3 : females	14.9 (12.7, 17.5)	15.5 (13.0, 18.4)	+2.45 (−2.09, 7.20)	0.292	0.418	0.095
n-3 : males	16.1 (14.4, 18.0)	16.3 (14.7, 18.1)	−0.022 (−3.81, 3.92)	0.991		0.420
n-6 : females	15.3 (13.5, 17.3)	14.9 (12.7, 17.4)	−1.46 (−5.82, 3.11)	0.522	0.961	
n-6 : males	16.5 (14.5, 18.7)	16.0 (14.0, 18.4)	−1.60 (−5.34, 2.27)	0.409		
**IGF-1, nmol/L**						
n-3 : females	16.6 (12.9, 21.5)	17.6 (14.4, 21.5)	+5.31 (−10.4, 23.8)	0.528	0.569	0.005
n-3 : males	19.4 (13.8, 27.5)	20.0 (10.9, 36.9)	−1.48 (−16.4, 16.1)	0.858		0.063
n-6 : females	16.6 (13.6, 20.3)	15.6 (12.6, 19.3)	−6.28 (−20.2, 10.0)	0.425	0.641	
n-6 : males	21.7 (11.4, 41.6)	19.2 (12.5, 29.3)	−10.9 (−22.6, 2.57)	0.107		
**Vitamin D_3, nmol/L**						
n-3 : females	68.2 (52.1, 89.3)	65.8 (46.3, 93.7)	−4.47 (−13.9, 5.95)	0.383	0.229	0.641
n-3 : males	60.9 (44.8, 82.8)	54.7 (41.4, 72.3)	−11.6 (−17.9, −4.82)	0.001		0.878
n-6 : females	69.6 (55.4, 87.5)	63.9 (47.8, 85.5)	−7.25 (−16.4, 2.87)	0.153	0.417	
n-6 : males	63.7 (46.4, 87.3)	54.5 (39.8, 74.7)	−12.0 (−18.3, −5.21)	0.001		
**Ketone bodies, serum**						
**3-hydroxybutyrate, µmol/L**						
n-3 : females	67.1 (29.3, 154)	67.4 (27.1, 167)	+13.1 (−27.4, 76.3)	0.583	0.154	0.089
n-3 : males	74.9 (35.1, 160)	45.2 (25.6, 79.9)	−25.8 (−49.2, 8.25)	0.120		0.955
n-6 : females	52.9 (28.5, 98.1)	49.1 (19.5, 124)	−17.6 (−47.1, 28.4)	0.389	0.745	
n-6 : males	48.6 (21.6, 109)	45.6 (22.1, 94.1)	−25.1 (−48.7, 9.23)	0.132		

Variable and treatment	Baseline[2]	Follow-up[2]	Relative change[3]	Time[4]	wTXbSEX[5]	bTXwSEX[6]
**Acetoacetate, µmol/L**						
**n-3 : females**	35.9 (18.0, 71.4)	39.2 (19.3, 79.7)	+17.1 (−18.6, 68.6)	0.392	0.254	0.134
**n-3 : males**	48.3 (25.8, 90.5)	35.7 (19.7, 64.8)	−11.2 (−34.9, 21.1)	0.449		0.462
**n-6 : females**	31.2 (20.3, 48.0)	31.6 (15.9, 62.8)	−5.49 (−34.4, 36.1)	0.759	0.531	
**n-6 : males**	32.9 (17.0, 63.6)	32.7 (19.3, 55.3)	−18.8 (−40.5, 10.7)	0.186		

[1] Fasting blood levels were analyzed with cLMMs adjusted for the main effects of period and subject-averaged baselines. Data were transformed by the natural logarithm before the analyses. Abbreviations: ALAT, alanine aminotransferase; ALP, alkaline phosphatase; ASAT, aspartate aminotransferase; bTXwSEX, between-treatment within-sex; CK, creatine kinase; FAI, free androgen index; FSH, follicle-stimulating hormone; FT4, free thyroxine; GGT, γ-glutamyl transpeptidase; HbA1c, glycated hemoglobin; HOMA2-IR, homeostasis model assessment of insulin resistance index 2 (computer model); HOMA2-%B, homeostasis model assessment of β-cell function index 2 (computer model); HOMA2-%S, homeostasis model assessment of insulin sensitivity index 2 (computer model); IGF-1, insulin-like growth factor-1; INCP, insulin C-peptide; LD, lactate dehydrogenase; cLMM, constrained linear mixed-effects model; LP-IR, lipoprotein-based insulin resistance index; n-3, omega-3 PUFAs; n-6, omega-6 PUFAs; PUFAs, polyunsaturated fatty acids; QUICKI, quantitative insulin sensitivity check index; rQUICKI, revised QUICKI; SHBG, sex hormone-binding globulin; TSH, thyroid-stimulating hormone; wTXbSEX, within-treatment between-sex.
[2] Values are geometric means (1 SD ranges) of fasting blood levels at baseline and follow-up within treatments and sexes.
[3] Relative model-adjusted mean change scores (95% CIs) from baseline to follow-up as percentages calculated from the model estimates: $\% = (\exp^{\text{estimate}} - 1) \times 100$.
[4] P-values for relative changes from baseline to follow-up within treatments and sexes (time effects).
[5] P-values for relative changes from baseline to follow-up within treatments and between sexes (sex differences in time effects). The first and second values refer to between-sex differences after the n-3 and n-6 interventions, respectively.
[6] P-values for relative changes from baseline to follow-up between treatments and within sexes (group differences in time effects within each stratum of sex). The first and second values refer to between-treatment differences in females and males, respectively.
[7] Two influential outliers were excluded from the final analyses of GGT and estrogen (different cases).

SUPPLEMENTARY TABLE 14 Sex-specific responses in absolute changes for anthropometric measures, circulating markers of glycemic control / insulin sensitivity and liver function, and hormone and ketone levels after seven wk of supplementation with n-3 or n-6 PUFAs[1]

Variable and treatment	Baseline[2]	Follow-up[2]	Absolute change[3]	Time[4]	wTXbSEX[5]	bTXwSEX[6]
**Glycemic control/insulin sensitivity, serum**						
**Glucose, mmol/L**						
**n-3 : females**	5.19 (0.42)	5.08 (0.24)	−0.12 (−0.35, 0.12)	0.319	0.035	0.095
**n-3 : males**	5.56 (0.43)	5.77 (0.54)	+0.21 (0.014, 0.42)	0.036		0.116
**n-6 : females**	5.21 (0.46)	5.26 (0.53)	+0.062 (−0.17, 0.30)	0.600	0.967	
**n-6 : males**	5.53 (0.60)	5.62 (0.47)	+0.069 (−0.13, 0.27)	0.496		
**HbA1c, %**						
**n-3 : females**	5.48 (0.30)	5.51 (0.21)	+0.031 (−0.044, 0.11)	0.411	0.828	0.641
**n-3 : males**	5.45 (0.30)	5.49 (0.26)	+0.020 (−0.042, 0.083)	0.520		0.656
**n-6 : females**	5.48 (0.26)	5.49 (0.24)	+0.012 (−0.063, 0.088)	0.742	0.909	
**n-6 : males**	5.48 (0.26)	5.47 (0.24)	+0.007 (−0.056, 0.070)	0.830		
**Insulin, mU/L**						
**n-3 : females**	9.35 (8.34)	7.44 (5.08)	−2.48 (−4.61, −0.34)	0.023	0.003	0.005
**n-3 : males**	12.1 (7.90)	14.7 (7.54)	+1.78 (−0.035, 3.60)	0.055		0.634
**n-6 : females**	10.5 (5.38)	10.3 (6.74)	+0.41 (−1.72, 2.55)	0.702	0.500	
**n-6 : males**	13.8 (8.13)	14.3 (7.39)	+1.37 (−0.45, 3.19)	0.138		
**INCP, nmol/L**						
**n-3 : females**	0.63 (0.29)	0.59 (0.24)	−0.077 (−0.17, 0.013)	0.093	0.008	0.017
**n-3 : males**	0.84 (0.33)	0.95 (0.28)	+0.084 (0.007, 0.16)	0.032		0.209
**n-6 : females**	0.70 (0.21)	0.69 (0.32)	+0.032 (−0.058, 0.12)	0.486	0.948	
**n-6 : males**	0.90 (0.32)	0.90 (0.27)	+0.035 (−0.041, 0.11)	0.359		
**HOMA2-IR**						
**n-3 : females**	1.42 (0.69)	1.30 (0.55)	−0.18 (−0.40, 0.034)	0.097	0.008	0.013
**n-3 : males**	1.91 (0.77)	2.18 (0.63)	+0.21 (0.024, 0.39)	0.027		0.179
**n-6 : females**	1.55 (0.49)	1.57 (0.78)	+0.082 (−0.14, 0.30)	0.456	0.969	
**n-6 : males**	2.04 (0.77)	2.06 (0.63)	+0.088 (−0.098, 0.27)	0.350		
**HOMA2-%S**						
**n-3 : females**	85.6 (36.8)	88.7 (32.3)	+13.2 (2.94, 23.5)	0.012	<0.001	0.016
**n-3 : males**	60.1 (23.3)	49.1 (12.3)	−8.60 (−14.7, −2.47)	0.006		0.127
**n-6 : females**	70.8 (23.1)	77.3 (31.9)	+1.60 (−7.09, 10.3)	0.716	0.246	
**n-6 : males**	55.1 (18.0)	53.0 (16.3)	−4.68 (−10.9, 1.49)	0.136		
**HOMA2-%B**						
**n-3 : females**	107 (23.2)	108 (25.4)	−4.80 (−15.3, 5.69)	0.366	0.393	0.370
**n-3 : males**	118 (30.1)	123 (31.9)	+1.17 (−7.78, 10.1)	0.796		0.914
**n-6 : females**	118 (24.7)	112 (25.0)	−0.091 (−10.6, 10.4)	0.986	0.911	
**n-6 : males**	126 (31.6)	122 (25.5)	+0.69 (−8.26, 9.64)	0.879		
**QUICKI**						
**n-3 : females**	0.16 (0.017)	0.16 (0.015)	+0.007 (0.002, 0.013)	0.005	<0.001	0.001
**n-3 : males**	0.15 (0.014)	0.14 (0.009)	−0.005 (−0.008, −0.002)	0.002		0.151
**n-6 : females**	0.15 (0.011)	0.15 (0.015)	−0.001 (−0.006, 0.004)	0.704	0.452	
**n-6 : males**	0.14 (0.012)	0.14 (0.012)	−0.003 (−0.007, 0.000)	0.047		
**rQUICKI**						
**n-3 : females**	0.17 (0.030)	0.18 (0.028)	+0.007 (−0.004, 0.018)	0.208	0.406	0.108
**n-3 : males**	0.16 (0.023)	0.17 (0.029)	+0.001 (−0.008, 0.010)	0.841		0.712
**n-6 : females**	0.17 (0.022)	0.17 (0.029)	−0.001 (−0.012, 0.010)	0.847	0.952	
**n-6 : males**	0.17 (0.025)	0.16 (0.028)	−0.001 (−0.010, 0.009)	0.894		
**LP-IR**						
**n-3 : females**	28.6 (25.0)	27.6 (19.6)	−5.78 (−12.8, 1.22)	0.104	0.552	0.018
**n-3 : males**	57.9 (18.7)	46.8 (16.0)	−8.48 (−14.1, −2.88)	0.003		0.002
**n-6 : females**	38.1 (27.3)	34.5 (23.2)	+1.16 (−5.84, 8.15)	0.744	0.881	
**n-6 : males**	53.4 (18.0)	55.7 (14.5)	+0.48 (−5.12, 6.08)	0.866		
**Anthropometrics**						
**Body weight, kg**						
**n-3 : females**	80.4 (15.4)	80.2 (14.8)	−0.38 (−1.18, 0.43)	0.357	0.068	0.007
**n-3 : males**	96.7 (12.7)	96.9 (13.1)	+0.61 (−0.078, 1.29)	0.082		0.489
**n-6 : females**	80.8 (14.9)	81.4 (15.1)	+0.75 (−0.052, 1.56)	0.067	0.855	
**n-6 : males**	96.5 (13.7)	97.2 (13.0)	+0.85 (0.17, 1.53)	0.015		
**BMI, kg/m²**						
**n-3 : females**	28.4 (4.63)	28.4 (4.38)	−0.13 (−0.42, 0.16)	0.359	0.127	0.012
**n-3 : males**	30.0 (3.78)	30.1 (3.93)	+0.16 (−0.086, 0.41)	0.200		0.488
**n-6 : females**	28.6 (4.46)	28.8 (4.53)	+0.28 (−0.014, 0.56)	0.062	0.917	
**n-6 : males**	29.9 (4.08)	30.2 (3.95)	+0.26 (0.009, 0.50)	0.042		

frontiers

Variable and treatment	Baseline[2]	Follow-up[2]	Absolute change[3]	Time[4]	wTXbSEX[5]	bTXwSEX[6]
**Body fat mass, kg**						
**n-3 : females**	31.9 (11.0)	31.9 (10.4)	−0.15 (−0.92, 0.62)	0.706	0.148	0.048
**n-3 : males**	30.2 (10.6)	31.0 (10.4)	+0.58 (−0.082, 1.24)	0.085		0.834
**n-6 : females**	32.1 (10.4)	32.3 (11.2)	+0.71 (−0.060, 1.48)	0.070	0.915	
**n-6 : males**	30.5 (10.9)	31.4 (10.9)	+0.66 (−0.006, 1.32)	0.052		
**Body fat, %**						
**n-3 : females**	38.8 (5.98)	39.0 (5.45)	−0.18 (−0.92, 0.56)	0.636	0.205	0.214
**n-3 : males**	30.7 (6.89)	31.4 (6.48)	+0.43 (−0.20, 1.07)	0.180		0.873
**n-6 : females**	39.0 (5.32)	38.9 (6.22)	+0.34 (−0.40, 1.08)	0.370	0.931	
**n-6 : males**	30.9 (6.91)	31.6 (6.84)	+0.38 (−0.26, 1.02)	0.243		
**Waist circumference, cm**						
**n-3 : females**	99.4 (11.9)	96.8 (9.81)	−2.66 (−8.93, 3.60)	0.402	0.373	0.293
**n-3 : males**	106 (8.40)	106 (8.67)	+0.19 (−0.63, 1.01)	0.648		0.485
**n-6 : females**	99.5 (10.8)	100 (11.3)	+0.65 (−0.23, 1.53)	0.144	0.742	
**n-6 : males**	106 (8.84)	107 (8.32)	+0.46 (−0.28, 1.20)	0.218		
**Waist/hip ratio**						
**n-3 : females**	0.92 (0.059)	0.90 (0.079)	−0.016 (−0.062, 0.030)	0.492	0.399	0.439
**n-3 : males**	0.97 (0.037)	0.98 (0.043)	+0.004 (−0.005, 0.013)	0.359		0.453
**n-6 : females**	0.92 (0.060)	0.92 (0.058)	+0.002 (−0.005, 0.008)	0.630	0.927	
**n-6 : males**	0.98 (0.040)	0.98 (0.037)	+0.001 (−0.006, 0.008)	0.736		
**Waist/height ratio**						
**n-3 : females**	0.59 (0.064)	0.58 (0.054)	−0.016 (−0.053, 0.021)	0.406	0.379	0.294
**n-3 : males**	0.59 (0.047)	0.59 (0.050)	+0.001 (−0.004, 0.006)	0.660		0.481
**n-6 : females**	0.59 (0.058)	0.60 (0.061)	+0.004 (−0.001, 0.009)	0.130	0.694	
**n-6 : males**	0.59 (0.051)	0.59 (0.048)	+0.003 (−0.002, 0.007)	0.227		
**Visceral fat area, cm²**						
**n-3 : females**	140 (52.9)	136 (48.9)	−4.61 (−17.0, 7.80)	0.463	0.296	0.793
**n-3 : males**	182 (54.0)	189 (53.6)	+3.49 (−5.47, 12.4)	0.442		0.778
**n-6 : females**	141 (54.0)	138 (51.5)	−2.70 (−15.1, 9.71)	0.667	0.349	
**n-6 : males**	189 (63.6)	190 (65.2)	+4.56 (−4.40, 13.5)	0.315		
**Fat-free mass, kg**						
**n-3 : females**	48.5 (5.47)	48.4 (5.43)	+0.027 (−0.66, 0.71)	0.938	0.876	0.299
**n-3 : males**	66.2 (5.99)	66.0 (6.07)	+0.097 (−0.49, 0.68)	0.744		0.538
**n-6 : females**	48.6 (5.59)	49.0 (5.68)	+0.42 (−0.27, 1.10)	0.231	0.785	
**n-6 : males**	66.0 (6.27)	66.0 (5.87)	+0.29 (−0.29, 0.88)	0.324		
**Liver function markers, serum**						
**ALAT, U/L**						
**n-3 : females**	22.2 (14.8)	24.6 (17.4)	+3.10 (−0.83, 7.02)	0.121	0.860	0.982
**n-3 : males**	33.5 (14.9)	38.5 (15.9)	+2.59 (−1.54, 6.72)	0.217		0.265
**n-6 : females**	20.9 (8.80)	24.0 (14.9)	+3.07 (−0.55, 6.69)	0.096	0.375	
**n-6 : males**	37.5 (17.9)	36.5 (14.5)	+0.68 (−3.22, 4.58)	0.731		
**Albumin, g/L**						
**n-3 : females**	43.4 (2.22)	43.6 (3.05)	+0.34 (−0.85, 1.54)	0.570	0.436	0.670
**n-3 : males**	45.1 (1.73)	44.8 (2.24)	−0.28 (−1.29, 0.74)	0.591		0.928
**n-6 : females**	43.1 (2.29)	43.3 (2.63)	+0.094 (−1.10, 1.29)	0.877	0.601	
**n-6 : males**	45.0 (2.02)	44.8 (2.16)	−0.32 (−1.34, 0.70)	0.532		
**ALP, U/L**						
**n-3 : females**	69.3 (23.5)	66.9 (25.0)	−3.00 (−6.79, 0.79)	0.119	0.422	0.016
**n-3 : males**	68.4 (18.4)	65.3 (15.1)	−5.02 (−8.25, −1.79)	0.003		0.002
**n-6 : females**	70.4 (25.2)	72.2 (23.8)	+2.38 (−1.41, 6.16)	0.216	0.567	
**n-6 : males**	72.3 (19.0)	71.3 (17.2)	+0.93 (−2.30, 4.16)	0.569		
**ASAT, U/L**						
**n-3 : females**	22.0 (5.54)	23.1 (6.71)	+1.09 (−1.14, 3.33)	0.333	0.864	0.723
**n-3 : males**	28.1 (10.1)	28.8 (7.85)	+0.80 (−1.71, 3.31)	0.528		0.170
**n-6 : females**	21.9 (4.28)	23.4 (5.91)	+1.41 (−0.82, 3.64)	0.214	0.163	
**n-6 : males**	27.5 (7.74)	27.0 (6.65)	−0.97 (−3.48, 1.54)	0.445		
**Bile acids, µmol/L**						
**n-3 : females**	2.22 (1.28)	2.41 (1.20)	+0.025 (−0.99, 1.04)	0.961	0.820	0.651
**n-3 : males**	3.96 (2.98)	3.73 (1.83)	+0.19 (−0.86, 1.25)	0.718		0.385
**n-6 : females**	2.54 (1.12)	2.56 (1.50)	+0.18 (−0.83, 1.19)	0.723	0.569	
**n-6 : males**	3.11 (1.40)	4.14 (2.46)	+0.60 (−0.45, 1.66)	0.261		
**Bilirubin, µmol/L**						
**n-3 : females**	6.12 (2.50)	6.44 (1.86)	+0.87 (−0.16, 1.91)	0.098	0.176	0.025
**n-3 : males**	10.3 (4.70)	9.32 (3.66)	−0.24 (−1.46, 0.99)	0.703		0.044
**n-6 : females**	5.44 (1.50)	5.62 (1.71)	+0.005 (−0.92, 0.93)	0.991	0.052	
**n-6 : males**	9.14 (3.97)	8.18 (3.27)	−1.44 (−2.57, −0.31)	0.013		

Variable and treatment	Baseline[2]	Follow-up[2]	Absolute change[3]	Time[4]	wTXbSEX[5]	bTXwSEX[6]
**CK, U/L**						
**n-3 : females**	76.2 (27.9)	74.9 (29.0)	−1.43 (−23.8, 20.9)	0.899	0.375	0.438
**n-3 : males**	153 (81.5)	128 (54.8)	−17.5 (−45.4, 10.4)	0.216		0.835
**n-6 : females**	76.4 (34.1)	91.3 (75.9)	+15.0 (−31.8, 61.9)	0.527	0.187	
**n-6 : males**	132 (61.6)	126 (51.5)	−19.6 (−41.2, 2.11)	0.076		
**GGT, U/L[7]**						
**n-3 : females**	18.5 (7.50)	17.3 (6.85)	−1.91 (−5.34, 1.52)	0.273	0.256	0.313
**n-3 : males**	41.2 (35.1)	43.4 (41.8)	+0.68 (−2.22, 3.58)	0.643		0.767
**n-6 : females**	19.7 (10.3)	19.5 (11.5)	+0.43 (−4.86, 5.71)	0.873	0.926	
**n-6 : males**	45.0 (46.0)	44.1 (40.0)	+0.10 (−4.36, 4.56)	0.963		
**LD, U/L**						
**n-3 : females**	156 (21.6)	156 (18.4)	+2.69 (−6.09, 11.5)	0.545	0.795	0.813
**n-3 : males**	154 (18.7)	154 (16.9)	+1.17 (−6.29, 8.64)	0.756		0.923
**n-6 : females**	151 (15.6)	157 (18.5)	+4.00 (−4.78, 12.8)	0.368	0.684	
**n-6 : males**	152 (16.8)	155 (21.4)	+1.63 (−5.83, 9.09)	0.666		
**Additional hormones, serum**						
**Estrogen, pmol/L[7]**						
**n-3 : females**	143 (123)	113 (89.7)	−37.2 (−75.5, 1.16)	0.057	0.166	0.535
**n-3 : males**	116 (34.0)	113 (31.4)	−9.15 (−20.2, 1.87)	0.103		0.037
**n-6 : females**	170 (185)	181 (275)	−14.5 (−87.1, 58.1)	0.694	0.444	
**n-6 : males**	123 (26.5)	128 (37.7)	+2.80 (−6.47, 12.1)	0.550		
**Testosterone, nmol/L**						
**n-3 : females**	0.80 (0.39)	0.81 (0.41)	−0.041 (−0.14, 0.062)	0.432	0.464	0.322
**n-3 : males**	14.2 (3.96)	15.0 (4.48)	+0.47 (−0.91, 1.86)	0.500		0.736
**n-6 : females**	0.89 (0.54)	0.84 (0.43)	−0.006 (−0.11, 0.097)	0.912	0.834	
**n-6 : males**	14.9 (4.07)	14.7 (4.00)	+0.14 (−1.24, 1.52)	0.840		
**SHBG, nmol/L**						
**n-3 : females**	57.5 (27.9)	56.9 (23.4)	−0.84 (−9.84, 8.17)	0.854	0.593	0.461
**n-3 : males**	37.3 (16.6)	39.1 (18.5)	+1.70 (−1.00, 4.41)	0.214		0.231
**n-6 : females**	57.7 (27.5)	61.3 (28.5)	+3.77 (−5.23, 12.8)	0.408	0.448	
**n-6 : males**	37.5 (15.4)	37.6 (15.7)	+0.16 (−2.54, 2.86)	0.907		
**FAI**						
**n-3 : females**	1.53 (0.76)	1.49 (0.59)	−0.069 (−0.32, 0.18)	0.584	0.641	0.847
**n-3 : males**	43.6 (19.1)	43.2 (16.3)	−1.11 (−5.51, 3.29)	0.618		0.572
**n-6 : females**	1.60 (0.58)	1.48 (0.66)	−0.091 (−0.34, 0.16)	0.474	0.750	
**n-6 : males**	45.1 (19.6)	45.0 (20.8)	+0.62 (−3.78, 5.02)	0.780		
**FSH, IU/L**						
**n-3 : females**	40.8 (29.7)	46.9 (30.2)	2.63 (−5.16, 10.4)	0.504	0.531	0.359
**n-3 : males**	5.23 (3.54)	5.35 (3.73)	0.033 (−2.56, 2.62)	0.980		0.167
**n-6 : females**	45.4 (34.8)	49.3 (33.5)	5.56 (2.16, 8.96)	0.002	0.023	
**n-6 : males**	5.41 (3.68)	5.84 (4.56)	0.52 (−2.18, 3.21)	0.704		
**TSH, mU/L**						
**n-3 : females**	1.64 (0.58)	1.83 (0.80)	+0.035 (−0.28, 0.35)	0.826	0.586	0.869
**n-3 : males**	1.77 (0.76)	1.92 (0.93)	+0.15 (−0.11, 0.40)	0.261		0.440
**n-6 : females**	1.94 (1.23)	1.80 (0.73)	+0.011 (−0.30, 0.32)	0.945	0.900	
**n-6 : males**	1.78 (0.83)	1.81 (0.84)	+0.037 (−0.22, 0.29)	0.777		
**FT4, pmol/L**						
**n-3 : females**	15.1 (2.44)	15.7 (2.92)	+0.45 (−0.31, 1.21)	0.241	0.341	0.110
**n-3 : males**	16.2 (1.80)	16.4 (1.76)	−0.030 (−0.68, 0.62)	0.928		0.580
**n-6 : females**	15.4 (1.97)	15.0 (2.40)	−0.20 (−0.96, 0.56)	0.609	0.963	
**n-6 : males**	16.6 (2.17)	16.2 (2.37)	−0.22 (−0.87, 0.43)	0.502		
**IGF-1, nmol/L**						
**n-3 : females**	17.2 (4.91)	17.9 (3.66)	+0.78 (−0.91, 2.48)	0.361	0.468	0.006
**n-3 : males**	20.5 (6.50)	22.7 (9.05)	+2.04 (−0.90, 4.97)	0.172		0.189
**n-6 : females**	16.9 (3.45)	15.9 (3.50)	−1.15 (−2.72, 0.43)	0.152	0.301	
**n-6 : males**	29.6 (41.1)	20.7 (7.47)	+0.23 (−1.87, 2.34)	0.827		
**Vitamin D_3, nmol/L**						
**n-3 : females**	70.5 (18.8)	69.5 (22.1)	−1.38 (−7.79, 5.02)	0.669	0.099	0.443
**n-3 : males**	63.5 (18.2)	56.8 (15.4)	−8.09 (−12.8, −3.33)	0.001		0.869
**n-6 : females**	71.2 (14.4)	66.4 (18.6)	−4.47 (−10.9, 1.93)	0.169	0.410	
**n-6 : males**	66.7 (21.5)	57.0 (17.1)	−7.79 (−12.6, −3.03)	0.002		
**Ketone bodies, serum**						
**3-hydroxybutyrate, µmol/L**						
**n-3 : females**	92.7 (80.2)	97.9 (86.6)	+16.5 (−24.5, 57.5)	0.427	0.107	0.410
**n-3 : males**	101 (91.0)	53.1 (33.3)	−27.8 (−62.7, 7.07)	0.117		0.446
**n-6 : females**	63.1 (39.8)	77.9 (96.8)	+5.46 (−35.8, 46.7)	0.793	0.360	
**n-6 : males**	68.1 (62.8)	61.8 (64.6)	−19.5 (−53.9, 14.9)	0.263		

Variable and treatment	Baseline[2]	Follow-up[2]	Absolute change[3]	Time[4]	wTXbSEX[5]	bTXwSEX[6]
**Acetoacetate, µmol/L**						
**n-3 : females**	44.4 (29.5)	49.7 (36.0)	+10.3 (−8.36, 29.0)	0.275	0.136	0.314
**n-3 : males**	58.2 (38.0)	42.6 (28.9)	−7.69 (−22.4, 7.04)	0.303		0.292
**n-6 : females**	34.3 (17.5)	41.0 (37.8)	+1.60 (−17.1, 20.3)	0.865	0.230	
**n-6 : males**	41.4 (32.1)	37.4 (21.9)	−12.9 (−27.6, 1.84)	0.086		

[1] Fasting blood levels were analyzed with cLMMs adjusted for the main effects of period and subject-averaged baselines. Abbreviations: ALAT, alanine aminotransferase; ALP, alkaline phosphatase; ASAT, aspartate aminotransferase; bTXwSEX, between-treatment within-sex; CK, creatine kinase; FAI, free androgen index; FSH, follicle-stimulating hormone; FT4, free thyroxine; GGT, γ-glutamyl transpeptidase; HbA1c, glycated hemoglobin; HOMA2-IR, homeostasis model assessment of insulin resistance index 2 (computer model); HOMA2-%B, homeostasis model assessment of β-cell function index 2 (computer model); HOMA2-%S, homeostasis model assessment of insulin sensitivity index 2 (computer model); IGF-1, insulin-like growth factor-1; INCP, insulin C-peptide; LD, lactate dehydrogenase; cLMM, constrained linear mixed-effects model; LP-IR, lipoprotein-based insulin resistance index; n-3, omega-3 PUFAs; n-6, omega-6 PUFAs; PUFAs, polyunsaturated fatty acids; QUICKI, quantitative insulin sensitivity check index; rQUICKI, revised QUICKI; SHBG, sex hormone-binding globulin; TSH, thyroid-stimulating hormone; wTXbSEX, within-treatment between-sex.

[2] Values are arithmetic means (SDs) of fasting blood levels at baseline and follow-up within treatments and sexes.

[3] Absolute model-adjusted mean change scores (95% CIs) from baseline to follow-up.

[4] P-values for absolute changes from baseline to follow-up within treatments and sexes (time effects).

[5] P-values for absolute changes from baseline to follow-up within treatments and between sexes (sex differences in time effects). The first and second values refer to between-sex differences after the n-3 and n-6 interventions, respectively.

[6] P-values for absolute changes from baseline to follow-up between treatments and within sexes (group differences in time effects within each stratum of sex). The first and second values refer to between-treatment differences in females and males, respectively.

[7] Two influential outliers were excluded from the final analyses of GGT and estrogen (different cases).

frontiers

SUPPLEMENTARY FIGURES

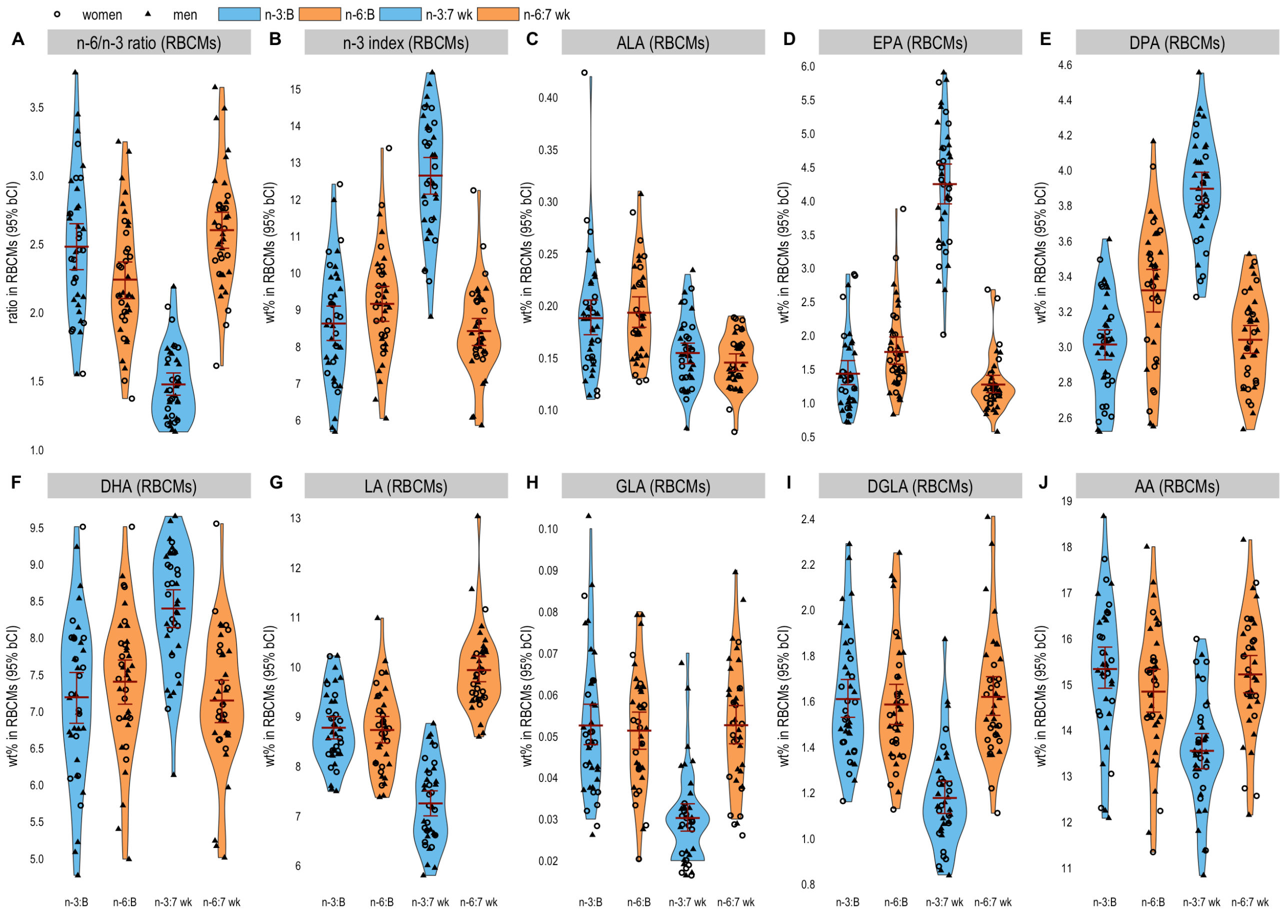

SUPPLEMENTARY FIGURE 1 Distribution of data points for RBCM fatty acids
The violin plots show the distribution of individual data points measured at the four baseline (blue violins) and follow-up (orange violins) study visits, while the red error bar plots (within the violins) display the mean values and bootstrapped 95% confidence intervals. Abbreviations: AA, arachidonic acid; ALA, α-linolenic acid; B, baseline visit; DGLA, dihomo-γ-linolenic acid; DPA, docosapentaenoic acid; GLA, γ-linolenic acid; LA, linoleic acid; n-3, omega-3 PUFAs; n-6, omega-6 PUFAs; PUFAs, polyunsaturated fatty acids; RBCMs, red blood cell membranes; wt%, weight percentage of total fatty acids; 7 wk, follow-up visit after 7 weeks; 95% bCI, bootstrapped 95% confidence interval.

frontiers

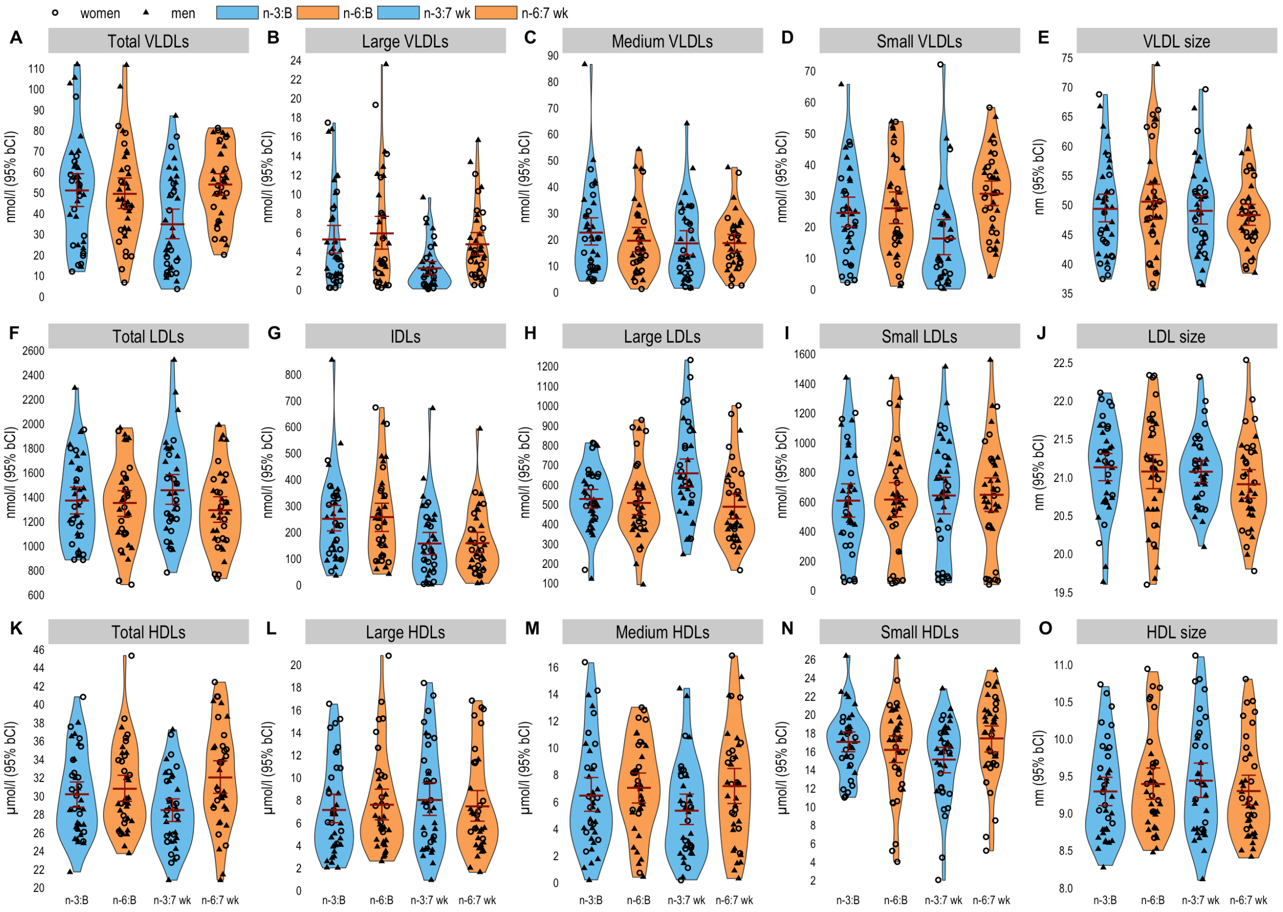

SUPPLEMENTARY FIGURE 2 Distribution of data points for lipoprotein subfractions
The violin plots show the distribution of individual data points measured at the four baseline (blue violins) and follow-up (orange violins) study visits, while the red error bar plots (within the violins) display the mean values and bootstrapped 95% confidence intervals. Abbreviations: B, baseline visit; HDLs, HDL particles; IDLs, intermediate-density lipoprotein particles; LDLs, LDL particles; n-3, omega-3 PUFAs; n-6, omega-6 PUFAs; PUFAs, polyunsaturated fatty acids; VLDLs, VLDL particles; 7 wk, follow-up visit after 7 weeks; 95% bCI, bootstrapped 95% confidence interval. Total VLDLs and large VLDLs also include chylomicrons if present.

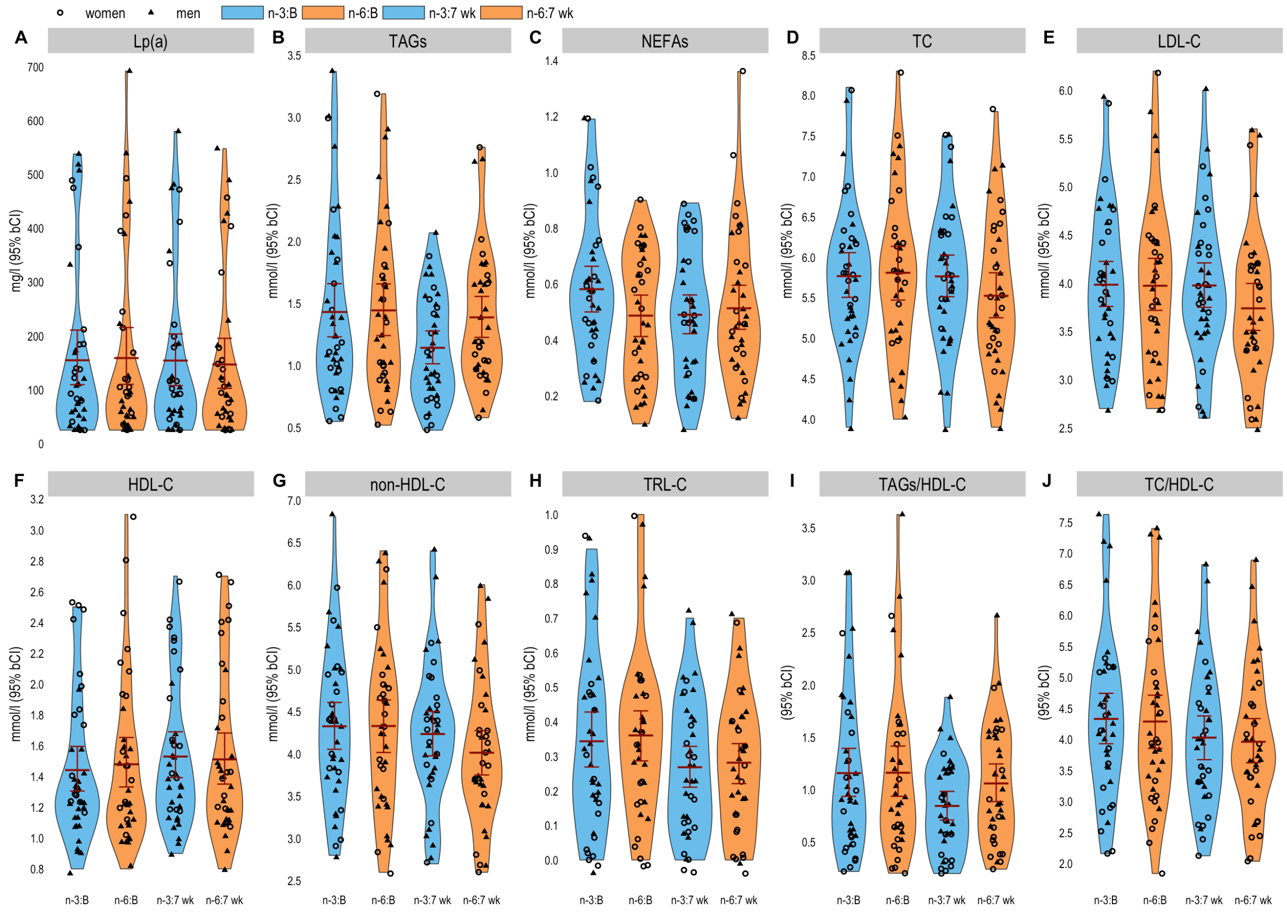

SUPPLEMENTARY FIGURE 3 Distribution of data points for Lp(a) and blood lipids
The violin plots show the distribution of individual data points measured at the four baseline (blue violins) and follow-up (orange violins) study visits, while the red error bar plots (within the violins) display the mean values and bootstrapped 95% confidence intervals. Abbreviations: B, baseline visit; HDL-C, HDL cholesterol; LDL-C, LDL cholesterol; Lp(a), lipoprotein (a); n-3, omega-3 PUFAs; n-6, omega-6 PUFAs; NEFAs, non-esterified fatty acids; non-HDL-C, non-HDL cholesterol; PUFAs, polyunsaturated fatty acids; TAGs, triacylglycerols; TC, total cholesterol; TRL-C, TAG-rich lipoprotein cholesterol; 7 wk, follow-up visit after 7 weeks; 95% bCI, bootstrapped 95% confidence interval.

frontiers

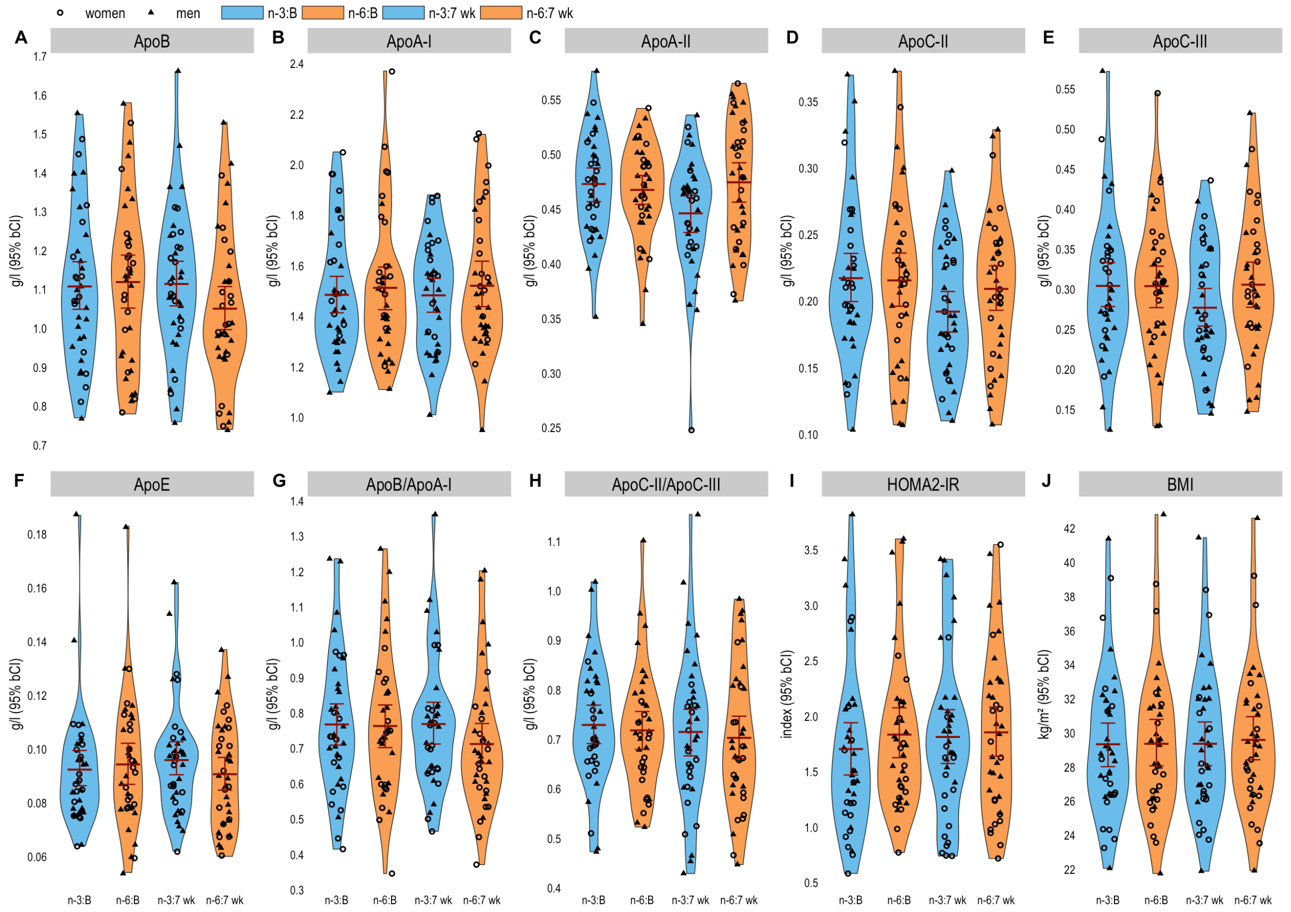

SUPPLEMENTARY FIGURE 4 Distribution of data points for apolipoproteins, HOMA2-IR, and BMI

The violin plots show the distribution of individual data points measured at the four baseline (blue violins) and follow-up (orange violins) study visits, while the red error bar plots (within the violins) display the mean values and bootstrapped 95% confidence intervals. Abbreviations: Apo, apolipoprotein; B, baseline visit; HOMA2-IR, homeostasis model assessment of insulin resistance index 2 (computer model); n-3, omega-3 PUFAs; n-6, omega-6 PUFAs; PUFAs, polyunsaturated fatty acids; 7 wk, follow-up visit after 7 weeks; 95% bCI, bootstrapped 95% confidence interval.

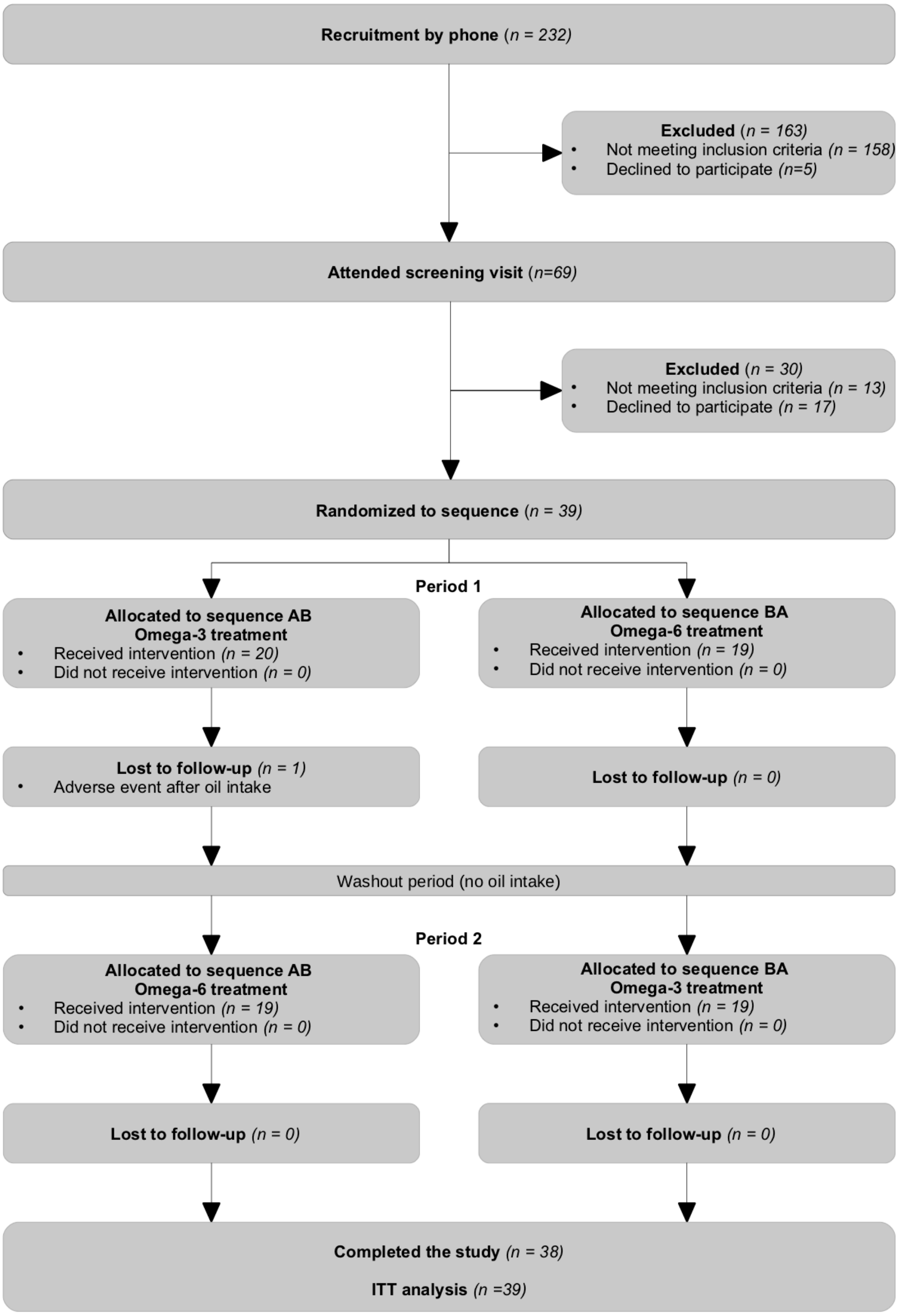

SUPPLEMENTARY FIGURE 5 Flow diagram of the study participants
The diagram shows the numbers of participants screened, included, allocated to sequences, and analyzed in the present study (CONSORT 2019 format (2)). 40 accepted the invitation to participate in the study, but one male dropped out for personal reasons before the first baseline visit and randomization. Another male, assigned to sequence AB, was lost to follow-up after one month in the first period due to adverse events of nausea after intake of the n-3 oil (1).

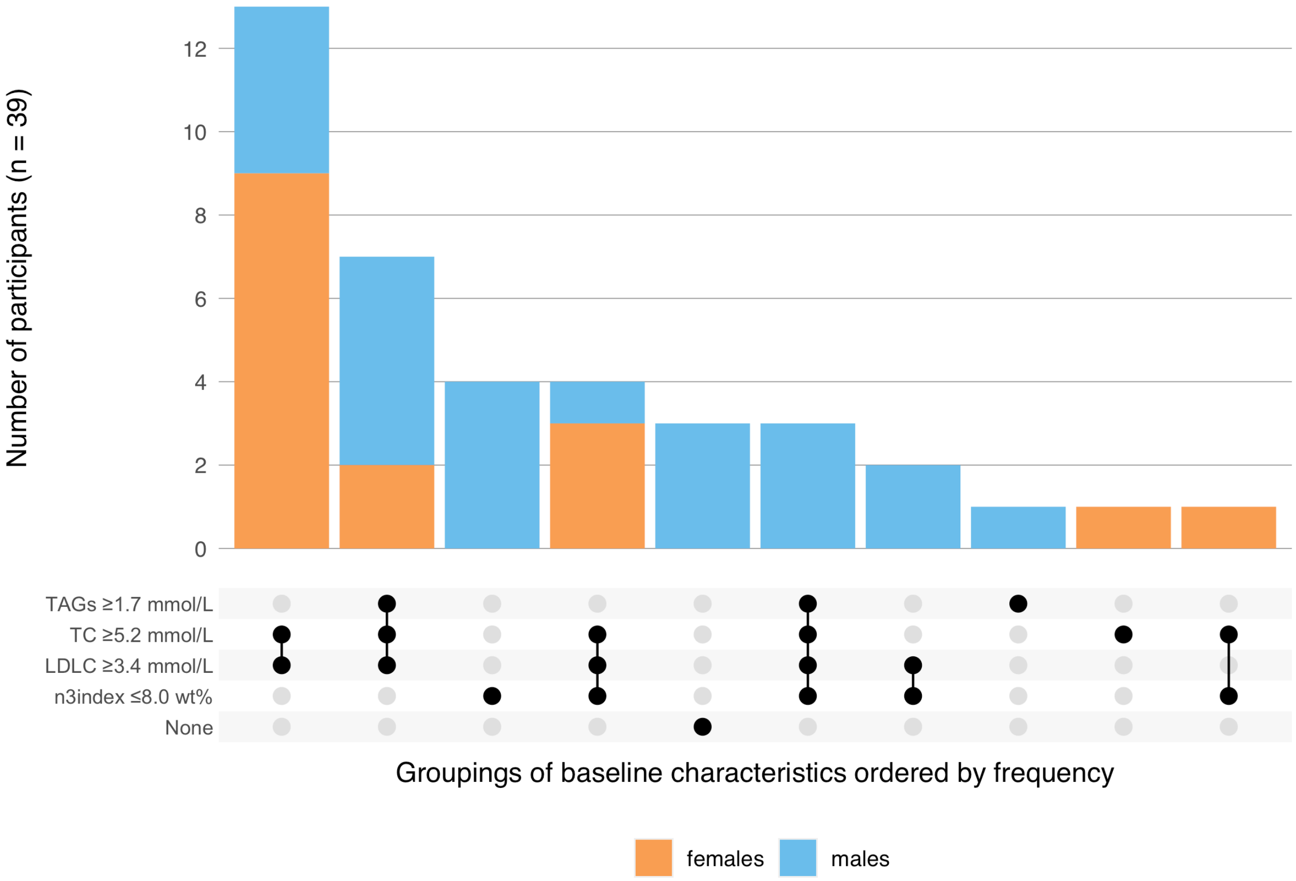

SUPPLEMENTARY FIGURE 6 Lipid levels at baseline
The upset plot shows the co-occurrence of hyperlipidemic traits at baseline for females and males separately. The circulating lipid levels for borderline high hyperlipidemic individuals are according to the American College of Cardiology/American Heart Association guidelines (3, 4). Abbreviations: LDLC, LDL cholesterol; n3index, n-3 index (weight percent of EPA and DHA in red blood cell membranes); TAGs, triacylglycerols; TC, total cholesterol.

REFERENCES

1. Grytten E, Laupsa-Borge J, Bohov P, Bjørndal B, Strand E, Skorve J, et al. Changes in Lipoprotein Particle Subclasses, Standard Lipids, and Apolipoproteins after Supplementation with N-3 or N-6 Pufas in Abdominal Obesity: A Randomized Double-Blind Crossover Study. *Clin Nutr* (2021) 40(5):2556-75. Epub 2021/05/03. doi: 10.1016/j.clnu.2021.03.040.

2. Dwan K, Li T, Altman DG, Elbourne D. Consort 2010 Statement: Extension to Randomised Crossover Trials. *BMJ* (2019) 366:l4378. doi: 10.1136/bmj.l4378.

3. Miller M, Stone NJ, Ballantyne C, Bittner V, Criqui MH, Ginsberg HN, et al. Triglycerides and Cardiovascular Disease: A Scientific Statement from the American Heart Association. *Circulation* (2011) 123(20):2292-333. doi: 10.1161/CIR.0b013e3182160726.

4. Grundy SM, Stone NJ, Bailey AL, Beam C, Birtcher KK, Blumenthal RS, et al. 2018 Aha/Acc/Aacvpr/Aapa/Abc/Acpm/Ada/Ags/Apha/Aspc/Nla/Pcna Guideline on the Management of Blood Cholesterol: A Report of the American College of Cardiology/American Heart Association Task Force on Clinical Practice Guidelines. *Circulation* (2019) 139(25):e1082-e143. doi: 10.1161/CIR.0000000000000625.
